# Single-cell long-read sequencing-based mapping reveals specialized splicing patterns in developing and adult mouse and human brain

**DOI:** 10.1038/s41593-024-01616-4

**Published:** 2024-04-09

**Authors:** Anoushka Joglekar, Wen Hu, Bei Zhang, Oleksandr Narykov, Mark Diekhans, Jordan Marrocco, Jennifer Balacco, Lishomwa C. Ndhlovu, Teresa A. Milner, Olivier Fedrigo, Erich D. Jarvis, Gloria Sheynkman, Dmitry Korkin, M. Elizabeth Ross, Hagen U. Tilgner

**Affiliations:** 1 Feil Family Brain and Mind Research Institute, Weill Cornell Medicine, New York, NY USA; 2 Center for Neurogenetics, Weill Cornell Medicine, New York, NY USA; 3 Spatial Genomics, Inc., Pasadena, CA USA; 4 Bioinformatics and Computational Biology Program, Worcester Polytechnic Institute, Worcester, MA USA; 5 Computer Science Department, Worcester Polytechnic Institute, Worcester, MA USA; 6 Data Science Program, Worcester Polytechnic Institute, Worcester, MA USA; 7 UC Genomics Institute, University of California, Santa Cruz, Santa Cruz, CA USA; 8 Department of Biology, Touro University, New York, NY USA; 9 Laboratory of Neuroendocrinology, The Rockefeller University, New York, NY USA; 10 Vertebrate Genome Lab, The Rockefeller University, New York, NY USA; 11 Department of Medicine, Division of Infectious Diseases, Weill Cornell Medicine, New York, NY USA; 12 Laboratory of Neurogenetics of Language, The Rockefeller University, New York, NY USA; 13 Howard Hughes Medical Institute, Chevy Chase, MD USA; 14 Department of Molecular Physiology and Biological Physics, University of Virginia, Charlottesville, VA USA; 15 Department of Biochemistry and Molecular Genetics, University of Virginia, Charlottesville, VA USA; 16 Center for Public Health Genomics, University of Virginia, Charlottesville, VA USA; 17 UVA Comprehensive Cancer Center, University of Virginia, Charlottesville, VA USA; 18 Present Address: New York Genome Center, New York, NY USA

**Keywords:** Genetics of the nervous system, RNA splicing, Computational models

## Abstract

RNA isoforms influence cell identity and function. However, a comprehensive brain isoform map was lacking. We analyze single-cell RNA isoforms across brain regions, cell subtypes, developmental time points and species. For 72% of genes, full-length isoform expression varies along one or more axes. Splicing, transcription start and polyadenylation sites vary strongly between cell types, influence protein architecture and associate with disease-linked variation. Additionally, neurotransmitter transport and synapse turnover genes harbor cell-type variability across anatomical regions. Regulation of cell-type-specific splicing is pronounced in the postnatal day 21-to-postnatal day 28 adolescent transition. Developmental isoform regulation is stronger than regional regulation for the same cell type. Cell-type-specific isoform regulation in mice is mostly maintained in the human hippocampus, allowing extrapolation to the human brain. Conversely, the human brain harbors additional cell-type specificity, suggesting gain-of-function isoforms. Together, this detailed single-cell atlas of full-length isoform regulation across development, anatomical regions and species reveals an unappreciated degree of isoform variability across multiple axes.

## Abstract (web summary)

RNA alternative splicing is involved in determining cell identity, but a comprehensive molecular map is missing. Here, the authors provide a human and mouse brain atlas of transcript isoforms linking them to cellular identity, brain regions and development stages.

## Main

Transcriptomic studies have offered insight into the molecular makeup of single cells in complex tissues such as the brain^1–4^ and perturbations in neurological diseases^5,6^. However, few brain single-cell studies consider mRNA isoforms. mRNA isoforms are strongly modulated in the mammalian brain and influence processes such as cellular growth^7^, maturation^8–11^, migration^12,13^, synapse formation^14,15^ and activity patterns^16–20^. These properties of neuronal and nonneuronal cells are altered in development and are highly distinct between brain regions, which may underlie regional vulnerabilities in disease. Although tissue-specific splicing has evolved across species^21–23^, we know little about the cellular isoform diversity in the brain.

We and others have developed various single-cell short-read^24–26^ and long-read^27–31^ technologies to study splicing. Long-read cDNA sequencing can quantify isoforms within and between conditions for thousands of single cells^28,32–35^. In the mouse brain, isoforms can define embryonic cell types^27,28^, postnatal cell subtypes and, to some extent, brain regions^32^. However, the extent to which brain regions differ in isoform expression for matched cell types is unknown. Furthermore, whether these regional differences differ in development or between cell subtypes is not well understood. Last, the degree to which any brain region- or cell-type-specific isoform patterns are transient or maintained across development is an unsolved question.

Here, using an enhanced single-cell long-read method (ScISOr-Seq2), we investigate these questions comprehensively. We investigate full-length isoforms across the following three axes: adult brain regions, cell subtypes and developmental time points. We find that neuron subtypes and glia show widespread isoform variability along these three axes. Thalamic and cerebellar astrocytes exhibit especially strong transcription start site (TSS), polyadenylation (poly(A)) site and exon regulation. Distinct exon sets display extremely high inclusion variability across cell types, brain regions and developmental time points, and mouse cell-type-specific splicing is conserved in humans. A strong splicing shift occurs in the hippocampal and cortical oligodendrocyte lineage after gene expression signatures distinguish oligodendrocyte precursors from astrocytes. Fluctuations in splicing variation occur during mouse adolescence, a critical period for splicing variability across all major cell types. A peak of neuronal subtype variability occurs in the telencephalon during this time. These data showcase the importance of long reads to capture a fuller picture of transcriptomic diversity in the brain and are available at www.isoformAtlas.com.

## Results

### Single-cell RNA sequencing identifies heterogenous cell populations

**Fig. 1:**
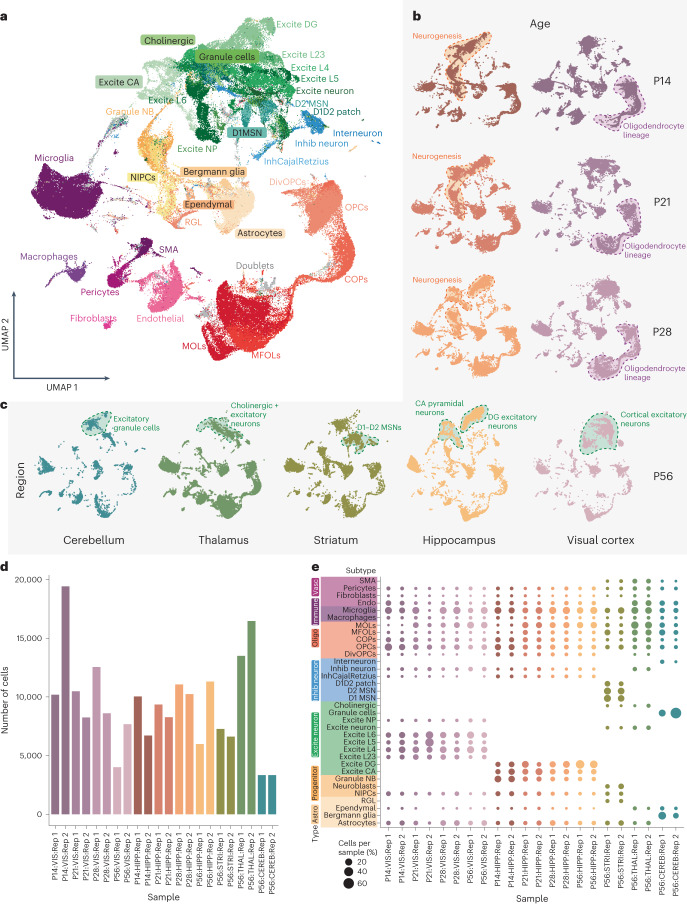
Summary of mouse brain cell subtype assignments by age and region. **a**, UMAP embedding of all ~200,000 cells. Each dot represents a cell that is colored according to its cell type of origin based on marker gene annotation; Excite, excitatory; DG, dentate gyrus; L, layer; Inhib, inhibitory; MOLs, mature oligodendrocytes; MFOLs, myelin-forming oligodendrocytes; Granule NB, granule neuroblasts; SMA, smooth muscle actin cells; COPs, committed oligodendrocyte precursors; NIPCs, neuronal intermediate progenitor cells; RGL, radial–glia like cells; NP, neural progenitors; D1 MSN, D1 medium spiny neuron; D2 MSN, D2 medium spiny neuron; D1D2 patch, Patch D1 and D2 striatal; DivOPCs, dividing OPCs; InhCajalRetzius, inhibitory Cajal–Retzius cells. **b**, Same UMAP representation from **a** but split by time point for the hippocampal (browns) and visual cortex (purples) lineage. **c**, Same UMAP representation from **a** but split by the region of origin at P56; blue, cerebellum; green, thalamus; olive, striatum; yellow, hippocampus; lilac, visual cortex. **d**, Bar plot depicting the number of cells obtained from each single-cell experiment (11 samples × 2 biological replicates); VIS, visual cortex; HIPP, hippocampus; STRI, striatum; THAL, thalamus; CEREB, cerebellum. **e**, Dot plot showing the percentage of cells belonging to each cell subtype indicated on the *y* axis obtained from the samples on the *x* axis. The color of the dots indicates sample of origin, and the size of the dot indicates the percentage of cells belonging to a subtype per sample; Vasc, vascular; Endo, endothelial; Astro, astrocytes; Oligo, oligodendrocytes; Rep, replicate.

Based on our single-cell/single-nucleus isoform sequencing^27,29^ studies, we devised ScISOr-Seq2 (Methods) to investigate brain region-specific, cell-type-specific and developmental-stage-specific isoform regulation. Given widespread transcription-mediated cell identity establishment in the telencephalon during postnatal development, we obtained single-cell 10x transcriptomics data from the mouse hippocampus and visual cortex at postnatal days 14, 21, 28 and 56 (P14, P21, P28 and P56, respectively; *n* = 16 samples, 2 biological replicates/age × 2 brain regions). Additionally, we obtained similar data from the adult (that is, P56) striatum, thalamus and cerebellum (*n* = 6 samples, 2 biological replicates/brain region × 3 brain regions). Filtering, quality control, short-read analysis^36,37^ and integration-mediated batch effect control^38^ yielded 204,725 cells (mean = 9,300 cells per sample; Methods and Supplementary Table 1), which we classified into neuronal, glial, vascular and immune cells. We defined three granularity levels for each cell: (1) broad, for example, neurons versus glia, (2) medium, for example, excitatory versus inhibitory neurons, and (3) subtype, for example, layer 2/3 versus layer 6 excitatory neurons (Fig. 1a). Time point-specific uniform manifold approximation and projection (UMAP) embeddings described hippocampal neurogenesis and oligodendrocyte maturation during adolescence (Fig. 1b). Similarly, mouse P56 brain region-specific UMAPs yielded broadly replicable cell types, albeit with region-specific neuronal populations (Fig. 1c). Gene expression of glial, immune and vascular cells was more homogeneous across brain regions (Fig. 1a,c and Supplementary Fig. 1). Except for the cerebellum, total cell numbers and cell subtypes were broadly consistent between samples (see Supplementary Note 1 and Fig. 1d,e).

### Cell types are characterized by distinct isoform regulation

Long-read data generated with Oxford Nanopore Technology (ONT) and PacBio HiFi sequencing yielded 250 × 10^6^ and 38 × 10^6^ barcoded long reads, respectively (Methods and Supplementary Tables 2 and 3). These were further processed to assign uniquely identified, multiexonic transcripts to single cells (Supplementary Figs. 2a and 3 and Supplementary Notes 2 and 3).

**Fig. 2:**
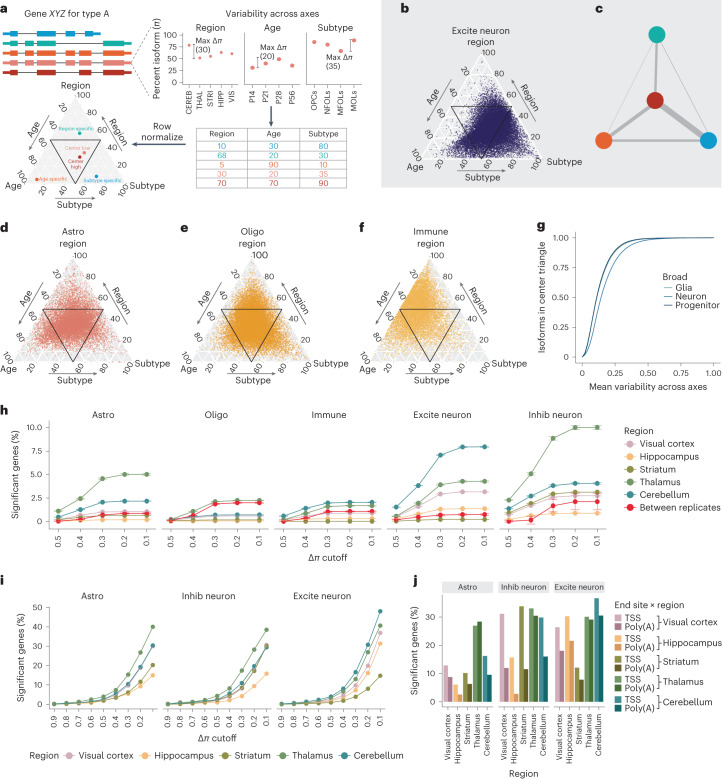
Distinct sources contribute to cell-type- and brain region-specific isoform expression in the mouse brain. **a**, Outline of full-length isoform variability across developmental age, brain region and cell subtypes. **b**, Ternary plot of variability in three axes for excitatory neurons. **c**, Network diagram of genes with isoforms in more than one triangle. The thickness of the lines represents the number of such genes. **d**–**f**, Same as **b** for astrocytes (**d**), oligodendrocytes (**e**) and immune cells (**f**). **g**, Comparison of mean variability for three broad cell types. **h**, Percentage of genes showing significant differences in isoform expression after repeatedly (*n* = 100) downsampling astrocyte reads of one brain region versus astrocytes of all other brain regions at five Δ*Π* cutoffs. The red curve represents the average percentage of significant genes when comparing two biological replicates of astrocytes within one brain region (*n* = 100 downsampling experiments averaged across *N* = 5 brain regions; left). The neighboring four plots depict the same for oligodendrocytes and immune cells and excitatory and inhibitory neurons. **i**, Same as in **h** but without downsampling for cell types indicated on the *x* axis. **j**, Percentage of genes with significant differences in TSS/poly(A) site choice for astrocytes of a fixed brain region versus astrocytes of all other brain regions at Δ*Π* ≥ 0.1 (left). The neighboring plots depict the same for inhibitory (Inhib neuron) and excitatory (Excite neuron) neurons.

To understand the roles of (1) developmental age, (2) brain region and (3) cell subtypes in splicing programs for each cell type, we calculated isoform variability of each cell type across these three axes (Methods). Similar to TSS–exon–poly(A) site contributions in ENCODE^39^, we represented the normalized age–subtype–region variability in a ternary plot. Each vertex of the triangle shows directed enrichment for the indicated axis of variability. The ‘center triangle’ represents isoforms with broadly equal variability along all three axes (Fig. 2a).

Excitatory neurons and their subtypes were well represented in all samples. Isoform variability was strongest across subtypes and less so across regions and time points (Fig. 2b). Considering genes with two isoforms in distinct triangles, indicating isoform-specific regulation rather than overall gene regulation, we found enrichment for specific patterns. We constructed a network diagram with nodes representing axes and edge weights indicating the number of such genes. For excitatory neurons, strong variation across subtypes for one isoform was frequently accompanied by uniform variation across all three axes for at least another isoform (Fig. 2c and Supplementary Fig. 4a).

Progenitor cell isoforms also varied more strongly by subtype than by age, suggesting that the switch from neuronal intermediate progenitor cells to granule neuroblasts is associated with isoform-mediated establishment of cell identity, regardless of developmental age. As progenitors are less abundant in nonhippocampal regions, there is little variation in progenitor isoforms between brain regions at P56 (Supplementary Fig. 4b). In contrast to excitatory neurons, isoform variability in inhibitory neurons was strong between ages and brain regions (Supplementary Fig. 4c). Among glia, both astrocytes and oligodendrocytes showed complex variability patterns along regions, ages and subtypes, but oligodendrocytes had stronger variability across regions and subtypes (Fig. 2d,e). Microglia dominated the immune population, where little variability between subtypes was observed. However, strong regional isoform variability characterized immune cells (Fig. 2f and Supplementary Fig. 4d). These observations were robust when increasing the minimum threshold of reads per gene from 10 to 100 (Supplementary Fig. 4e).

Center triangle isoforms have similar variability across all three axes, with variabilities being either all high or all low (Fig. 2a). For excitatory neurons, most center triangle isoforms had consistent low variability; however, a few showed consistent high variability (Supplementary Fig. 4f). Additionally, isoforms in the center triangle display subtype specificity, mirroring the trend in the entire excitatory neuron population (Supplementary Fig. 4g). A similar observation was made across all cell types (Supplementary Fig. 4h). In summary, cell types exhibit distinct preferences for isoform variability across brain regions, ages and cell subtypes.

Isoforms had higher mean variability across the three axes in neurons than in glia or progenitors, pointing to more complex neuronal regulation (Fig. 2g). Approximately 71.9% of genes had isoforms in distinct triangles and thus showed isoform variability independent of gene variability along at least one axis and cell type. Moreover, 48.14% of genes showed isoforms in three triangles, revealing isoform variability along two or three axes. However, this analysis does not include the comparison of isoform patterns of individual genes between cell types described above. We found that at least 36.6% of genes showed variable isoform usage between cell types in addition to regulation along another axis (Methods and Supplementary Fig. 5a). An investigation of individual cell types revealed a unique pattern of regulation in excitatory neurons, and some genes emerged as being hypervariable across multiple cell types (Methods, Supplementary Figs. 4i, 5b and 6 and Supplementary Note 4). This points to a previously underappreciated complexity of isoform expression for genes such as *Rufy3* in conferring specialization across neurodevelopment, cell (sub)types and brain regions.

Given regional differences, we systematically tested genes for altered isoform expression for matched cell types comparing one brain region to all other brain regions at P56 using our isoform tests and differential isoform quantification^32^ (Δ*Π*; Methods). First, we repeatedly downsampled highly expressed genes and tested for differential isoform expression between replicates of the same sample. Although downsampling reduces statistical power, it allows a fair comparison between replicates and/or brain regions. Between-region variability was consistently higher than between-replicate variability for most cell types (Fig. 2h). Second, 60–80% of commonly tested genes that were significant in replicate 1 were also significant in replicate 2 (Supplementary Fig. 7a). Leveraging the full dataset without downsampling, thalamic and cerebellar astroglia showed strong specialized isoform expression compared to other brain structures. This is particularly interesting because the cerebellum contains specialized Bergmann glia that are both morphologically and functionally distinct^40^. This high splicing specialization supports alternative splicing as an important influence on anatomical region morphology and function. At modest differences of 10% isoform usage between conditions (Δ*Π* = 0.1, false discovery rate ≤ 0.05; Methods), ~40% of tested genes showed a significant difference in isoform abundance between thalamic astrocytes and all other astrocytes. Neurons had more differentially expressed isoforms, with medium spiny neurons contributing to high brain region specificity of striatal inhibitory neurons and distinct hippocampal pyramidal cells contributing to region specificity in excitatory neurons. These differences were consistent, albeit to a lower extent, for increased Δ*Π* values across all cell types. However, for Δ*Π* ≥ 0.5, few brain region differences arose, indicating that regional differences in isoform expression arise from smaller modulations across many genes (Fig. 2i). Importantly, although the within-sample variability is a consideration in these results, the trends noticed are robust and highly cell-type specific. Using pseudobulk isoform expression between regions, the number of significant genes is considerably reduced (Supplementary Fig. 7b,c).

Although many genes exhibited unique regional signatures, most genes showed distinct isoform expression in two or three regions and rarely in four or five (Supplementary Fig. 7d,i). Compared to astrocytes, neurons exhibited higher levels of differential TSS and poly(A) site usage between regions, and neuronal TSS usage was more region specific (Fig. 2j). In summary, in addition to isoform regulation across development and between distinct cell (sub)types within an anatomical structure, the same cell type leverages distinct splicing patterns, TSSs and poly(A) sites in different regions.

### Marker exons delineate cell-type- and brain region-specific splicing patterns conserved in humans

To define precise transcript elements underlying splicing programs, we focused on individual exons. We considered exons alternatively included in at least one brain region or time point and calculated percent spliced in (*Ψ*) values for four main cell types (astrocytes, oligodendrocytes, excitatory neurons and inhibitory neurons) after ensuring reproducibility and averaging both replicates (Supplementary Fig. 8). An exon’s *Ψ* value can vary along the triad of (1) cell subtypes, (2) matched cell types at different ages or (3) brain regions (for example, P21:hippocampus:oligo, *n* = 44 triads; Supplementary Fig. 9). Pairwise correlations of triad *Ψ* values separated neuronal from nonneuronal populations, and all adult (P56) astrocytes clustered together regardless of region. However, hippocampal excitatory neurons clustered together regardless of age. Thus, unifying programs of age and/or brain region do not dictate splicing of distinct cell types (Supplementary Fig. 10).

**Fig. 3:**
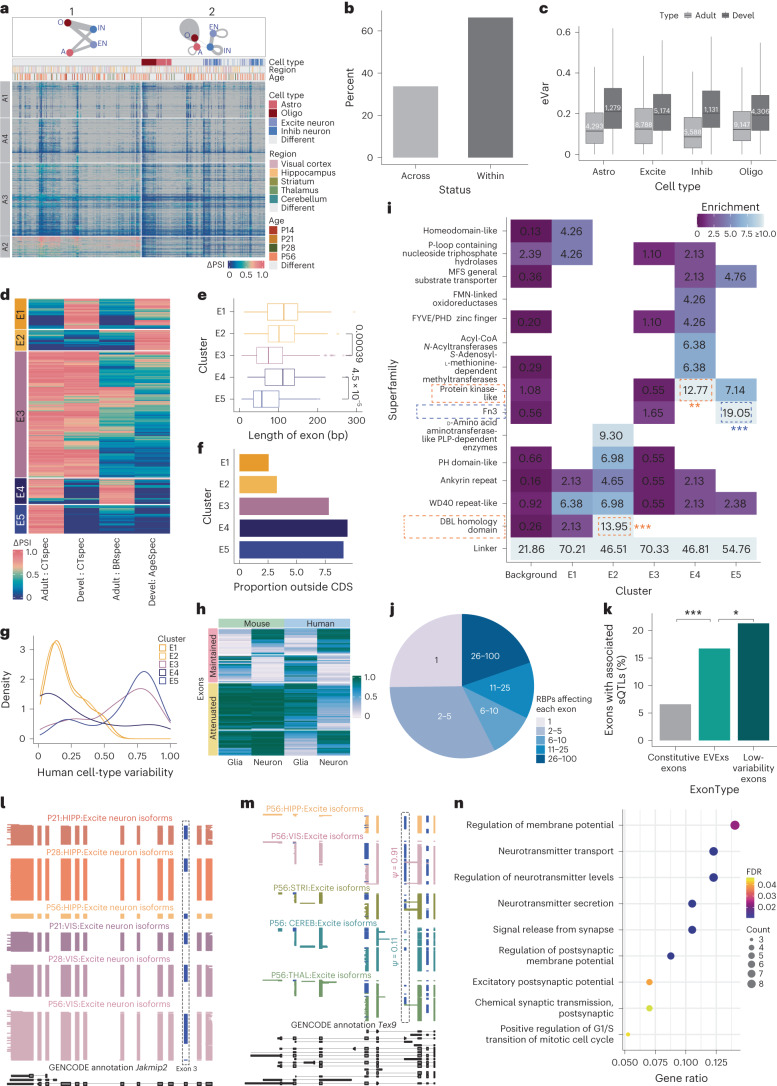
Marker exons underlying distinct splicing programs correlate with function and are conserved in humans. **a**, Δ*Ψ* heat map for pairwise cluster comparisons (columns) and exons (rows) where Δ*Ψ* ≥ 0.25 in at least one comparison; Δ*Ψ*, change in percent spliced index (PSI); O, oligodendrocytes; A, astrocytes; IN, inhibitory neurons; EN, excitatory neurons. **b**, Percentage of hVExs whose variability stems from a comparison of a matched cell type across brain regions or from two cell types in the same region. **c**, Maximal Δ*Ψ* values for matched cell types across brain regions and across developmental time points. The numbers of exons per condition are indicated in the plot; Devel, development. **d**, Heat map of EVExs (rows) and axes of variation (columns: adult cell-type specificity (CTspec), developmental cell-type specificity, adult brain region specificity (BRspec) and developmental (Age) specificity of a matched cell type). The five EVEx classes are indicated in the bar on the left. **e**, Length distribution of five EVEx classes. *P* values were obtained from a Wilcoxon’s two-sided test without correcting for multiple tests. **f**, Noncoding fraction for five EVEx classes; CDS, coding sequence. **g**, Cell-type variability of mouse EVExs in the human hippocampus. **h**, Heat map of neuron and glia *Ψ* values for mice (left) and humans (right) for exons that have high cell-type specificity in humans. The left annotation bar indicates whether cell-type specificity was maintained (pink) or attenuated (yellow) in mice. **i**, Protein domains enriched in five EVEx classes. **P < 0.01, ***P < 10^−^^5^. **j**, Pie chart of the number of RBPs significantly affecting each exon identified from human cell line data. **k**, Bar chart of the percentage of exons associated with a known brain region-specific sQTL for exons classified as constitutive, EVEx or lowly variable in mice. *P* values were obtained by using a Fisher’s two-sided exact test; *P < 0.05; ***P < 2.2 × 10^−16^. **l**, Cluster-resolved single-cell long reads for the *Jakmip2* gene. Each line is a single cDNA molecule. Blue exons indicate alternative exons. The top three tracks indicate hippocampal excitatory CA isoforms for P21, P28 and P56, and the next three tracks indicate the visual cortex excitatory isoforms from P21, P28 and P56. The bottom black track shows the GENCODE annotation. **m**, Similar as **l** for the *Tex9* gene with tracks colored for brain region of origin for P56 excitatory neurons. Data in **l** and **m** were plotted with ScisorWiz. **n**, GO biological process annotations for EVExs in E4 from **e**; FDR, false discovery rate. For box plots in **c** and **e**, the center lines indicate the median, box limits indicate the upper and lower quartiles, and whiskers indicate 1.5× the interquartile range.

Next, we compared exon inclusion between triads to isolate splicing programs. This yielded 4,557 exons with a 25% difference (Δ*Ψ* ≥ 0.25) in at least one comparison (highly variable exons (hVExs); Methods). The highest Δ*Ψ* values arose from neuron versus nonneuron comparisons (Fig. 3a, top left, bipartite network diagram). Moderate Δ*Ψ* values corresponded to comparisons between two neuronal or two glial triads (Fig. 3a, top right, fully connected network diagram). Many of the latter comparisons corresponded to differences between cell subtypes or to brain region and developmental differences of a matched cell type (see self-loops in the network). Clustering along rows defined four exon groups (Fig. 3a, A1–A4) whose genes harbor largely nonoverlapping functional ontologies. A1 and A4 hVExs have high Δ*Ψ* values for a few comparisons and low Δ*Ψ* values for most others. The genes of these exons are linked to regulatory roles for transcription, histone methylation and acetylation and synaptic signaling (Methods). Conversely, hVEXs exhibiting high Δ*Ψ* values in many comparisons, especially in the left half of the heat map (A2 and A3), belong to genes implicated in protein localization and neurotransmitter transport to the synapse. Together with the clean neuron-versus-glial split in the left half of the heat map, these observations emphasize the role of synaptic isoforms, rather than pure synaptic gene expression, in establishing neuronal and glial identities (Supplementary Fig. 11).

By definition, each hVEx arises from one or more triad comparisons with highly different *Ψ* values. Considering the triads contributing to hVExs, we found that, although most came from cluster comparisons within one brain region, a cell type could also show variability across brain regions (Supplementary Fig. 12). Indeed, 33.71% of these comparisons corresponded to a matched cell type between two brain regions, and 66.29% corresponded to a comparison within one brain region (Fig. 3b). Developmental variability exceeded variability between adult brain regions (both for matched cell types; median Δ*Ψ* = 0.197 and 0.115, respectively; Wilcoxon rank sum test *P* < 2.2 × 10^−16^). This was true for each major cell type, suggesting that cell types extensively modulate exon inclusion during development but reach homeostasis in adulthood for many genes (Fig. 3c).

To delineate markers of extensive splicing modulation, we defined extremely variable exons (EVExs; Δ*Ψ* ≥ 0.75 between two triads; Methods) as representing potential candidates for functionality. Specifically, we determined five exon groups (E1–E5), 89 exons in E1 with cell-type-specific inclusion during development but not in adulthood and 60 exons in E2 with temporal variability. The largest group, E3, had 373 exons with cell-type specificity in development and in adulthood. E4 included 75 exons with brain region variability between matched cell types, combined with cell-type specificity within a brain region. Thus, variability between matched cell types of different brain regions largely implied cell-type specialization within one region. Last, E5 contained 84 exons with cell-type specificity acquired only in adulthood (Fig. 3d). Exons in E3 and E5, that is, those with adult cell-type specificity regardless of earlier cell-type specificity, were markedly shorter, suggesting a link to microexons^29,41^. Exons in E1, E2 (transient cell-type specificity in development) and E4 (brain region-specific regulation) were all longer (Fig. 3e; two-sided Wilcoxon rank sum test *P* = 1.62 × 10^−10^). Moreover, exons with adult cell-type specificity (E3, E4 and E5) contained more noncoding sequence (Fig. 3f; Fisher’s exact test *P* = 1.5 × 10^−4^).

We produced single-cell long-read data for six adult human hippocampi and compared cell-type-specific exon inclusion between humans and mice (Methods and Supplementary Fig. 13). Among the EVExs, 24.09% (seen in E5, that is, adult cell-type signature) to 48.31% (in E1, that is, developmental cell-type- and time-specific signatures) of exon sequences and boundaries were conserved between species and were sufficiently expressed in our human hippocampal data (Methods and Supplementary Fig. 14a). Among those, exons with cell-type specificity in mice (E3 and E5) tended to also exhibit high cell-type specificity in humans (Fig. 3g, Wilcoxon rank sum test *P* < 2.2 × 10^−16^). Probing the reverse transferability from humans to mice revealed that among exons that were highly cell-type specific in the human hippocampus, ~42.6% showed similar cell-type specificity in the mouse hippocampus. However, ~57.4% of human cell-type-specific and almost 82.7% of invariable alternative exons in humans were constitutively included in mouse tissue, suggesting that human brains evolved some gain-of-function alternative exons, which cannot be modeled well in mice (Fig. 3h and Supplementary Fig. 14b).

Next, we estimated the functional impact of EVEx inclusion patterns by determining affected protein domains (Methods). Interdomain linkers typically represent intrinsically disordered regions, often mediating protein–protein interactions^42^, and were most frequent in all EVEx groups (*χ*^2^ test *P* < 0.05; Supplementary Fig. 15a). This suggests that EVExs affect protein function by rewiring signaling and regulatory networks in different cell types^43^. Additionally, protein repeats, including WD40 and ankyrin repeat (Fisher’s exact test *P* = 0.02) with short repetitive motifs, were frequently affected. Splicing is known to drive functional and structural diversity in these highly modular domains^44–46^. The protein kinase-like superfamily was highly enriched in exons displaying adult brain region-specific inclusion (E4), consistent with the known roles of kinases in synaptic plasticity^47^ (Fisher’s exact test *P* = 0.0054). Additionally, exons with transient developmental exon regulation (E2), were enriched for DBL homology domains as well as d-aminoacid aminotransferase-like PLP-dependent enzymes (Fisher’s exact test *P* = 8.29 × 10^−6^). These superfamilies are associated with cytoskeletal organization and neuronal development and morphogenesis^48^, processes governing both cell-type identity establishment and differentiation. Finally, adult cell-type-specific EVExs (E5), especially those distinguishing neurons from glia, are enriched for the fibronectin type III (Fn3) superfamily (Fisher’s exact test *P* = 3.2 × 10^−6^). NRCAM and NFASC, both of which have been associated with neural regulation^49–52^, exemplify such proteins. Their structure includes immunoglobulin-like domains, followed by several Fn3 domain repeats. The presence of Fn3 repeats in tandem with immunoglobulin domain repeats is not uncommon and is found in other proteins associated with the regulation of neuronal activity^53–55^ (Supplementary Fig. 15b,c). These findings indicate that biological programs defining EVEx inclusion are intrinsically tied to cellular identity and function (Fig. 3i; Wilcoxon rank sum test *P* < 2.2 × 10^−16^).

Based on preferential knockdown (KD) data of hundreds of RNA binding proteins (RBPs) in human cell lines^56,57^, we found 20% of human orthologs of EVExs to be affected by RBP KD. Many exons were significantly affected by multiple RBPs (Methods, Fig. 3j and Supplementary Fig. 16a,b). Indeed, inclusion of an alternative exon of the well-characterized *CLTA* gene was influenced by the expression of many RBPs (Supplementary Fig. 16c). Separating RBPs with a low (Δ*Ψ* < 0.4) versus high (Δ*Ψ* ≥ 0.4) effect on the *CLTA* exon from the human data, we correlated RBP expression with that of mouse cell types in adulthood. RBPs themselves followed a neuronal–glial split, and expression was highly correlated with exon inclusion in these cell types and especially so with the high-effect RBPs (Methods, Supplementary Note 5 and Supplementary Fig. 16d,e). Finally, visualizing the neuronal-versus-glial split in mouse cell types helped demonstrate the complex interplay of many RBPs modulating *Clta* exon inclusion in different brain regions (Supplementary Fig. 16f). This shows that the regulatory layer of cell-type-specific exon inclusion is somewhat conserved across species.

We additionally probed whether EVEx inclusion has known ramifications in human diseases. Sixteen percent of EVExs have an associated published splicing quantitative trait locus (sQTL),^58^ which largely arise from cell-type-specific EVEx categories (Methods, Fig. 3k and Supplementary Fig. 17a). Thus, we have identified a cell-type-specific component of sQTLs. Most genes containing these exons (81 of 114) have important known associations with neurological disorders, such as bipolar disorder, schizophrenia, Alzheimer’s disease, Parkinson’s disease, anxiety and depression (see Supplementary Note 6). This analysis opens the door to understanding the cell-type-specific effects of human variation on splicing in health and disease.

Some EVExs were unexpectedly alternatively spliced. Identified in E2 (that is, exons that change exon inclusion in early development), exon 3 of the *Jakmip2* gene, annotated as constitutive, is developmentally regulated in hippocampal, but not visual cortex, excitatory neurons (Fig. 3l). Similarly, the testis-expressed 9 (*Tex9*) gene exhibits low brain region specificity in protein and single-cell RNA data^59,60^. However, *Tex9* exon 5 belongs to exon group E4, which represents exon variability across brain regions and cell types in adulthood, and the exon indeed marks brain regions. Visual cortex excitatory neurons have near-constitutive inclusion, whereas the excitatory neurons of other brain regions show inclusion as low as 11% (Fig. 3m). Gene ontology (GO) enrichment of neurotransmitter secretion and synaptic potential in E4 indicates that matched cell types expressing the same gene across brain regions modulate neuronal function using alternative splicing (Fig. 3n). These findings further underscore the role of alternative exons in conferring developmental, cell-type and brain region specificity.

### Adolescence transiently increases brain region specificity

**Fig. 4:**
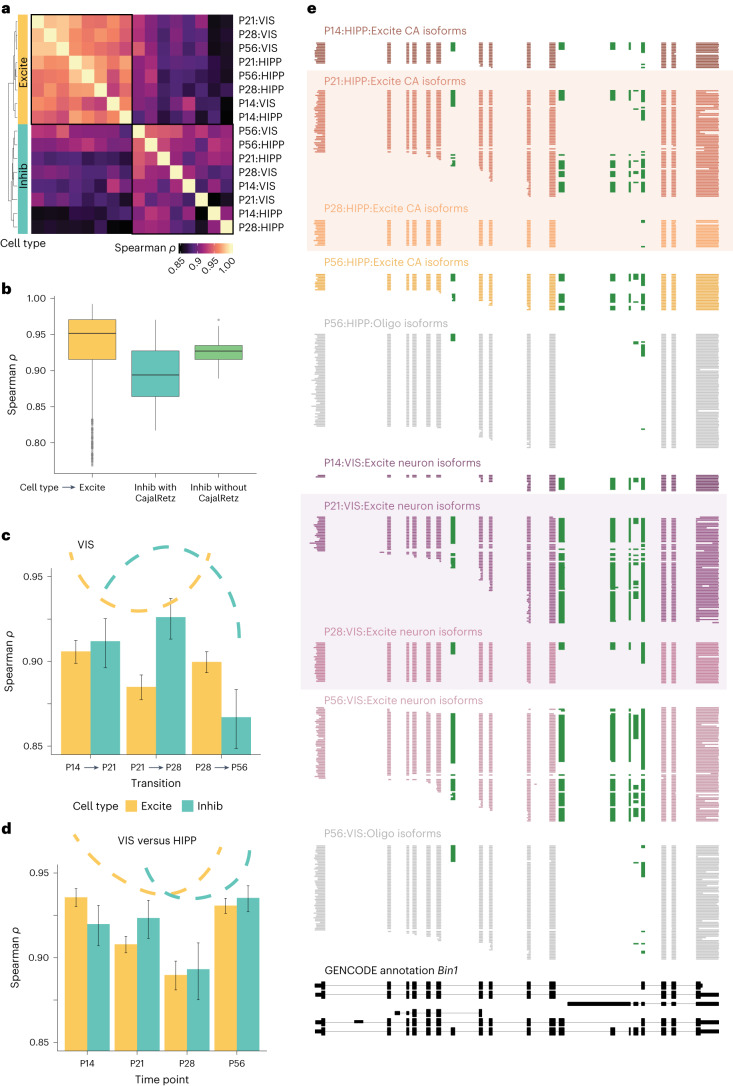
Neuronal exon inclusion changes between the mouse visual cortex and hippocampus and over time. **a**, Heat map of pairwise correlations of exon inclusion (*Ψ*) for excitatory and inhibitory types. **b**, Box plot of pairwise correlations of *Ψ* values for pairs of excitatory subtypes (*n* = 30), all inhibitory subtypes (*n* = 12) and inhibitory subtypes excluding Cajal–Retzius cells (*n* = 8). The center lines indicate the median, box limits indicate the upper and lower quartiles, and whiskers indicate 1.5× the interquartile range. **c**, Correlations of exon inclusion for excitatory clusters between neighboring time points in the visual cortex (yellow). The same is presented for inhibitory clusters (green). **d**, Correlations of exon inclusion for excitatory clusters at each time point between the visual cortex and hippocampus (yellow). The same is presented for inhibitory clusters (green). In **c** and **d**, error bars indicate 95% confidence intervals around the Spearman’s correlation. **e**, Depiction of cell-type-resolved single-cell long reads for the *Bin1* gene in excitatory neurons in the hippocampus and visual cortex. Each line represents one individual cDNA molecule, and blocks are colored by cell type and time point. Green represents alternative exons. Gray blocks indicate oligodendrocyte populations at P56. The bottom black track shows the GENCODE annotation.

Given that age alone was not enough to define isoform differences (refer to Supplementary Fig. 8), we delineated cell-type and brain region specificity in development. We first correlated neuronal cell subtype *Ψ* values from hippocampus and visual cortex developmental time points. Hierarchical clustering separated excitatory from inhibitory populations independently of brain region and developmental stage (Fig. 4a). Surprisingly, pairwise correlations were higher between excitatory types than between inhibitory neuron types (Supplementary Fig. 18; two-sided Wilcoxon rank sum test *P* = 5.099 × 10^−14^). We then considered finer excitatory cell subtypes. Clustering correlation values of exon inclusion revealed four main groups, corresponding to (1) neuronal intermediate progenitor cells, (2) granule neuroblasts, (3) mature neurons including excitatory and inhibitory subtypes and (4) multiple Cajal–Retzius clusters, with a unique cell-type signature (Supplementary Fig. 19). Removing Cajal–Retzius subtypes from inhibitory neurons increased intrainhibitory neuron correlation, but this correlation remained lower than pairwise excitatory cluster correlations. Thus, Cajal–Retzius cells contribute strongly to hippocampal inhibitory neuron diversity but do not fully account for it (Fig. 4b). We examined if time points with large developmental shifts distinguished brain regions. In the visual cortex, excitatory neuron correlations between adjacent time points were lowest at the P21-to-P28 transition, with higher values before and after, whereas inhibitory neurons showed the opposite (Fig. 4c). After correlating *Ψ* values between the visual cortex and hippocampus for matched cell types, excitatory neurons showed high correlation at P14 and P56 but lower correlation at P21 and especially P28. Thus, developmental timelines of excitatory neuron splicing differ between the visual cortex and hippocampus, and brain region specificity transiently increases. A similar, albeit weaker, observation was made for inhibitory neurons. The lowest hippocampus-versus-visual cortex correlation occurred at the P21-to-P28 critical developmental period^61,62^, indicating nonaligned splicing shifts for cortical and hippocampal excitatory and inhibitory neurons (Fig. 4d). *Bin1*, a synaptic gene implicated in Alzheimer’s disease, exemplifies regional specificity in excitatory neurons and temporal regulation at the P21–P28 transition. Hippocampal excitatory CA neurons express the P1 cerebellar neuronal isoform^61^ at P14 and as the main isoform at P56, which begins to transiently disappear at P21 and is almost entirely absent at P28. At P28, *Bin1* excitatory neuron isoforms resemble the isoform profile of oligodendrocytes, skipping all six alternative exons. Although this is also observed in the visual cortex, the transition is drastic from P21 to P28, marking a brain region difference between the hippocampus and visual cortex (Fig. 4e). Similar patterns arise in other disease-relevant genes, including *Mapt* (Supplementary Fig. 20a,b). In summary, cell-type and brain region specificity in isoform expression can be transient for some genes, blurring the lines of cell-type-specific splicing. This temporal difference is an important consideration especially during development.

### Discordance in splicing and expression-defined glial maturation

**Fig. 5:**
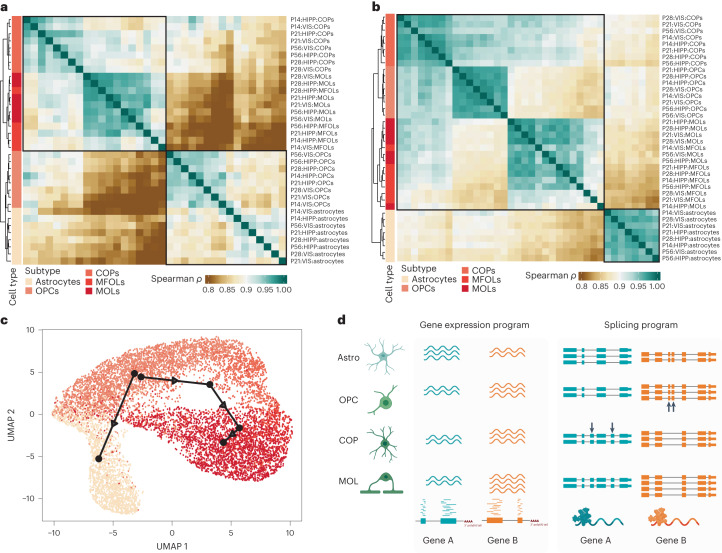
Exon inclusion patterns in glial subtypes suggest an ordered molecular cascade. **a**, Heat map based on pairwise correlations of exon inclusion patterns for astrocyte and oligodendrocyte lineage cells. **b**, Similar heat map based on pairwise gene expression values. **c**, Slingshot trajectory of glial cells using exon inclusion values. **d**, Model depiction summarizing the findings of the presented data. Subtypes in the oligodendrocyte lineage have similar gene expression patterns. However, a switch in splicing patterns occurs after OPCs have matured to committed oligodendrocyte precursors. Arrows represent alternative exons.

Similar to our temporal neuronal analysis, we determined exon inclusion levels for 35 astrocyte clusters and 1 oligodendrocyte cluster. Surprisingly, clustering of correlations revealed that the first split in the dendrogram separated astrocytes and all oligodendrocyte precursor cell (OPC) clusters, regardless of age, from all committed and mature oligodendrocytes (Fig. 5a). In stark contrast, a similar gene expression analysis grouped all oligodendrocyte lineage clusters together, well separated from astrocytes (Fig. 5b). Consistently, a pseudotime trajectory^63^ using single-cell isoform data (Methods) with a starting point defined at OPCs revealed two trajectories, one toward astrocytes and one along the oligodendrocyte lineage. Of note, the trajectory from OPCs to astrocytes likely does not represent a maturation pattern but rather the fact that, mathematically, OPC splicing is close to astrocytic splicing (Fig. 5c). Taken together, these analyses support divergent cell group similarities observable in splicing and 3′ gene expression patterns motivating cell-type identity partially defined by splicing (Fig. 5d).

### Fluctuating patterns of exon variability in development

**Fig. 6:**
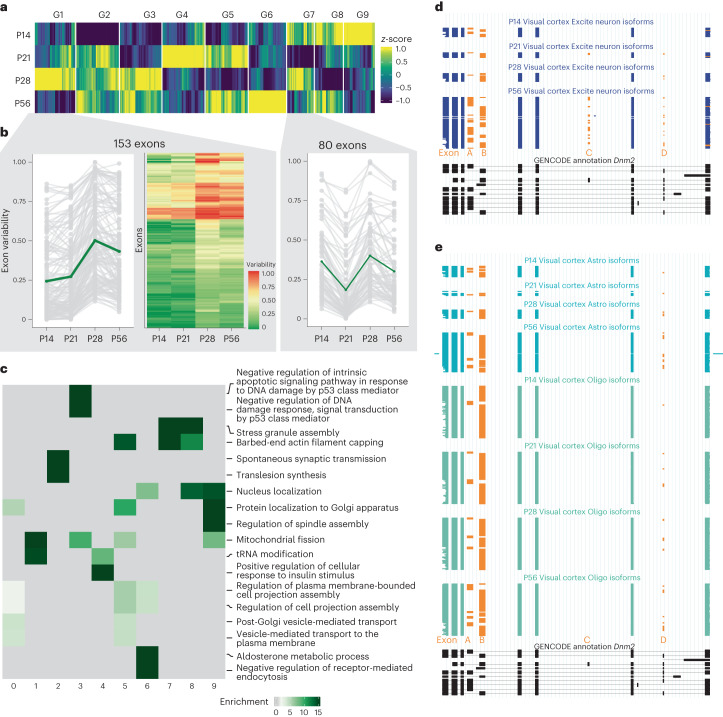
Developmental exon regulation reveals convergent and divergent patterns of exon variability. **a**, Heat map of *z*-normalized exon variability between the four major cell types across development. **b**, Line plot of raw values of exon variability for individual genes in groups 3 (left) and 7 (right) for the visual cortex and heat map of exon variability for group 7 in the visual cortex (middle). Some show lower changes, whereas others exhibit drastic differences. **c**, Heat map of GO enrichment values for highly enriched sets of genes contributing to the nine groups from **a**. **d**, Depiction of time point-resolved single-cell long reads from visual cortex excitatory neurons for the *Dnm2* gene. Each line represents one individual cDNA molecule. Alternative exons are denoted in orange and are marked A through D. **e**, Same as in **d** but for astrocyte (teal) and oligodendrocyte (sea green) clusters. The bottom black track shows the GENCODE annotation.

We hypothesized that EVExs with temporal regulation (E1–E2; Fig. 3d) were related to the correlation drop around P28 (Fig. 4c,d). We focused on 1,072 exons that substantially change exon variability between time points (Supplementary Fig. 21 and Methods). Three transitions, each of (1) increasing, (2) decreasing or (3) constant variability, define 3^3^ = 27 patterns, but after removing invariable exons (G0), only 9 were frequent (denoted G1–G9; Fig. 6a). For genes containing multiple alternative exons, 36% had all exons exhibiting fluctuations in a single pattern (*n* = 106 hippocampus, *n* = 115 visual cortex), whereas 64% had exons in two or more patterns (*n* = 195 hippocampus, *n* = 204 visual cortex). Interestingly, pairs of neighboring exons were frequently included or skipped together (Methods and Supplementary Fig. 22a). The P21-to-P28 transition consistently exhibited drastic shifts in exon variability (Fig. 6b and Supplementary Figs. 23 and 24, Bernoulli *P* *=* 1.89 × 10^–6^). Exon inclusion variability between cell types had the highest standard deviation at P28 in both the hippocampus and visual cortex, and we found that pairs of exons were tightly coordinated at this time point (Supplementary Fig. 22b). These observations further suggest that the difference between cell types can transiently change during this critical period (Supplementary Fig. 25).

Genes unique to each of the G1–G9 patterns were largely nonoverlapping in function (Supplementary Fig. 26). Of note, synaptic vesicle budding and transport was associated with genes exhibiting an exon variability increase at P28 (G1), whereas actin filament capping and functions associated with cell projection were associated with G5 with an increase in exon variability at P21 and P56. Both observations support a developmentally regulated neuronal-versus-glial split (Fig. 6c and Supplementary Fig. 26). The dynamin 2 (*Dnm2*) gene is ubiquitously expressed, has known roles in intracellular membrane trafficking and cytoskeleton organization and is associated with neurological diseases^64^. In the visual cortex, *Dnm2* has exons in G5 and G8 and therefore exemplifies repetitive developmental changes in cell-type variability (Fig. 6d,e and Supplementary Note 7). *Dnm2* not only varies across the cell-type and developmental axes but also shows differing patterns of isoform regulation between the visual cortex and hippocampus (compare Fig. 6d,e to Supplementary Fig. 27). This example highlights how cell types leverage inclusion patterns of multiple exons in tandem for developmental and brain region-specific specialization.

## Discussion

A large body of research has established that isoform expression underlies physiological differences in brain regions and cell types and is altered in development and evolution. However, a unified view of these dimensions remained elusive.

We found that cell types vary extensively in their splicing patterns across these axes. Although exons most frequently modulate their inclusion between cell types within a brain region, we find compelling evidence for differences between matched cell types across regions, involving neurotransmitter secretion and regulation as well as synaptic regulation. Thus, cell types that are considered homogenous and are ubiquitously present in brain tissue display specialized splicing patterns depending on their region of origin. This is particularly true for astrocytes in the thalamus and the cerebellum, which differ not only in their exon inclusion but also in their TSS and poly(A) site usage, rendering the mRNA isoform landscape more complex than previously imagined.

We defined EVExs across brain regions, development and cell types and in combinations thereof. These exon groups exhibit key differences in properties such as length, protein coding capacity and protein domain architecture. Therefore, distinct programs of exon variability correlate with functional consequences of proteins. Remaining questions include the precise definition of regulatory elements governing inclusion variability and its conservation in humans. Nonetheless, we found that adult cell-type variability is largely recapitulated in human single-nucleus long-read data. Extrapolating from human data, the KD of multiple RBPs in tandem influences exon inclusion patterns, and sQTLs influence exon inclusion of a significant number of alternative exons, particularly of cell-type-specific exons. Therefore, many of these cell-type-specific mouse results can be extended to human brain and disease conditions.

Importantly, the same cell type traced along development exhibits more isoform fluctuation than across adult brain regions. Synapse formation, axon guidance and general neural network formation induce a temporal splicing heterogeneity within cell populations that is attenuated in adulthood. This observation is further strengthened by nine patterns of developmental variability that we identify, a majority of which involve fluctuations during the critical developmental period^61,62^ of mouse adolescence (P21–P28) in the visual cortex and hippocampus. We repeatedly find that exons that are cell-type specific at a developmental time point can transiently change inclusion status and lose their cell-type specificity. Fundamental genes, such as *Bin1* and *Mapt*, show such transient cell-type specificity in isoform expression, suggesting highly sophisticated developmental splicing programs. Additionally, our data reveal the precise timing of splicing switches in maturation programs, especially on the oligodendrocyte lineage after the split from astrocytes. This adds subtlety to studies of isoform expression over development and justifies the need for simultaneous recording of gene expression and splicing.

Taken together, we present a comprehensive single-cell investigation of alternative isoform usage in the brains of mice and humans and the dynamics of isoform expression variation across anatomical structures and development.

## Methods

### Ethics statement

All experiments were conducted in accordance with relevant National Institutes of Health (NIH) guidelines and regulations related to the Care and Use of Laboratory Animals. All animal procedures were approved by the Institutional Animal Care and Use Committee of Weill Cornell Medicine and were in accordance with the 2011 Eighth Edition of the NIH Guide for the Care and Use of Laboratory Animals. Human tissue samples were acquired through the NIH NeuroBioBank and were compliant with research ethics stated by the NIH. All donors completed University of Maryland Institutional Review Board-approved consent documents. They were informed via these consent documents that the donated tissue would be used for distribution to qualified researchers and that such distributions could be made at any time in the future. These consent documents also assured that the identity of the donor would remain unknown to any tissue recipients and those reviewing the results of their work.

### Experimental design and analysis considerations

The null hypothesis of the study was that brain regions and developmental time points of wild-type and healthy animals had no impact on alternative splicing patterns. No experimental manipulations of mice were performed. The study design was hence observational (known samples collected at different time points) and did not require randomization of experimental or control groups. Additionally, data collection and analysis were not performed blind to the conditions of the experiments. No animals were excluded from the analysis; however, in cases where adequate data points were not available (for example, minimum number of reads per gene), then those points were excluded. No statistical methods were used to predetermine sample sizes (for example, cell number in single-cell experiments), but our sample sizes are similar to those reported in previous publications^65,66^. Statistical tests used in this manuscript largely involve Fisher’s exact test and Wilcoxon rank sum test, which do not make assumptions about the underlying data distribution. Some analyses used *χ*^2^ tests, for which the *χ*^2^ criterion was checked before testing^32^.

### ScISOr-Seq2: single-cell isoform RNA sequencing from adolescent and adult mouse brain

#### Tissue acquisition

C57BL/6NTac mice (male, P14: *N* = 2, P21: *N* = 2, P28: *N* = 2, P56: *N* = 6; see Supplementary Table 1) were housed in groups of three to four per cage with a 12 h-light/12-h-dark cycle and ad libitum access to food and water. Ambient temperature and humidity were centrally regulated. Mice were perfused with 25 ml of ice-cold and carbogen-treated 1× partial sucrose cutting solution containing 5 µg ml^–1^ actinomycin D. The remaining 1× partial sucrose cutting solution and Earle’s balanced salt solution were oxygenated. Dissection of specific brain regions was conducted by using the mouse three-dimensional coronal sections from Allen Brain Atlas as a reference map for coordinates. The brain slices were collected on a vibratome (Leica) at a thickness of 300 µm per slice and kept in ice-cold 1× partial sucrose cutting solution. For the hippocampus, eight to ten mouse coronal slices (300 µm) were collected from the caudal region of the brain after removing the cerebellum. The hippocampus region was dissected out based on the mouse coronal sections (images 62~89). Note, each image section on Allen Brain Atlas is spaced at 100-µm intervals. Eight to ten slices can cover almost the whole hippocampus region. The visual cortex was collected based on images 79–100. The first one or two slices from the caudal region of the brain were discarded, and subsequently five to six continuous slices were collected. For the striatum, five to six mouse coronal slices were collected from the rostral region of the brain after removing the olfactory bulb. The striatum was dissected out based on the mouse coronal sections (images 39~59). Dissection of the cerebellum does not require any vibratome sectioning. The cerebellum was dissected based on the location and structure with forceps and minced into small pieces with a scalpel. Slices were transferred to a slicing chamber with bubbling 1× partial sucrose containing small-molecule mix B at room temperature, and slices were allowed to recover for ~30 min. The 1× cutting solution contained the following components: 93 mM *N*-methyl-d-glucamine (Acros Organics, AC126841000), 2.5 mM KCl (Sigma, 44675), 1.2 mM NaH_2_PO_4_ (Sigma, S5011), 30 mM NaHCO_3_ (Sigma, S5761), 20 mM HEPES (Gibco, 15630106), 25 mM glucose (Sigma, G7021), 5 mM sodium ascorbate (Sigma, A4034), 2 mM thiourea (Alfa Aesar, AAA1282822), 3 mM sodium pyruvate (Gibco, 11360070), 10 mM *N*-acetyl-l-cysteine (Alfa Aesar, AAA1540914), 0.5 mM CaCl_2_ (Sigma, 223506) and 10 mM MgSO_4_ (Sigma, M2643), pH 7.2–7.4.

#### Single-cell disassociation

Tissue sections were dissociated by using a previous protocol with modification from the Smit lab at Vrije Universiteit, the Netherlands. Regions of interest were dissected on a Sylgard-coated plate with a dark background in 1–2 ml of carbogen-treated cutting solution. Tissue pieces were transferred to 5 ml of 2 mg ml^–1^ activated papain (Worthington, LK003150) and incubated for 15–25 min at 37 °C with gentle mixing. After the incubation, the tissue was cut into tiny pieces and gently triturated 15–20 times using large- to small-sized Pasteur pipettes until no obvious chunks were observed. Pasteur pipettes with different opening sizes (large: 0.6–0.7 cm, medium: 0.3–0.4 cm, small: 0.15–0.2 cm) were created by flame polishing disposable glass Pasteur pipettes (Thermo Fisher) and assembled with rubber bulbs. After undissociated tissue chunks settled down, the supernatant was taken and filtered using a 30-μm cell strainer (Miltenyi Biotec, 130-041-407) into a nuclease-free collection tube. The supernatant was then centrifuged at 300–400*g* for 5 min at room temperature. After discarding the supernatant, the cell pellet was resuspended in 3 ml of 10% ovomucoid protease inhibitor solution (150 µl of DNase I, 300 µl of ovomucoid protease inhibitor solution and 2.55 ml of Earle’s balanced salt solution; Worthington, LK003150). Next, the cell suspension was slowly and gently added to the top layer of 5 ml of ovomucoid protease inhibitor solution (Worthington, LK003150) without interfering with the bottom layer. The cells were spun down by centrifugation at 70–100*g* for 6 min at room temperature. After removing all the supernatant, cells were suspended in 1 ml of fluorescence-activated cell sorting (FACS) buffer (1× HBSS (Gibco, 14175079) containing 0.2% bovine serum albumin (Thermo Scientific, 37525), 25 mM glucose (Sigma, G7021), 3 mM sodium pyruvate (Gibco, 11360070) and 0.2 U µl^–1^ RNase inhibitor (Ambion, AM2682)). After incubation for 15 min in FACS buffer with 0.1 µg ml^–1^ DAPI (Sigma, D9542), viable cells were collected as a DAPI-negative population using a Sony MA900 sorter with FlowJo version 10 software. Sorted viable cells were centrifuged and subsequently diluted to 1,000–1,500 cells per μl in FACS buffer for capture on the 10x Genomics Chromium controller.

### SnISOr-Seq: single-nucleus isoform sequencing in frozen human tissue

#### Sample acquisition

Six healthy human brain samples (hippocampus, three male and three female; Supplementary Table 5) used for this study were requested through the NIH NeuroBioBank and were obtained from the University of Maryland Brain and Tissue Bank according to Institutional Review Board-approved protocols. No donors had pre-existing neurodegenerative or neurological diseases. Tissues were flash-frozen and maintained at −80 °C until processing.

#### Single-nucleus isolation

Approximately 30 mg of frozen tissue from each sample was dissected in a sterile dish on dry ice and transferred to a 2-ml glass tube containing 1.5 ml of nuclei pure lysis buffer (MilliporeSigma, L9286) on ice. Tissue was completely minced and homogenized to a nuclei suspension by grinding samples with with Dounce homogenizers (MilliporeSigma, D8938-1SET) with 20 strokes with pestle A and 18 strokes with pestle B. The nuclei suspension was filtered by loading through a 35-µm-diameter filter, followed by centrifuging for 5 min at 600*g* and 4 °C. The nuclei pellet was collected and washed with cold wash buffer (1× PBS (Corning, 46-013-CM), 20 mM DTT (Thermo Fisher Scientific, P2325), 1% bovine serum albumin (New England Biolabs, B9000S) and 0.2 U µl^–1^ RNase inhibitor (Ambion, AM2682)) three times. After removing the supernatant from the last wash, the nuclei were resuspended in 1 ml of 0.5 µg ml^–1^ DAPI (MilliporeSigma, D9542) containing wash buffer to stain for 15 min. The nuclei suspension was prepared for sorting by filtering cell aggregates and particles out with a diameter of 35 µm. After removing myelin and fractured nuclei by sorting, the nuclei were collected by centrifuging for 5 min at 600*g* and 4 °C and resuspended in wash buffer to reach a final concentration of 1 × 10^6^ nuclei per ml after counting in trypan blue (Thermo Fisher Scientific, T10282) using a Countess II cell counter (Thermo Fisher Scientific, A27977).

### Linear/asymmetric PCR (LAP) to remove nonbarcoded cDNA

The first-round PCR protocol (95 °C for 3 min, 12 cycles of 98 °C for 20 s, 64 °C for 30 s and 72 °C for 60 s) was performed by applying 12 cycles of linear/asymmetric amplification to preferentially amplify one strand of the cDNA template (30 ng of cDNA generated by using a 10x Genomics Chromium Single Cell 3′ GEM kit) with the primer ‘Partial Read1’, and the product was purified with 0.8× SPRIselect beads (Beckman Coulter, B23318) and washed twice with 80% ethanol. The second-round PCR was performed by applying six cycles of exponential amplification under the same conditions with forward primer ‘Partial Read1’ and reverse primer ‘Partial TSO’, and the product was purified with 0.6× SPRIselect beads, washed twice with 80% ethanol and eluted in 30 µl of buffer EB (Qiagen, 19086). The following primer sequences were used: 5′-CTACACGACGCTCTTCCGATCT-3′ (Partial Read1) and 5′-AAGCAGTGGTATCAACGCAGAGTACAT-3′ (Partial TSO). KAPA HiFi HotStart PCR Ready Mix (2×; Roche, KK2601) was used as the polymerase for all the PCR amplification steps in this paper, except for the 10x Genomics 3′ library construction.

### Exome capture (CAP) to enrich for spliced cDNA

Exome enrichment was applied to the cDNA purified from the previous step by using a SSELXT Human All Exon V8 probe kit (Agilent, 5191-6879) for human samples or SureSelectXT Mouse All Exon (Agilent, 5190-4641) for mouse samples. The reagent kit SureSelectXT HSQ (Agilent, G9611A) was used according to the manufacturer’s manual. First, the block oligonucleotide mix was made by mixing an equal amount (1 µl of each per reaction) of Partial Read1 primer (5′-CTACACGACGCTCTTCCGATCT-3′) and Partial TSO primer (5′-AAGCAGTGGTATCAACGCAGAGTACAT-3′) with a concentration of 200 ng µl^–1^ (Integrated DNA Technologies), resulting in 100 ng µl^−1^. Next, 5 µl of 100 ng µl^–1^ cDNA diluted from the previous step was combined with 2 µl of block mix and 2 µl of nuclease-free water (New England Biolabs, AM9937), and the cDNA block oligonucleotide mix was incubated on a thermocycler under the following conditions to allow the block oligonucleotide mix to bind to the 5′ end and the 3′ end of the cDNA molecule: 95 °C for 5 min, 65 °C for 5 min and 65 °C on hold. For the next step, the hybridization mix was prepared by combining 20 ml of SureSelect Hyb1, 0.8 ml of SureSelect Hyb2, 8.0 ml of SureSelect Hyb3 and 10.4 ml of SureSelect Hyb4 and kept at room temperature. Once the reaction reached 65 °C on hold, 5 µl of probe, 1.5 µl of nuclease-free water, 0.5 µl of 1:4 diluted RNase Block and 13 µl of the hybridization mix were added to the cDNA block oligonucleotide mix and incubated for 24 h at 65 °C. When the incubation reached the end, the hybridization reaction was transferred to room temperature. Simultaneously, an aliquot of 75 µl of M-270 Streptavidin Dynabeads (Thermo Fisher Scientific, 65305) was prepared by washing three times and was resuspended with 200 µl of binding buffer. Next, the hybridization reaction was mixed with all the M-270 Dynabeads and placed on a Hula mixer for 30 min at room temperature. During the incubation, 600 µl of wash buffer 2 (WB2) was transferred to three wells of a 0.2-ml PCR tube and incubated in a thermocycler on hold at 65 °C. After the 30-min incubation, the buffer was replaced with 200 µl of WB1. The tube containing the hybridization product bound to M-270 Dynabeads was put back into the Hula mixer for another 15-min incubation at low speed. Next, WB1 was replaced with WB2, and the tube was transferred to the thermocycler for the next round of incubation. Overall, the hybridization product bound to M-270 Dynabeads was incubated in WB2 for 30 min at 65 °C, and the buffer was replaced with fresh preheated WB2 every 10 min. When the incubation was over, WB2 was removed, and the beads were resuspended in 18 µl of nuclease-free water and stored at 4 °C. Next, the spliced cDNA, which bound with the M-270 Dynabeads, was amplified with primers Partial Read1 and Partial TSO by using the following PCR protocol: 95 °C for 3 min, 12 cycles of 98 °C for 20 s, 64 °C for 60 s and 72 °C for 3 min. The amplified spliced cDNA was isolated from M-270 beads as supernatant, followed by a purification with 0.8× SPRIselect beads.

### 10x 3′ library preparation and sequencing

A single-cell/single-nucleus suspension containing 10,000 cells/10,000 nuclei was loaded on a Chromium Single Cell B Chip (10x Genomics, 1000154). Specifically, 75 µl of master mix + nuclei suspension was loaded into the row labeled 1, 40 µl of Chromium Single Cell 3′ Gel Beads (10x Genomics, PN-1000093) was loaded into the row labeled 2, and 280 µl of partitioning oil (10x Genomics, 2000190) was loaded into the row labeled 3. This was followed by GEM generation and barcoding after GEM-RT cleanup and cDNA amplification. Then, 100 ng of purified cDNA derived from 12 cycles of cDNA amplification was used for 3′ Gene Expression Library Construction by using Chromium Single Cell 3′ GEM, Library & Gel Bead kit v3 (10x Genomics, 1000092) according to the manufacturer’s manual (10x Genomics, CG000183 Rev C). The barcoded short-read libraries were measured using a Qubit 2.0 with a Qubit dsDNA HS assay kit (Invitrogen, Q32854), and library quality was assessed on a Fragment analyzer (Agilent) using a high-sensitivity NGS Fragment kit (1–6,000 base pairs (bp); Agilent, DNF-474-0500). Sequencing libraries were loaded on an Illumina NovaSeq6000 with PE 2 × 50 paired-end kits by using the following read lengths: 28 cycles read 1, 8 cycles i7 index and 91 cycles read 2.

### Library preparation for PacBio

HiFi SMRTbell libraries were constructed according to the manufacturer’s manual by using a SMRTbell Express Template Prep kit 2.0 (PacBio, 100-938-900). For all samples, ~500 ng of cDNA obtained by performing linear/asymmetric PCR followed by exome capture (LAP-CAP) from the previous step was used for library preparation. The library construction includes DNA damage repair (37 °C for 30 min), end repair/A tailing (20 °C for 30 min and 65 °C for 30 min), adaptor ligation (20 °C for 60 min) and purification with 0.6× SPRIselect beads.

### Library preparation for ONT

For all samples, ~75 fmol of cDNA was processed with LAP-CAP and underwent ONT library construction by using a Ligation Sequencing kit (ONT, SQK-LSK110), according to the manufacturer’s protocol (Nanopore Protocol, Amplicons by Ligation, version ACDE_9110_v110_revC_10Nov2020). The ONT library was loaded onto a PromethION sequencer by using a PromethION Flow Cell (ONT, FLO-PRO002) and was sequenced for 72 h. Base calling was performed with Guppy by setting the base quality score to >7.

### Short-read assignment of cell types (mouse)

Fastq files were obtained from the Illumina sequencing reads by running bcl2fastq. Gene × cell matrices processed with CellRanger V3.1.0 were loaded into Seurat V3.2.3 (ref. ^36^) and preprocessed individually using cutoffs described in Supplementary Table 4. After filtering for high-quality cells, they were scaled and normalized using default parameters and clustered using the Louvain algorithm. Doublet clusters were discarded. Subsequently, all samples from the hippocampal developmental lineage were processed together, as were the samples from the visual cortex lineage. After combining the data without any integration approaches and using the Seurat merge function, the data were scaled and normalized, and variable genes were identified. Integration of the data to control for sample-specific batch effects was performed using Harmony. Cell types were assigned using marker genes in the following three levels of granularity: broad, cell type and cell subtype. These were then assigned to each single cell along with the information on replicate, brain region and age. For the spatial axis, that is, the cerebellum, striatum and thalamus, the two replicates were integrated with Harmony^38^ before assigning cell types in the same three levels of granularity as described above. Finally, the entire dataset was merged together into a single object for visualization purposes and to obtain summary statistics in Fig. 1. This was also done using Harmony while controlling for region-specific differences in gene expression.

### Short-read assignment of cell types (human)

Fastq files were obtained from the Illumina sequencing reads by running bcl2fastq. Gene × cell matrices processed with CellRanger v3.1.0 were loaded into Seurat 3.2.2 and preprocessed individually. After filtering for high-quality cells, they were scaled and normalized using default parameters and clustered using the Louvain algorithm. Doublet clusters were discarded. Subsequently, all samples were processed together. After combining the data without any integration approaches and using the Seurat merge function, the data were scaled and normalized, and variable genes were identified. Integration of data to control for sample-specific batch effects was performed using Harmony^38^. Cell types were assigned using marker genes in the following three levels of granularity: broad, cell type and cell subtype. These were then assigned to each single cell along with the information on sample ID.

### Generation of PacBio circular consensus reads

Using the default SMRT-Link (v8.0.0.78867) parameters, we performed circular consensus sequencing (8.0.0.80529) with IsoSeq3 with the following modified parameters: maximum subread length of 14,000 bp, minimum subread length of 10 bp and minimum number of passes of 3.

### Preprocessing of long-read sequencing data with PacBio

Subread fastq files were obtained from circular consensus sequencing performed using the parameters described above. Data were processed using the scisorseqr^32^ pipeline by first aligning to the genome using STARlong (v2.7.0). Cellular barcodes were assigned using the cell-type and sample information from the short-read analysis as input to the GetBarcodes() function. Subsequently, uniquely mapped, spliced barcoded reads were obtained using the MapAndFilter() and InfoPerLongRead() functions in scisorseqr.

### Preprocessing of long-read sequencing data with ONT

Reads were basecalled using MinKNOW Core (v4.0.5), Bream (v6.0.10) and guppy (v4.0.11) on the PromethION machine. Reads were aligned using minimap2 (ref. ^67^; v2.17-r943-dirty), and data were preprocessed using the scisorseqr (v0.1.9)^32^ package. Cell barcodes were assigned using the cell-type and sample information from the short-read analysis.

### PacBio transcript assignment using IsoQuant

IsoQuant (v2.3.0) was run using default PacBio parameters on an aggregate of all barcoded PacBio reads with GENCODE v21 as annotation. Multiexonic transcripts classified as ‘novel in catalog’ were then used to create an enhanced annotation

### ONT transcript assignment using IsoQuant

This enhanced annotation gtf file was used on each of the ONT samples to correct incorrectly assigned splice sites in multiexonic barcoded reads. Isoquant (v3.1) was run using default parameters for ONT data. These corrected splice sites were then reassigned to reads in the AllInfo file, which was then filtered for unique molecular identifiers and used as input in subsequent analyses. More information is available in Supplementary Note 3.

### Obtaining full-length isoform variability across three axes

To find regional variability, we first calculated the percent inclusion (*Π*) for each isoform in a region by summing the inclusion values over all cell subtypes and time points for that region. A *Π* value is only calculated if the minimum number of reads in a gene for that region is at least 10. If at least two regions have *Π* values, we obtain an isoform × region matrix of *Π* values. We then define the variability per isoform as the max(*Π*) – min(*Π*). The brain region contributing the most to the region-specific variability is recorded as having the *Π* with the highest divergence from the median *Π* value.

The same procedure was performed to calculate age and subtype variability of a given cell type provided that the minimum read number requirements per age and subtype in a gene were met. More formally, for a cell type (CT), all possible clusters can be represented as a combination of brain region (*a*_1_), age (*a*_2_) and subtype (*a*_3_).

$$\begin{array}{ccl}{\rm{Therefore}},\; {\rm{for}}\; {\rm{CT}} & = & {\rm{oligodendrocytes}},\\ & & {{st}}_{{{\mathrm{CT}}}} \sim a_{\mathrm{1}}.a_{\mathrm{2}}.a_{\mathrm{3}}a_{\mathrm{1}}\in \left\{{{\mathrm{visual}}\,{\mathrm{cortex}}},{{\mathrm{striatum}}}.\,.\right\},\\ & & \qquad\qquad\qquad\quad a_{\mathrm{2}}\in \{{\mathrm{P14}},{\rm{P21}},.\,.\}\\ & & \qquad\qquad\ {{\mathrm{where}}}\,a_{\mathrm{3}}\in \left\{{{\mathrm{OPCs}}},{{\mathrm{COPs}}},.\,.\right\}\end{array}$$

Per gene and cell type (CT), a matrix (G) can be constructed with *i* rows containing the isoforms of the gene and *j* columns containing the cell clusters. So,

$$G \sim {g}_{{\mathrm{i,}}\;{\mathrm{j}}}\,{{\mathrm{where}}}\,j\in {{st}}_{{{\mathrm{CT}}}}\,{{\mathrm{and}}}\,i\,{{\mathrm{is}}}\,{{\mathrm{an}}}\,{{\mathrm{isoform}}}\,$$

So, for a brain region (*x*), a subset of the matrix can be obtained containing only the subtypes originating from that brain region,

$${G}^{{\prime} } \sim {g}_{{\mathrm{i}},\;{{\mathrm{j}}}^{{\prime} }}{{\mathrm{where}}}\,j{\prime} \sim x.a_{\mathrm{2}}.a_{\mathrm{3}}$$

Thus, for a cell type (CT), the brain region (BR) variability for isoform (*i*) is defined as

$$\begin{array}{l}{{\mathrm{isoVar}}}_{{\rm{i}}{\rm{\_}}{{\mathrm{CT}}}{\rm{\_}}{{\mathrm{BR}}}}=\max \left(\overline{{\Pi }_{{\rm{a}}_1}}\right)-\min \left(\overline{{\Pi }_{{\rm{a}}_1}}\right)\,{{\mathrm{where}}}\end{array}$$

$$\dot{{\Pi }_{{\rm{a}}_1={\rm{x}}}}=\frac{\sum _{{\rm{k}}\in {\rm{j}}{\prime} }{G{\prime} }_{{\rm{i}},{\rm{k}}}}{\sum {{\rm{G}}{\prime} }_{{\rm{x}}}}\,$$

Similarly, the age variability is defined as

$$\begin{array}{l}{{\mathrm{isoVar}}}_{{\rm{i}}{\rm{\_}}{{\mathrm{CT}}}{\rm{\_}}{{\mathrm{age}}}}=\max \left(\overline{{\Pi }_{{\rm{a}}_2}}\right)-\min \left(\overline{{\Pi }_{{\rm{a}}_2}}\right)\,{{\mathrm{where}}}\end{array}$$

$$\dot{{\Pi }_{{\rm{a}}_2={\rm{y}}}}=\frac{\sum _{{\rm{k}}\in {\rm{j}}{\prime} }{G{\prime} }_{{\rm{i}},{\rm{k}}}}{\sum {G}_{{\rm{y}}}}$$

and the subtype variability is defined as

$$\begin{array}{l}{{\mathrm{isoVar}}}_{{\mathrm{i}}{\rm{\_}}{{\mathrm{CT}}}{\rm{\_}}{{\mathrm{subtype}}}}=\max \left(\overline{{\Pi }{_{{a_{3}}}}}\right)-\min \left(\overline{{\Pi }_{{a_{3}}}}\right)\,{{\mathrm{where}}}\end{array}$$

$$\dot{{\Pi }_{{\rm{a}}_3={\rm{z}}}}=\frac{\sum _{{\rm{k}}\in {\rm{j}}{\prime} }{G{\prime} }_{{\rm{i}},{\rm{k}}}}{\sum {G}_{\rm{z}}}\,$$

Thus, the raw isoform variability for a cell type (CT) is

$${{\mathrm{isoVar}}}_{{\mathrm{i}}{\rm{\_}}{{\mathrm{CT}}}}=[{{\mathrm{isoVar}}}_{{\mathrm{i}}{\rm{\_}}{{\mathrm{CT}}}{\rm{\_}}{{\mathrm{BR}}}},{{\mathrm{isoVar}}}_{{\mathrm{i}}{\rm{\_}}{{\mathrm{CT}}}{\rm{\_}}{{\mathrm{age}}}},{{\mathrm{isoVar}}}_{{\mathrm{i}}{\rm{\_}}{{\mathrm{CT}}}{\rm{\_}}{{\mathrm{subtype}}}}]$$

For each isoform, we thus had a raw value for age, region and subtype variability. If an isoform did not have a reported variability value for any of the three axes, that isoform was excluded from further analysis. If any of these values were at least 0.1, that is, exhibiting at least a 10% change in isoform usage across an investigated axis, then the values were normalized to add up to 1 and represented in the ternary plot. For values where the normalized regional variability was greater than 0.5 but the age and subtype variability was less than 0.5, then the isoform was considered to be region specific for a particular cell type.

### Bootstrapping to evaluate cell-type differences in isoform variability

To reduce bias in our comparisons between cell types, reads were downsampled per gene for every cluster considered within an axis to have exactly ten reads. Thus, although it is possible to have a variable number of cell subtypes and time point clusters for different cell types, the *Π* calculation is performed on an equal number of reads. Variability per isoform and axis was performed as described above, and a matrix was obtained per iteration. One hundred such iterations were performed. To test if excitatory neurons exhibited variability not recapitulated in other cell types, that is, to test if the number of genes with isoforms in multiple (greater than or equal to two) triangles was greater than that for other cell types, we performed a Fisher’s exact test by constructing a 2 × 2 contingency matrix of counts for each iteration. This was performed per cell type compared to excitatory neurons. We then counted the number of iterations wherein the *P* value was significant and reported the summarized statistic.

### Obtaining full-length isoform variability across three axes in pseudobulk

A similar analysis to the one described above was performed, except that the cell subtype axis was replaced by a cell-type axis. Therefore, to find regional variability, we first calculated *Π* for each isoform in a region by summing the inclusion values over all cell types and time points for that region, and the variability values were obtained by subtracting the min(*Π*) from the max(*Π*). Age and cell-type variability were calculated similarly.

### Differential isoform expression analysis

#### Between-replicate variability estimation using downsampling

For each of the five brain regions, we obtained *Π* values of isoform inclusion per cell type and replicate. For each cell type, we selected genes wherein there were at least 100 reads per gene for both replicates. For each gene, we subsampled 50 reads from both replicates. To perform differential isoform expression analysis, we used the two-sample framework using *χ*^2^ tests of abundance, as described in scisorseqr^32^, between replicates. *P* values from a *χ*^2^ test were reported per gene, along with a Δ*Π* value per gene. The Δ*Π* value was constructed as the sum of change in percent isoform (*Π*) of the top two isoforms in either the positive or negative direction. After these numbers were reported for all testable genes for a comparison, the Benjamini–Hochberg correction for multiple testing with a false discovery rate of 5% was applied to return a corrected *P* value. If this false discovery rate *P* value was ≤0.05, then the percentage of significant genes was reported for Δ*Π* values ranging between 0.1 and 0.5. For each value of Δ*Π*, the average percentage of significant genes across all five brain regions was reported. This process was repeated 100 times to obtain a confidence interval. This gave us an idea of within-sample variability in isoform expression.

To obtain intersample (that is, regional) differences, we repeated the same process (downsampling followed by testing) for each cell type by considering one brain region and comparing it to the aggregated counts across the other four brain regions. This process was also repeated 100 times to get an estimate of intersample variability.

Cell types wherein the intrasample differences were much lower than the intersample differences were considered for further differential expression analysis.

#### Differential isoform expression for one brain region versus all others

We performed the same testing framework as described above by comparing one brain region to all others on all reads and reported the corrected *P* values and Δ*Π* per cell type.

#### Differential TSS and poly(A) expression for one brain region versus all

For each sequenced long read, we assigned a known CAGE peak if the start of the read fell within 50 bp of an annotated peak. Similarly, we assigned a poly(A) site to a read if it fell within 50 bp of a known site. Counts for each known TSS and poly(A) site were obtained per cell type, and reads where a TSS/poly(A) site could not be assigned were discarded. Counts for brain region comparisons were aggregated in a similar fashion to the full-length transcript analysis described above to obtain an *n* × 2 matrix. Differential isoform expression was performed using scisorseqr, and the Δ*Π* was recorded for each gene.

### Obtaining exon counts using corrected splice sites

Using all exons appearing as internal exons in a read, we calculated the following:

1. The number of long-read molecules containing this exon with identity of both splice sites: *X*_in_
2. The number of long-read molecules assigned to the same gene as the exon, which skipped the exon and ≥50 bases on both sides: *X*_out_
3. The number of long-read molecules supporting the acceptor of the exon and ending on the exon: *X*_acc_In_
4. The number of long-read molecules supporting the donor of the exon and ending on the exon: *X*_don_In_
5. The number of long-read molecules overlapping the exon: *X*_tot_

Nonannotated exons with one or two annotated splice sites, ≥70 bases of nonexonic (in the annotation) bases, were excluded as intron-retention events or alternative acceptors/donors.

We then calculated

- $${\varPsi }_{{{\mathrm{overall}}}}=\displaystyle\frac{{X}_{{{\mathrm{in}}}}+{X}_{{{\mathrm{acc}}}{\rm{\_}}{{\mathrm{In}}}}+{X}_{{{\mathrm{don}}}{\rm{\_}}{{\mathrm{In}}}}\,}{{X}_{{{\mathrm{in}}}}+{X}_{{{\mathrm{acc}}}{\rm{\_}}{{\mathrm{In}}}}+{X}_{{{\mathrm{don}}}{\rm{\_}}{{\mathrm{In}}}}+{X}_{{{\mathrm{out}}}}}$$
- $${\varPsi }_{{{\mathrm{acceptor}}}}=\displaystyle\frac{{X}_{{{\mathrm{in}}}}+{X}_{{{\mathrm{acc}}}{\rm{\_}}{{\mathrm{In}}}}\,}{{X}_{{{\mathrm{in}}}}+{X}_{{{\mathrm{acc}}}{\rm{\_}}{{\mathrm{In}}}}+{X}_{{{\mathrm{out}}}}}$$
- $${\varPsi }_{{{\mathrm{donor}}}}=\displaystyle\frac{{X}_{{{\mathrm{in}}}}+{X}_{{{\mathrm{don}}}{\rm{\_}}{{\mathrm{In}}}}\,}{{X}_{{{\mathrm{in}}}}+{X}_{{{\mathrm{don}}}{\rm{\_}}{{\mathrm{In}}}}+{X}_{{{\mathrm{out}}}}}$$
  If
- $0.05\le {\varPsi }_{{{\mathrm{condition}}}}\le 0.95$, where condition ∈ {overall, acceptor, donor}
- $$\displaystyle\frac{{X}_{{{\mathrm{in}}}}+{X}_{{{\mathrm{acc}}\_{\mathrm{In}}}}+{X}_{{{\mathrm{don}}\_{\mathrm{In}}}}+{X}_{{{\mathrm{out}}}}}{{X}_{{{\mathrm{tot}}}}}\ge 0.8$$

the exon was kept.

We then calculated the *Ψ*_overall_ for each cell type from all long-read unique molecular identifiers for that cell type if and only if *X*_tot_ ≥ 10 for the exon and cell type in question. Otherwise, *Ψ*_overall_ for the exon and cell type was set to ‘NA’.

### Identifying hVExs

We first obtained a matrix of *Ψ* values for all major cell types for each of the 11 samples by summing the counts over replicates. This yielded 44 triads defined by the age, region and cell type of origin. We then considered four lineages to calculate differences of exon inclusion values (Δ*Ψ* values) across the following:

- For a matched cell type and region, the developmental time-specific changes in exon inclusion were obtained by calculating pairwise Δ*Ψ* values. All comparisons that yielded Δ*Ψ* ≥ 0.25, that is, showed a 25% change in inclusion for an exon between two time points, were reported.
- For a given time point and region, the cell-type-specific changes in exon inclusion were obtained by calculating pairwise Δ*Ψ* values. All comparisons that yielded Δ*Ψ* ≥ 0.25 were reported.
- For a matched cell type at P56, adult brain region-specific changes in exon inclusion were obtained by calculating pairwise Δ*Ψ* values, and comparisons yielding Δ*Ψ* ≥ 0.25 were reported.
- For a given brain region at P56, the cell-type-specific changes in exon inclusion were obtained by calculating pairwise Δ*Ψ* values, and comparisons yielding Δ*Ψ* ≥ 0.25 were reported.

The exons obtained from the four lineages described above were classified as hVExs. The Δ*Ψ* value for all pairwise comparisons of 44 triads, that is, for 946 comparisons, were then calculated for these hVExs and reported. To enable hierarchical clustering of this matrix of comparisons × exons, exons with too many NA values due to lack of depth for *Ψ* calculations in many triads were filtered out.

### Identifying EVExs

For the exons classified as highly variable (see above) and for each of the four lineages considered, we retained the comparison with the highest Δ*Ψ* value. This yielded a matrix with four columns, one for each of the lineages, and 5,931 rows, 1 for each hVEx. Of these, exons with Δ*Ψ* ≥ 0.75 in any of the four columns were retained. These were defined as EVExs, wherein at least one comparison between triads across the four lineages displayed 75% or more change in exon inclusion.

### Mapping orthologous exons in human data

The TransMap^67^ projection alignment algorithm was used to map exons between human and mouse assemblies. LASTZ^68^ genomics alignments between the human GRCh38 and mouse GRCm39 reference assemblies were used to map reference transcript annotations between assemblies. TransMap was used instead of University of California Santa Cruz Genome Browser liftOver^69^ as it produces base-level alignments, allowing observation of insertions/deletions and other differences between the LASTZ chain and net alignments files^70^. These were obtained from the University of California Santa Cruz Genome Browser site, along with the below-mentioned programs to process them. Syntenic genomic alignments were obtained by filtering the net files to obtain the syntenic nets using ‘netFilter -syn’ and then using ‘netChainSubset -wholeChains’ to obtain a set of syntenic chain alignments for mappings. GENCODE^71^ human v35 and mouse vM26 were mapped to the other assembly using the pslMap program.

### Obtaining cell-type variability in the human hippocampus

We obtained *Ψ* values for each cell type in the human data using the same strategy as for the mouse data. Orthologous exons that were unambiguously and reciprocally mapped between humans and mice and shared sequence homology and length were selected. For EVExs in groups E1–E5 identified in mice, exon *Ψ* values were obtained per cell type for exons where sufficient depth for the exon’s ortholog was available. Because only one brain region (hippocampus) and time point (adult) were available, variability was defined as the max(*Ψ*) – min (*Ψ*) among the major cell types. Similarly, for all exons in the human data that had orthologs in mice, *Ψ* values were obtained per major cell type, and exons were classified as ‘highly’ variable if eVar ≥ 0.5 and invariable but alternative if eVar ≤ 0.2. To allow for a higher number of exons to be queried, we then obtained the *Ψ* values for the broad categories of neurons and glia from both mouse and human data and reported them.

### Effects of RBP on exon inclusion

We accessed ENCODE III data from two human cell lines, K562 and HepG2, wherein short hairpin RNA or CRISPR was used to deplete individual RBPs, followed by RNA sequencing of both the KD and control samples^56,57^. A total of 263 RBPs were profiled in this study, and about one-third were annotated as being involved in splicing regulation and RNA processing. We obtained data processed with rMATs and identified cassette exons that were significantly altered (*P* ≤ 0.05 and change in percent spliced index ≥ 0.1) in their splicing profiles from all the RBP KD experiments and recorded both the exon and the associated RBP. We intersected these exons with the human orthologs of the mouse EVExs. We could thus characterize the number of exons that were influenced by RBP expression and the number of RBPs that influenced each exon. More information is available in Supplementary Note 5.

### Identifying sQTLs associated with exons of interest

We accessed the GTEx v8 (ref. ^58^) genome-wide association study (GWAS) catalog and obtained a list of sQTLs that were associated with intron removal ratios for brain regions included in our study. From our mouse data, we isolated exons that either flank or are contained within these significant introns identified by GTEx. Finally, we cross-referenced the genes within which these single-nucleotide polymorphisms were located with the GWAS catalog and manually identified the list of genes that are associated with central nervous system traits.

### Obtaining developmental modalities of variability

Considering the second of the four lineages above, that is, the cell-type-specific differences in exon inclusion for a given time point and brain region, we calculated exon variability. For exons where we had sufficient depth to calculate the *Ψ* values for at least two cell types, we calculated the exon variability as the max(*Ψ*) – min(*Ψ*) for each time point. Thus,

$$\begin{array}{l}{{{\mathrm{eVar}}}}_{{{\mathrm{TP}}}}=\max \left(\overline{{\rm{PSI}}}\right)-\min \left(\overline{{\rm{PSI}}}\right){{\mathrm{where}}}\,{\rm{PSI}} \\\sim [{\Psi }_{{\rm{Astro}}},{\Psi }_{{\rm{Oligo}}},{\Psi }_{{\rm{ExciteNeuron}}},{\Psi }_{{\rm{InhibNeuron}}}]\end{array}$$

We then considered the first lineage from the hVEx paradigm described above, that is, developmental time-specific changes. Here, we looked at the change in variability between time point transitions, that is, from P14 to P21, from P21 to P28 and from P28 to P56. If the change in eVar (Δ_eVar_) was less than 0.1 in all three transitions, indicating less than a 10% change in cell-type-specific variability over time, then the exon was classified as invariable (Supplementary Fig. 21).

$$\begin{array}{l}\Delta {{\mathrm{eVar}}}={{{\mathrm{eVar}}}}_{{\mathrm{x}}}-{{{\mathrm{eVar}}}}_{{\mathrm{y}}}\,{{\mathrm{where}}}\,x > y\,{{\mathrm{and}}}\,x,y\in \{{\mathrm{P14,P21,P28,P56}}\}\\{\Delta {{\mathrm{eVar}}}}_{{{\mathrm{comp}}}}\le 0.1\forall {{\mathrm{comp}}}\in \left({\mathrm{P14}}\to {\mathrm{P21,P21}}\to {\mathrm{P28,P28}}\to {\mathrm{P56}}\right) \\\sim {{ \mbox{`} }}\text{Invariable}\,{{\mathrm{exon}}}{{\mbox{'}}}\end{array}$$

Otherwise, the exon was classified as variable. *Z* scores were calculated per row of the matrix of exon × eVar_TP_ by centering and scaling the data to have the mean at 0 and standard deviation of 1. This normalized matrix was used for clustering and obtaining nine developmental modalities.

### Getting protein superfamily annotations per exon

We developed a computational pipeline to identify protein superfamily annotations per exon. For each exon, we used the genomeToProtein() function of the ensembldb package and extracted the Ensembl ID, coordinates, and residue sequence of the protein identified. We filtered the obtained protein identifiers based on their corresponding Ensembl transcript IDs and limited the search to the principal isoforms from the APPRIS^72^ database. For each protein sequence, we ran the SUPERFAMILY^73,74^ tool that uses the hidden Markov model to identify the structural-defined SCOP protein domain families and the domain boundaries. The tool was implemented in InterProScan^75–77^. Protein regions not associated with domains were considered interdomain linkers. Subsequently, for each exon, the superfamily annotation associated with the protein residue for those coordinates was identified and used for further analysis.

### Enrichment of protein superfamily annotations

We considered EVExs in clusters E1–E5 (Fig. 3) as well as a background set consisting of exons with low variability across the entire dataset. For exons that had a domain associated with the coordinates of the coding sequence, we extracted the superfamily rather than individual and similar domains, given that the broader classification would allow for better grouping. We then counted the number of exons associated per superfamily and group and reported a percentage. The superfamilies for which a value was obtained only for the background set were discarded, yielding a total of 55 superfamilies. For better interpretability, we retained superfamilies that were associated with at least 4% enrichment in any group, yielding a total of 15 superfamilies.

### GO analysis for genes associated with variable exon categories

For exons in the four hVEx categories (H1–H4), genes to which these exons belonged were extracted per category. Only unique genes per category were retained, meaning that if two exons from a gene belonged to two different categories, the gene was discarded from the analysis. GO biological process enrichment analysis was performed using the function enrichGO() from the clusterProfiler^78^ package. GO terms with *q* values of ≤0.1 were reported, and the enrichment value was defined as the ratio of genes in the category being considered to those in the background. Similarly, for the invariable exons and the nine variable developmental categories (G0–G9), unique genes containing the exons in each category were identified. GO biological process analysis was performed as described above using a list of brain-expressed genes obtained from SynGO^79^ as the background set. GO terms with *q* values of ≤0.1 were reported, and the enrichment value was defined as the ratio of genes in the category being considered to those in the background. In the same vein, genes of adult brain region-specific EVExs (group E4) were identified, and the same steps were performed.

### Pseudotime trajectory analysis

Isoquant v3.1 was run on the ONT data, and full-length isoforms were grouped by barcode to obtain an isoform × cell sparse matrix similar to the CellRanger pipeline across the dataset. Cell barcodes corresponding to astrocytes and oligodendrocytes from the hippocampus and visual cortex developmental lineage were isolated, and a subset of a matrix containing these cellular barcodes as columns was obtained. This matrix was then processed using Seurat^36^ (v3.2.3) to obtain a UMAP representation of the cells. Each cell was colored according to the original short-read cell-type assignments. Slingshot (v1.6)^63^ was then used to obtain a pseudotime trajectory along these clusters, with the initial point specified at OPCs. Lineages obtained were then reported.

### Testing for exon coordination

Testing for exon coordination can be done at the pseudobulk level or at the cell-type level. For every exon pair passing the criteria for sufficient depth, a 2 × 2 matrix of association for a given sample (that is, cell type or pseudobulk) was generated. This matrix contained counts for inclusion of both exons (in–in), inclusion of the first exon and exclusion of the second (in–out), exclusion of the first exon and inclusion of the second (out–in) and exclusion of both exons (out–out).

The co-inclusion score of an exon was defined as the double inclusion (in–in) divided by the total counts for that exon pair. An exon pair deemed ‘coordinated’ was assessed using the *χ*^2^ test of association. The effect size was calculated as the | log_10_ (odds ratio) |. The odds ratio was calculated by setting 0 values to 0.5 and dividing the product of double inclusion and double exclusion by the product of single inclusion, that is, [(in–in) × (out–out)]/[(in–out) × (out–in)].

### Reporting summary

Further information on research design is available in the Nature Portfolio Reporting Summary linked to this article.

## Online content

Any methods, additional references, Nature Portfolio reporting summaries, source data, extended data, supplementary information, acknowledgements, peer review information; details of author contributions and competing interests; and statements of data and code availability are available at 10.1038/s41593-024-01616-4.

### Supplementary information

Supplementary Information Supplementary Tables 1–5, Figs. 1–27, Notes 1–7 and Methods.

Reporting Summary

## Data Availability

The summary of all mouse data used for this study is available on the Knowledge Brain Map at https://knowledge.brain-map.org/data/Z0GBA7V12N4J4NNSUHA/summary, and all human data are available at https://knowledge.brain-map.org/data/ASP3B09DZ8PXDUYSHDH/summary. These pages contain links to raw and processed data hosted on the Neuroscience Multi-Omic data archive under the identifier dat-717krsa (https://assets.nemoarchive.org/dat-717krsa). All data supporting the findings of this study are provided within the paper and its Supplementary Information. Publicly available data were downloaded from APPRIS (https://apprisws.bioinfo.cnio.es/landing_page/), ENCODE (https://www.encodeproject.org/), GTEx (https://www.gtexportal.org/home/downloads/adult-gtex/qtl) and the GWAS catalog (https://www.ebi.ac.uk/gwas/docs/file-downloads). Source data for the main figures can be found at https://github.com/noush-joglekar/biccn_tilgner_scisorseq/tree/main/data (ref. ^80^).

## Code availability

The source code generated for this paper is publicly available at https://github.com/noush-joglekar/biccn_tilgner_scisorseq (ref. ^81^).
