# Supplementary material for: Single-cell long-read sequencing-based mapping reveals specialized splicing patterns in developing and adult mouse and human brain
Source: Nat Neurosci. 2024 Apr 9;27(6):1051–63. doi: 10.1038/s41593-024-01616-4 (PMC11156538; doi:10.1038/s41593-024-01616-4)
Supplement: Supplementary file 1 — Supplementary Tables 1–5, Figs. 1–27, Notes 1–7 and Methods. [file 41593_2024_1616_MOESM1_ESM.pdf]

nature neuroscience

Article https://doi.org/10.1038/s41593-024-01616-4

Single-cell long-read sequencing-based mapping reveals specialized splicing patterns in developing and adult mouse and human brain

In the format provided by the authors and unedited

Single-cell long-read sequencing-based mapping reveals specialized splicing patterns in developing and adult mouse and human brain

Anoushka Joglekar, Wen Hu, Bei Zhang, Oleksandr Narykov, Mark Diekhans, Jordan Marrocco, Jennifer Balacco, Lishomwa C Ndhlovu, Teresa A Milner, Olivier Fedrigo, Erich D Jarvis, Gloria Sheynkman, Dmitry Korkin[4], M. Elizabeth Ross, Hagen U. Tilgner

Supplementary Materials

Contents

Supplementary Tables S1-S5	2
Supplementary Figures S1-S27	7
Supplementary Notes 1-7	32

Supplementary Tables S1-S5

Timepoint	Brain region	Replicate	Sample ID	Total cells	Cells after QC	Median genes per cell	Median UMIs per cell
P14	HIPP	Rep1	M5	14350	10046	2696	6042
		Rep2	M6	10180	6711	3120	7651
	VIS	Rep1	M5	13661	10191	1865	3415
		Rep2	M6	23317	19424	1673	3114
P21	HIPP	Rep1	M1	11695	9355	1530	2803
		Rep2	M5	13920	8271	2035	4560
	VIS	Rep1	M1	14231	10485	1272	2221
		Rep2	M5	8655	8253	2251	4849
P28	HIPP	Rep1	M1	13392	11067	2189	4746
		Rep2	M2	11882	10240	2077	4224.5
	VIS	Rep1	M1	15320	12553	2144	4271
		Rep2	M2	10046	8604	2504	5322
P56	HIPP	Rep1	M1	6906	6494	1919	4354
		Rep2	M2	12724	12124	1487	2839
	VIS	Rep1	M8	4733	4009	3285	8327
		Rep2	M9	9564	7676	2196.5	5001
	STRI	Rep1	M1	8206	7270	1767	3739
		Rep2	M2	7550	6618	2332	5073
	THAL	Rep1	M5	16145	13502	2116	4998
		Rep2	M6	18278	16474	1985	4415
	CEREB	Rep1	M1	3676	3339	2971	9210
		Rep2	M2	3870	3340	1993	3687

Supplementary Table 1: Sample details and quality control statistics for 10x single-cell short read sequencing libraries

Timepoint	Brain region	Replicate	Sample ID	Mean reads / SMRT cell	PolyA detected	Barcoded	Mean Length	CSMM Reads
P14	HIPP	Rep1	M5	2816040	88.73	46.56	898.67	67.11
		Rep2	M6	3921610	87.07	36.88	1002.27	66.98
	VIS	Rep1	M5	2599560	83.14	38.23	929.94	70.59
		Rep2	M6	3366700	91.43	49.33	887.56	68.78
P28	HIPP	Rep1	M1	2698694	81.98	51.42	1004.28	70.12
		Rep2	M2	3074310	88.25	55.96	992.39	67.03
	VIS	Rep1	M1	2820433	84.14	53.10	1059.71	69.07
		Rep2	M2	1990540	86.64	52.27	1012.07	68.34
P56	HIPP	Rep1	M1	2802050	91.03	70.28	1155.99	70.71
		Rep2	M2	2620720	80.59	69.51	1071.05	72.66
	STRI	Rep1	M1	2212459	87.66	70.75	1099.93	70.99
		Rep2	M2	2083750	84.68	67.96	1103.04	72.75
	CEREB	Rep1	M1	2755640	89.90	59.06	1125.40	72.91
		Rep2	M2	2705560	85.64	61.03	1137.35	65.39
	THAL	Rep1	M5	3724854	85.10	41.96	1012.66	72.56
		Rep2	M6	1349837	85.26	45.48	1026.63	71.79

Supplementary Table 2: Sample details and quality control statistics for unfragmented single-cell long-read libraries sequenced on Pacific Biosciences (PacBio) HiFi Sequel II

Timepoint	Brain region	Replicate	Sample ID	Total Reads	PolyA	Read length	Mapped and barcoded	CSMM
P14	HIPP	Rep1	M5	168303300	77.98	851.84	29959564	66.63
		Rep2	M6	73356979	79.64	951.89	12957198	65.44
	VIS	Rep1	M5	53348838	77.05	842.75	9794529	67.11
		Rep2	M6	104565321	80.78	823.28	12957198	65.44
P21	HIPP	Rep1	M1	97967471	73.42	857.60	22819033	66.42
		Rep2	M5	103148800	72.53	860.36	11736363	62.25
	VIS	Rep1	M1	68513664	73.67	860.39	17593692	66.32
		Rep2	M5	50020807	71.21	876.01	6831543	64.54
P28	HIPP	Rep1	M1	83183369	80.02	959.37	16422428	64.60
		Rep2	M2	56387308	87.41	889.43	15610708	59.57
	VIS	Rep1	M1	82152770	78.77	918.15	17636898	64.80
		Rep2	M2	85041433	86.52	908.46	21860630	60.53
P56	HIPP	Rep1	M1	55104959	84.5	946.97	16908061	70.74
		Rep2	M2	53430301	72.34	942.99	15552654	71.56
	VIS	Rep1	M8	76083018	81.31	968.55	21209929	74.06
		Rep2	M9	157171800	84.73	1063.09	52197443	76.58
	STRI	Rep1	M1	72719845	78.02	965.52	21342316	73.25
		Rep2	M2	58786362	74.27	953.77	16320143	71.25
	THAL	Rep1	M5	60842158	79.22	840.97	12024111	69.72
		Rep2	M6	77752175	77.68	870.98	14836416	69.43
	CEREB	Rep1	M1	42192772	81.94	962.18	10509899	74.01
		Rep2	M2	119513025	77.06	1014.49	30659173	65.77

Supplementary Table 3: Sample details and quality control statistics for unfragmented single-cell long-read libraries sequenced on Oxford Nanopore (ONT) PromethION

Timepoint	Brain region	Replicate	Sample ID	min Genes	max Genes	min UMIs	max UMIs	mito cutoff	dims	resolution
P14	HIPP	Rep1	M5	500	6000	1000	25000	20	30	0.6
		Rep2	M6	500	6000	1000	30000	30	30	0.6
	VIS	Rep1	M5	500	6000	1000	20000	20	30	0.6
		Rep2	M6	500	6000	1000	25000	30	30	0.6
P21	HIPP	Rep1	M1	500	5000	1000	15000	25	30	0.6
		Rep2	M5	500	6000	1000	25000	20	30	0.6
	VIS	Rep1	M1	500	6000	1000	18000	40	30	0.6
		Rep2	M5	1000	6000	1000	30000	30	25	0.6
P28	HIPP	Rep1	M1	500	6000	1000	25000	18	30	0.6
		Rep2	M2	500	6000	1000	25000	15	30	0.6
	VIS	Rep1	M1	500	6000	1000	25000	18	30	0.6
		Rep2	M2	500	7000	1000	30000	18	25	0.6
P56	HIPP	Rep1	M1	500	7000	1000	20000	15	30	0.6
		Rep2	M2	500	7000	1000	20000	15	30	0.6
	VIS	Rep1	M8	1000	7500	1000	40000	7	25	0.6
		Rep2	M9	1000	7500	1000	40000	10	25	0.6
	STRI	Rep1	M1	500	5000	1000	20000	15	25	0.6
		Rep2	M2	500	6000	1000	25000	18	25	0.6
	THAL	Rep1	M5	400	7500	1000	40000	35	30	0.6
		Rep2	M6	400	7500	1000	40000	35	30	0.6
	CEREB	Rep1	M1	500	6000	1000	25000	20	20	0.5
		Rep2	M2	500	6000	1000	25000	20	20	0.5

Supplementary Table 4: Parameters used in the processing of single-cell short read data

Age	Hemisphere	Subject Sex	Race	PMI (hours)	RIN	Clinical Brain Diagnosis
28-40	Left	Female	Black or African-American	20	99.99	Unaffected Control
28-40	Left	Male	White	15	7	Unaffected Control
28-40	Left	Female	White	23	7.1	Unaffected Control
28-40	Left	Male	White	24	8.3	Unaffected Control
28-40	Left	Female	Black or African-American	15	7.1	Unaffected Control
28-40	Left	Male	White	7	8.3	Unaffected Control

Supplementary Table 5: Subject details for single-nuclei hippocampal samples

Supplementary Figures S1-27

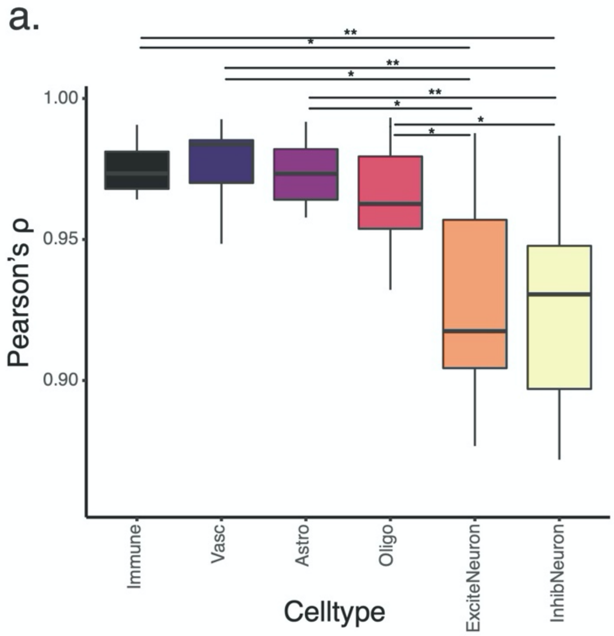

Supplementary Figure 1: Heterogeneity of neurons and glia. Boxplot showing the pairwise pearson correlation in gene expression of individual cell types. Distribution represents n=10 correlation values obtained from gene expression values. Center line, median; box limits, upper and lower quartiles; and whiskers, 1.5× interquartile range. Wilcoxon two-sided test p-value indicated for distributions of neuronal types versus glial types (*: p<0.05, **:p<0.005)

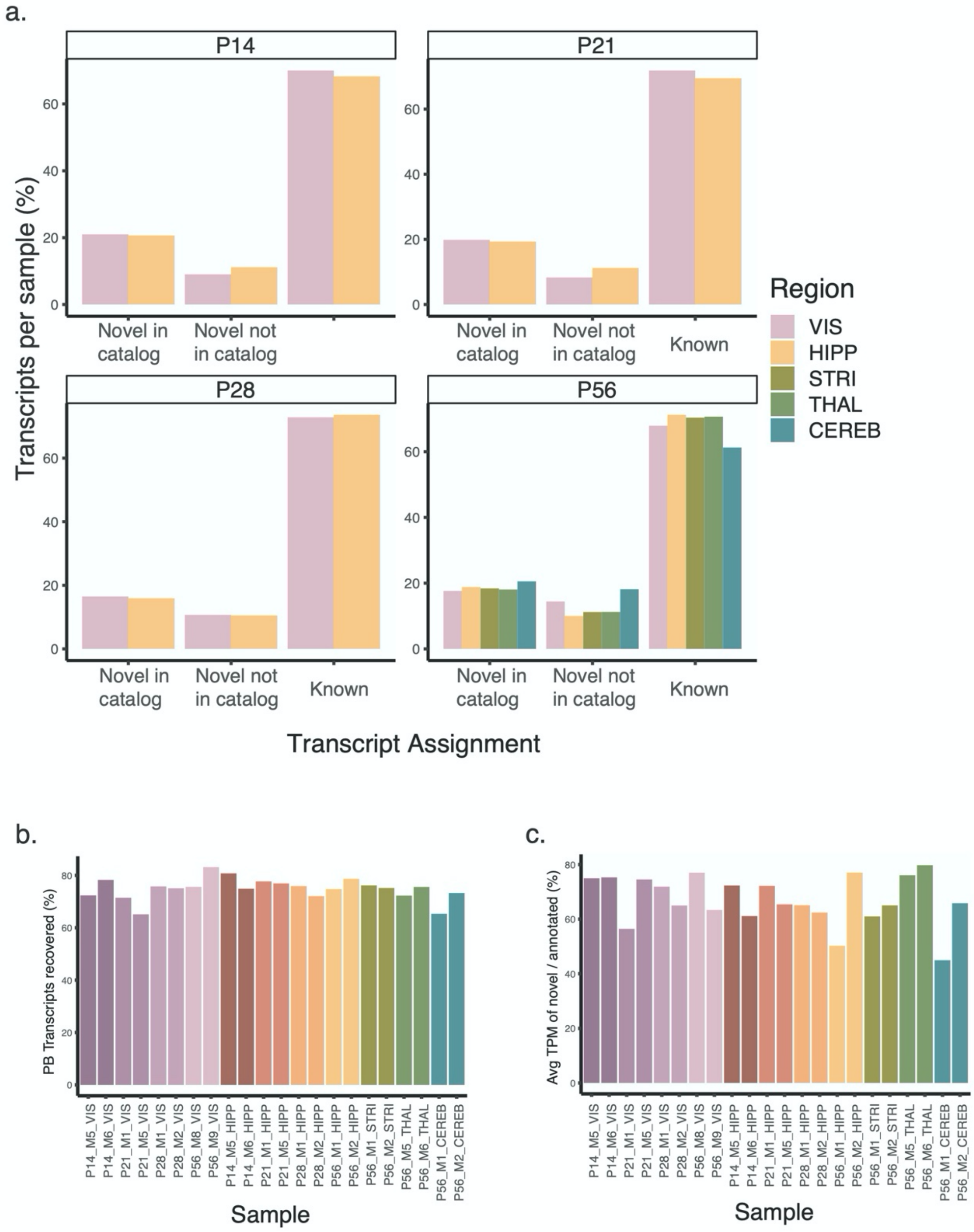

Supplementary Figure 2: Long read transcript assignment and novelty. (a) Barplots of the percentage of transcript in each sample, averaged over replicates, and classified by IsoQuant as novel in catalog, novel not in catalog or Known. Color of bar indicates region of origin. Each sub-panel represents the developmental timepoint from which the samples were collected. **(b)** Percentage of novel PacBio isoforms that were recovered by samples indicated on the x-axis **(c)** Barplot of the ratio of the average TPM of isoforms classified as novel to annotated isoforms in each sample indicated on the x-axis.

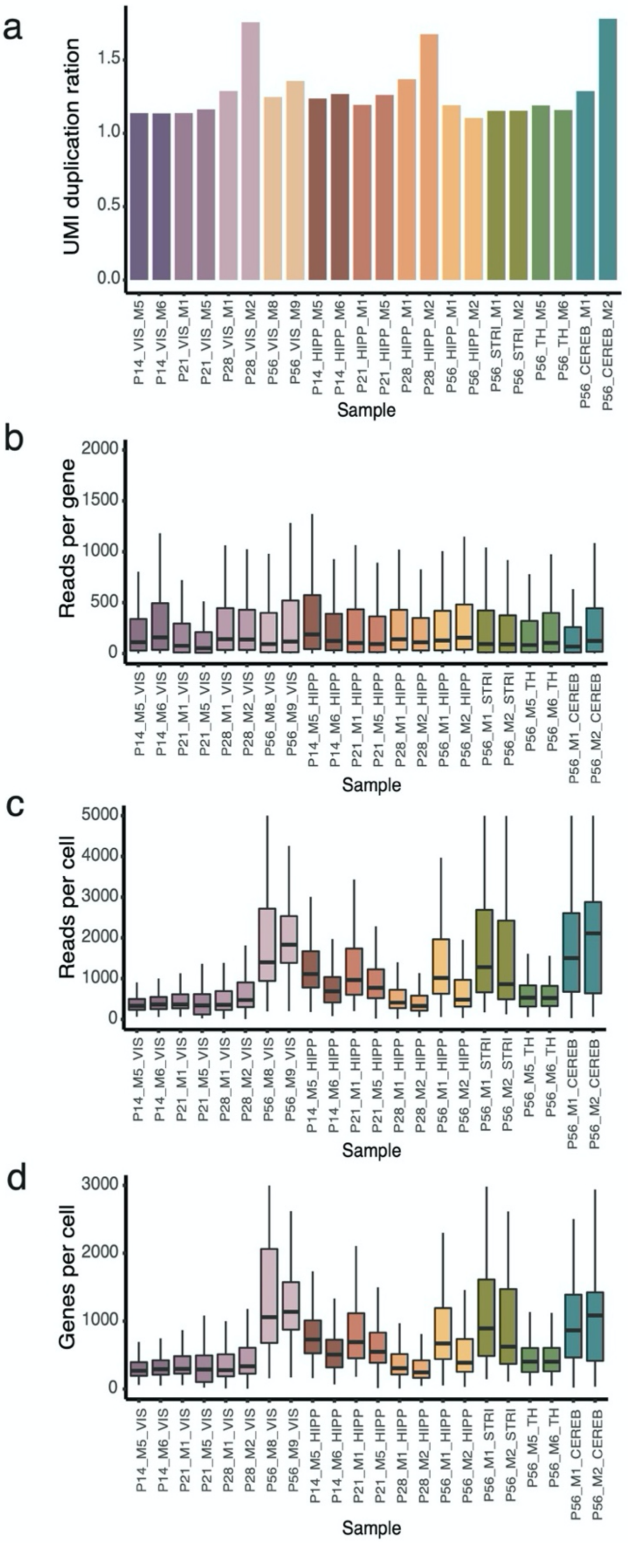

Supplementary Fig 3: Quality control for ONT long reads. (a) Barplot of the UMI duplication ratio per sample indicated on the x-axis. **(b)** Boxplot of the number of spliced, barcoded, umi deduplicated reads per gene in each sample **(c)** Same as (b) but with reads per cell in each sample. **(d)** Same as (b) but with number of genes per cell in each sample. Color of bar represents sample of origin, each replicate has the same color. N=11 samples with 2 biological replicates each were used. For cell numbers refer to Table S1. For boxplots: center line, median; box limits, upper and lower quartiles; and whiskers, 1.5× interquartile range.

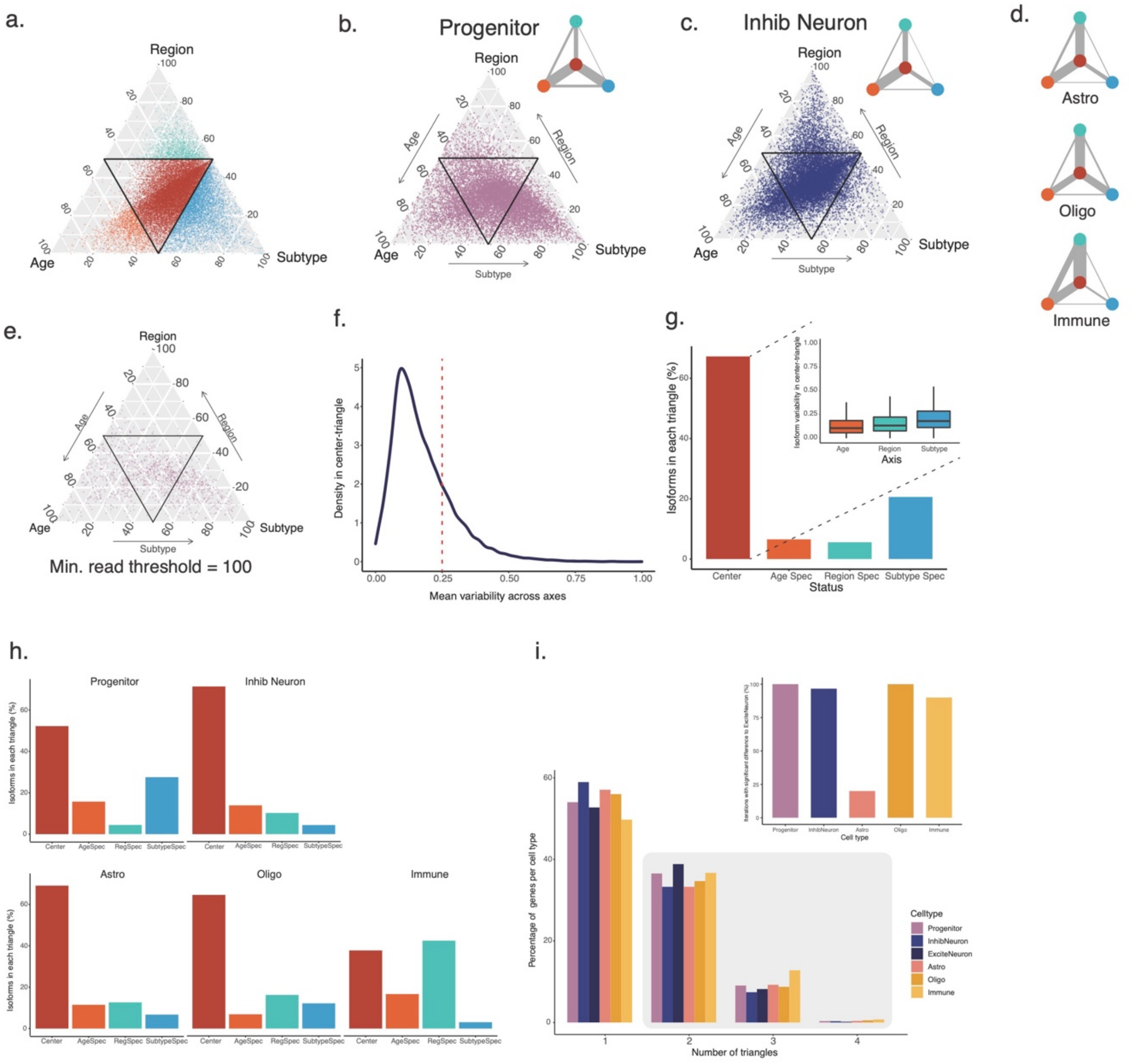

Supplementary Fig 4: Isoform variability in cell types for full-length isoforms. (a) Categorization of isoforms as being age (orange), subtype (blue), region (teal) specific depending on localization in the outer triangles, or as center triangle (red) isoforms as having equally low or high variability. **(b)** Ternary plot of Isoform variability for progenitors. *Inset* Network diagram showing genes with two or more isoforms in distinct triangles. Nodes represent variability type as described in (a). Edge represents number of genes with isoforms in the two triangles represented by the nodes. **(c)** Same as in (b) but for inhibitory neurons. **(d)** Network diagrams for astrocytes, oligodendrocytes, and immune cells **(e)** Same as in (b) but with an imposed minimum read threshold of 100 reads for each axis considered. **(f)** Density of mean variability across three axes for each isoform in the center triangle for excitatory neurons **(g)** % of total isoforms in each triangle of variability (n = 20975, 2044, 1740, 6430 isoforms derived from 11 samples with 2 biological replicates for each sample) for excitatory neurons. *Inset* Variability values for each axis within the center triangle. Center line, median; box limits, upper and lower quartiles; and whiskers, 1.5× interquartile range **(h)** Same as in (f) but for all other cell types. **(i)** % of genes with isoforms in 1, 2, 3, or 4 triangles of variability. *Inset* Bar plot showing % of downsampling experiments (n = 100) wherein number of genes with isoforms in multiple quadrants (grey shaded area in i) was significantly higher in excitatory neurons than other cell types. Significance: Fisher two-sided exact p-value < 0.05. No correction for multiple testing was performed.

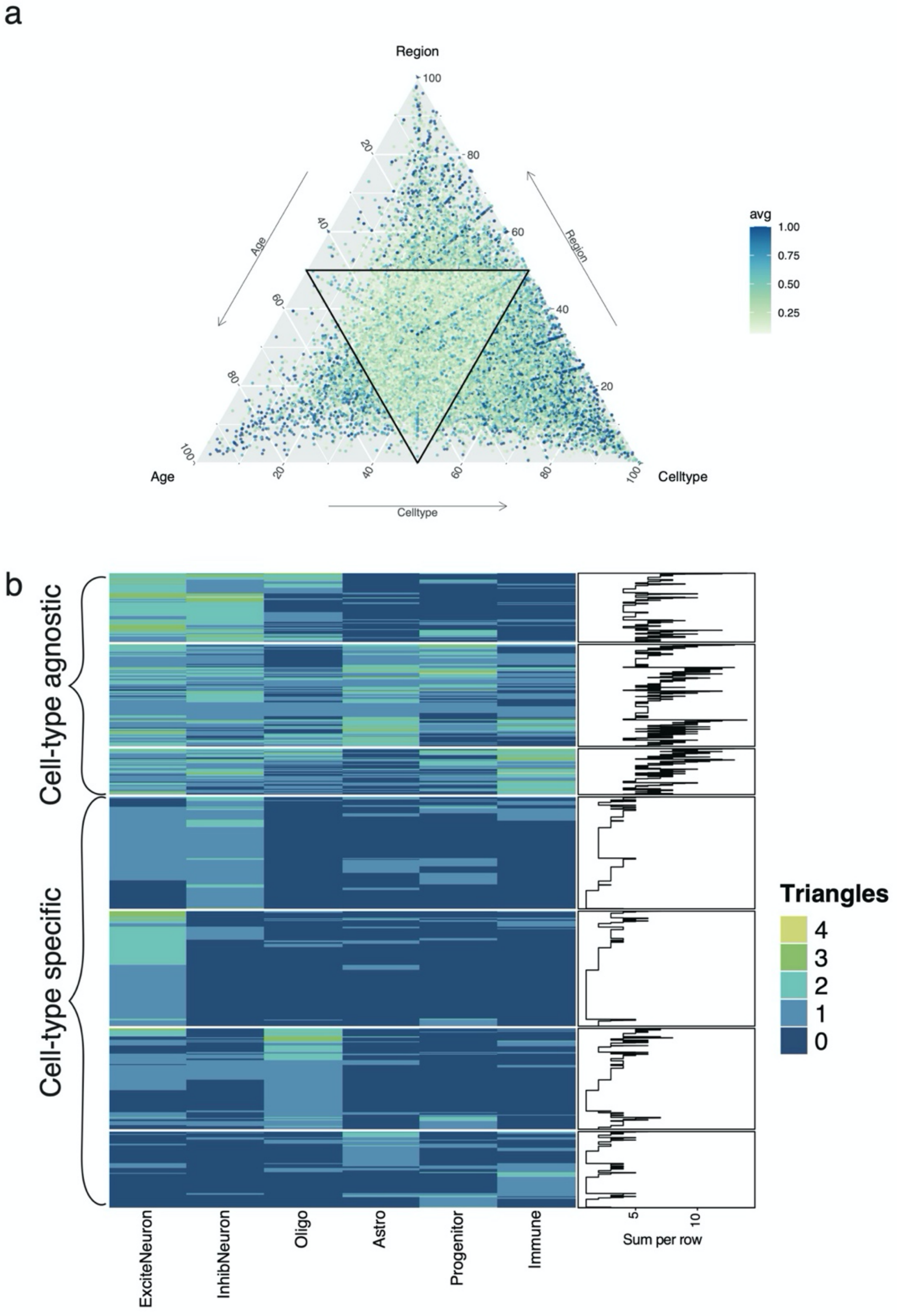

Supplementary Fig 5: Isoform variability in pseudobulk and hypervariable genes (a) Ternary plot of the isoform variability in pseudobulk with triangles representing age, region, or cell type specificity per isoform at each vertex. Each point is a single isoform, and color of point represents the average raw variability value along all three axes. **(b)** Heatmap of hypervariable genes per cell type. Color of each tile in the heatmap indicates the number of triangles in which an isoform was found to be highly variable (variability ≥ 0.25) for a given cell type. Annotation bar on the right denotes the line plot of the sum of the number of triangles per row. High values indicate genes wherein isoforms are regulated along multiple axis along multiple cell types and are therefore highly variable and cell-type agnostic. Low values / non-zero values in a few cell-types indicates highly specialized isoforms with cell-type specificity.

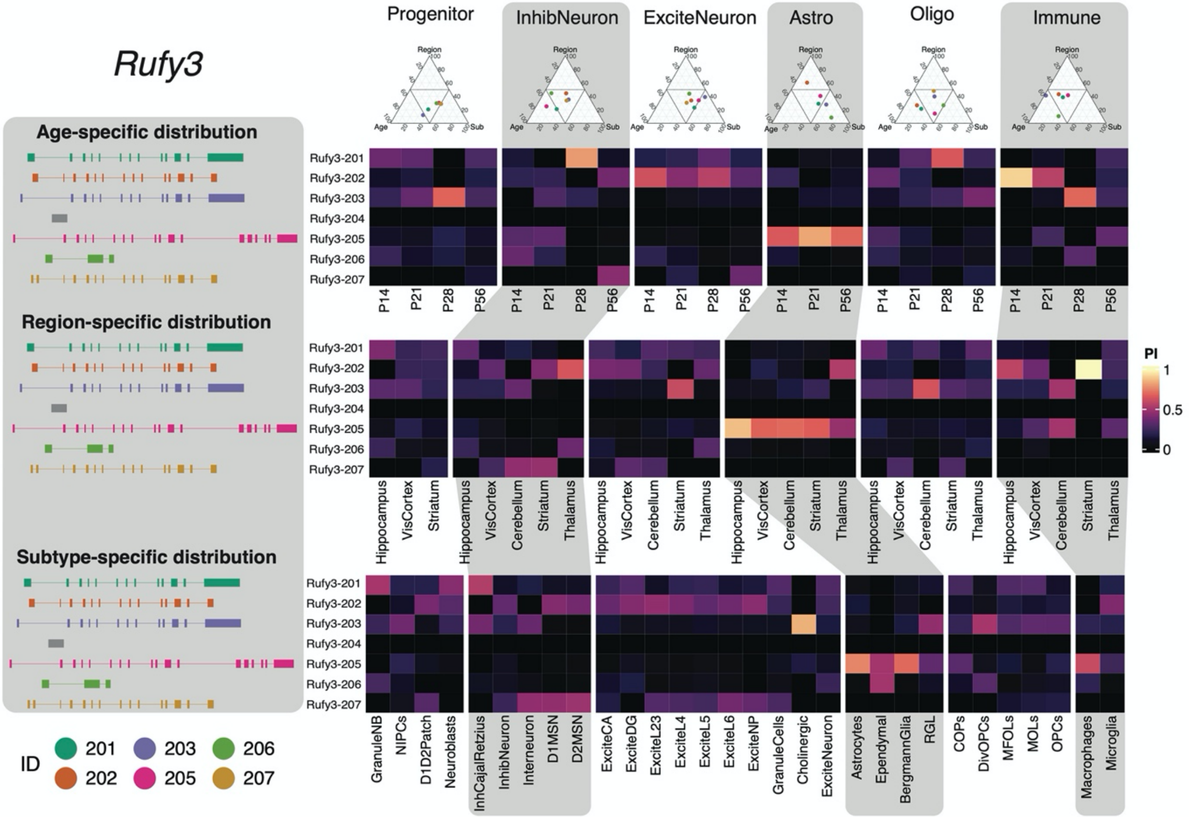

Supplementary Fig 6: Isoform regulation in *Rufy3.* Left panel in grey showing seven annotated isoforms of the gene *Rufy3* with three sections indicating age, region, and subtype specific distributions to be represented in the heatmaps on the right. Ternary plots on the right are shown per cell type, with the isoform variability value per isoform represented in the plot, where each point represents a *Rufy3* isoform colored according to the legend on the bottom left. Each heatmap shows an isoform (rows) per cell-type in each developmental stage as columns (top section), each brain region as columns (middle section) and each cell subtype as columns (bottom section). Color of tile shows the percent inclusion (Π).

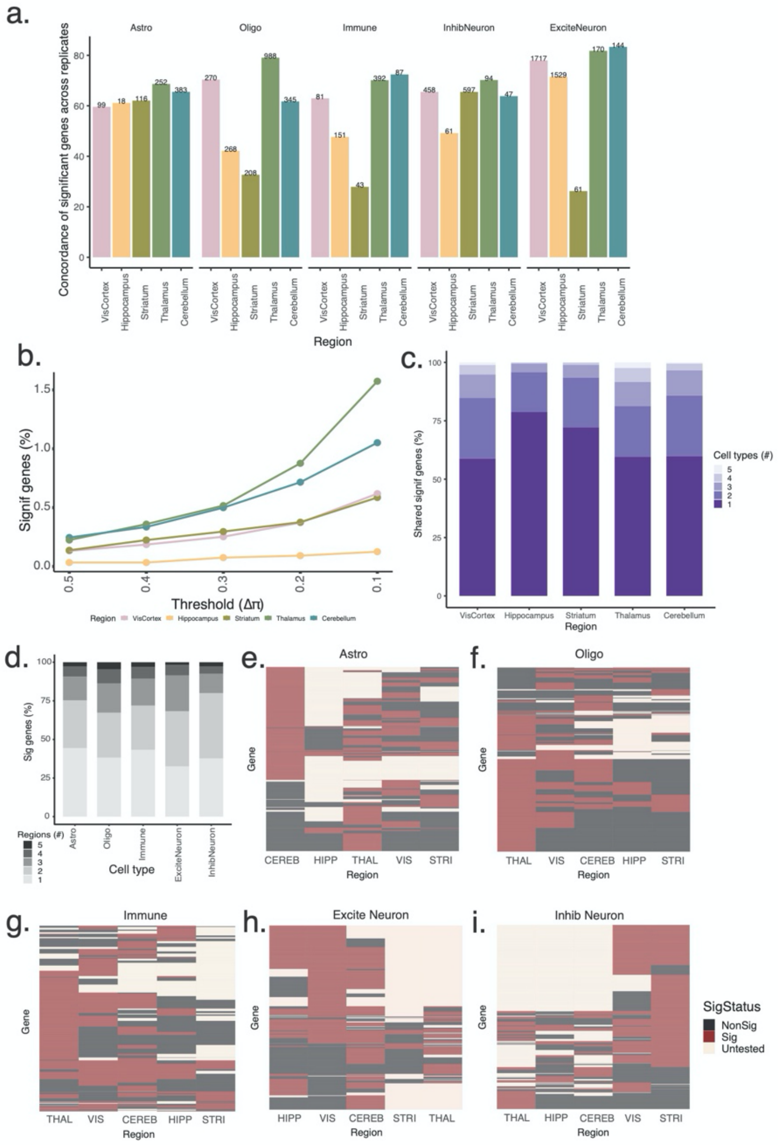

Supplementary Fig 7: (a) Barplot of % of commonly tested genes in replicate 1 and replicate 2 that are significant in both replicates. Count of commonly tested genes represented on top of each bar (**b**) % of genes showing significant differences in isoform expression between one brain region vs. all other brain regions at 5 ΔΠ cutoffs **(c)** Stacked barplot of % of significant cell-type and region-specific genes that are significant in one cell type (dark purple) for that region or shared between 2,3,4,5 cell types (lighter purples). **(d)** % of genes with significant differential isoform expression ($\Delta\Pi \geq 0.1$) that are unique to one (light grey) or shared between multiple (darker grays) brain regions for each cell type. (**e**) Heatmap showing status (black–NonSig, Tan–Untested, Maroon–Significant) for each gene with significant differential isoform usage in one brain region compared to all others for astrocytes cells. Foreground region indicated on the x-axis. (**f-i**) Same as in (e) for oligodendrocytes, immune cells, excitatory, and inhibitory neurons.

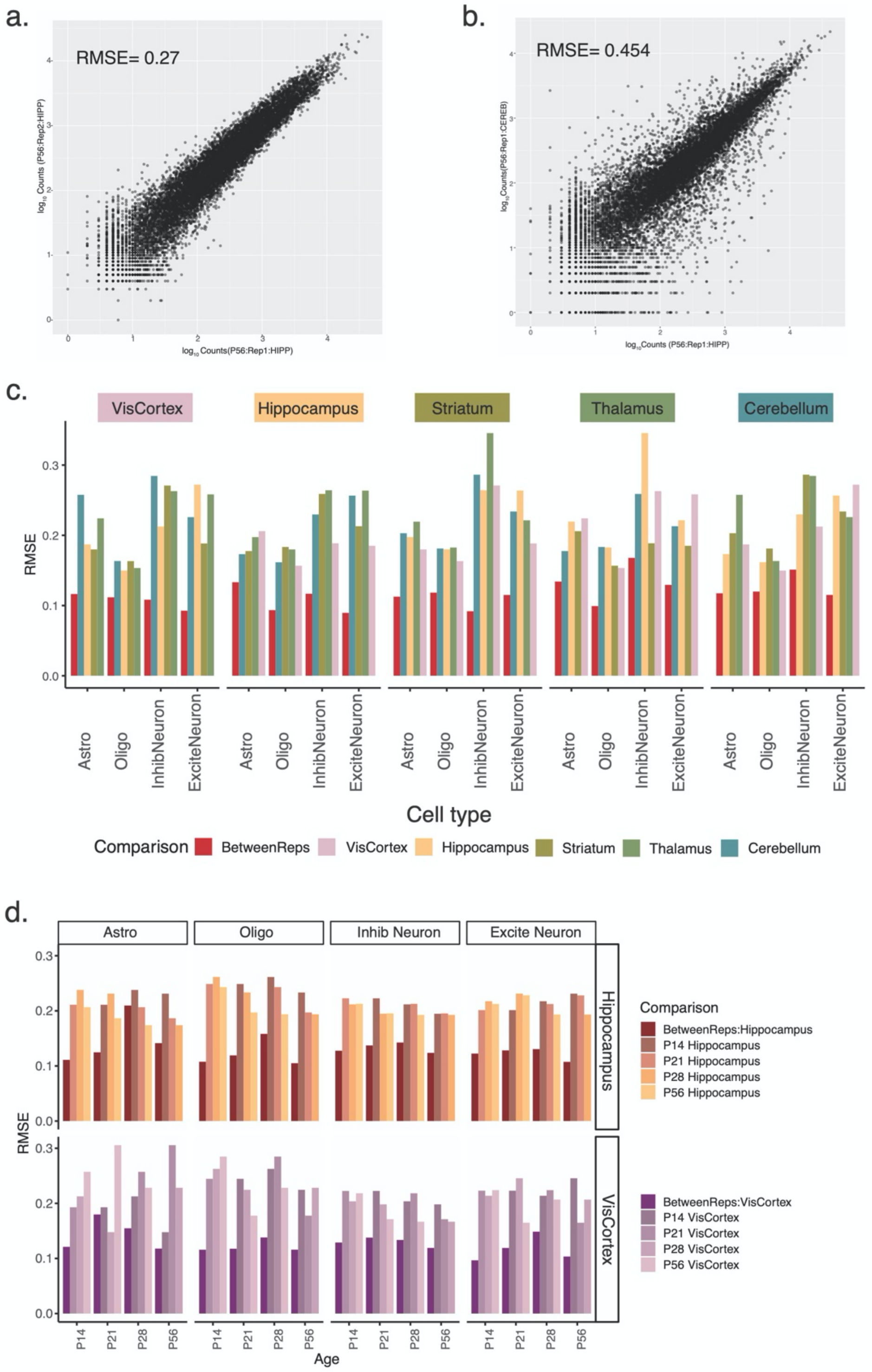

Supplementary Fig 8: Within-sample variability (a) Scatter plot of long-read gene expression represented as $\log_{10}$ Counts for two replicates (Rep1: x-axis, Rep2: y-axis) of P56 Hippocampus. Root mean square error (RMSE) denoting dispersion from y = x also indicated. **(b)** Same as in a but with P56 Rep1 Cerebellum on y-axis. **(c)** *left* Barplot showing the RMSE between replicates in astrocytes in VisCortex (red), and between VisCortex and other brain regions (teal: Cerebellum, yellow: Hippocampus, olive: Striatum, green: Thalamus). Panels on the right show the same but for other brain regions. **(d)** Same as in (c) but showing the RMSE between replicates versus between ages for Hippocampus (*top*) and for VisCortex (*bottom*)

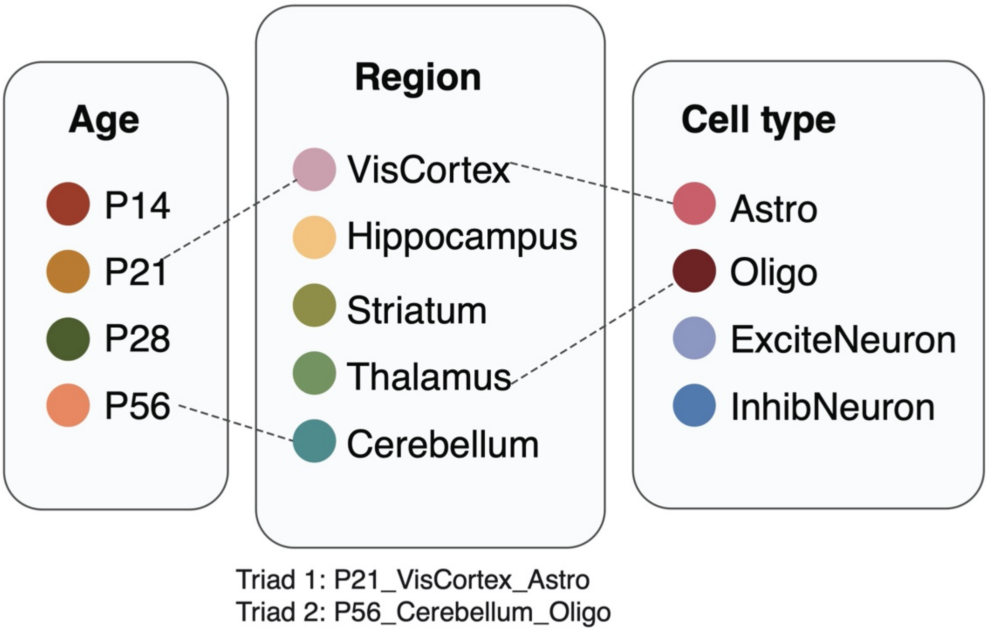

Supplementary Fig 9: Definition of a triad. A triad can be defined by the age, region, and major cell type of origin, with counts being averaged over replicates. This results in 44 triads given sample constraints.

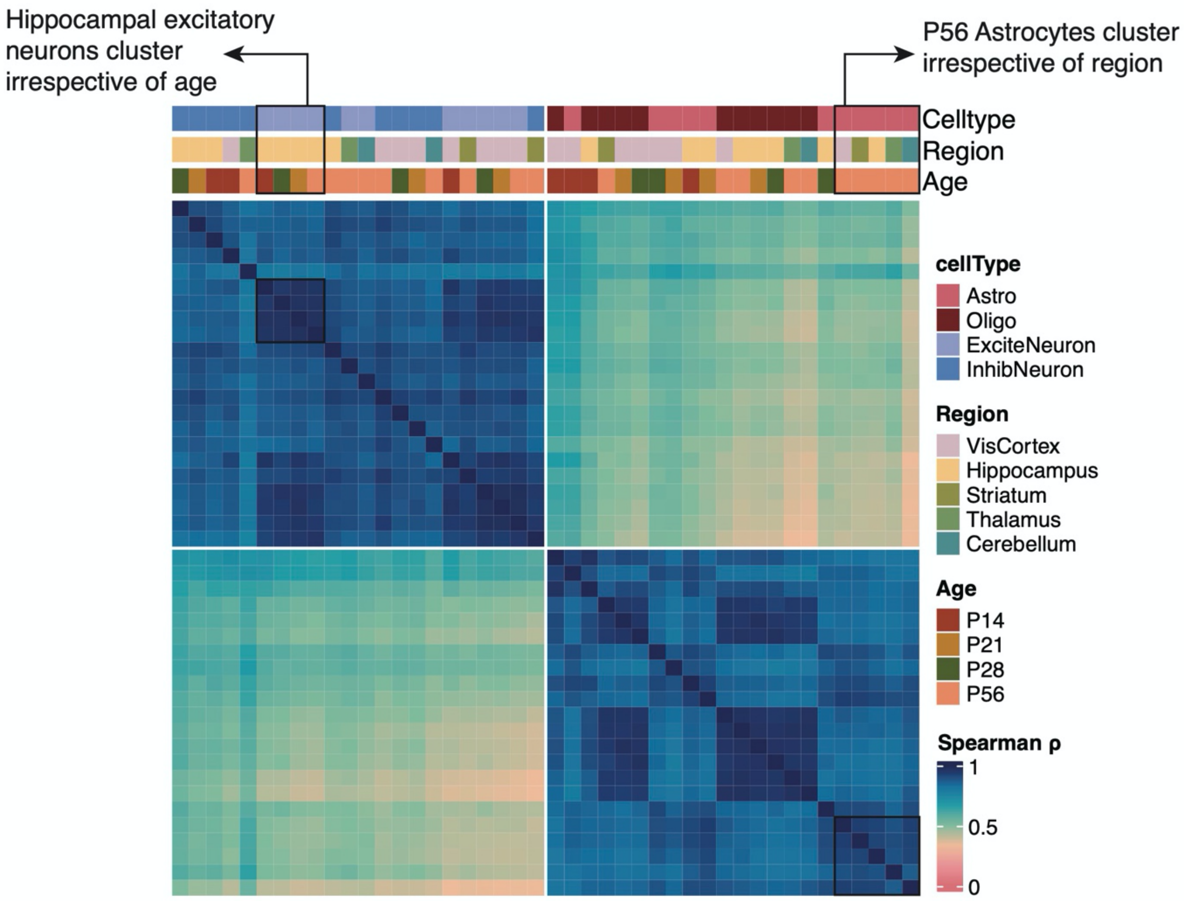

Supplementary Fig 10: Correlation of triad PSIs. Heatmap representing the spearman correlation values of triad exon Ψ values across all measurable exons. Color of tile represents the spearman ρ Annotation bars represent the major cell type (top), brain region (middle), and age (bottom) contributing to each of the 44 triads. Black outlines indicate astrocytes at P56 which have clustered together regardless of brain region, and excitatory neurons in the hippocampus which have clustered together regardless of timepoint.

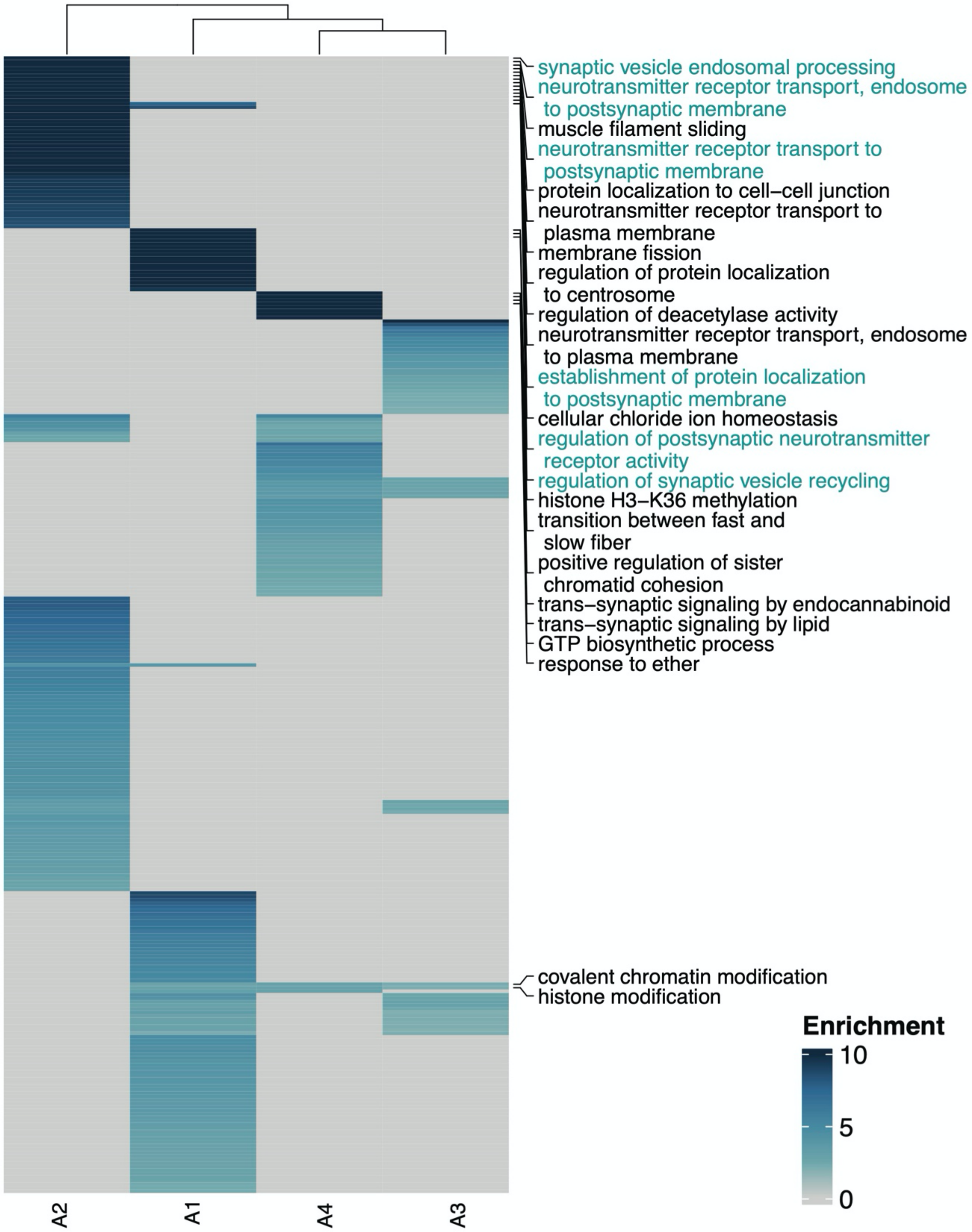

Supplementary Fig 11: Gene ontology of hVEx groups. Heatmap of the biological process gene ontology (GO-BP) enrichment terms for each of the four categories of highly variable exons (hVEx). Color of tile indicates the level of enrichment in categories compared to the background. GO terms with very high enrichment observed in a single category, or medium enrichment observed across multiple categories are reported.

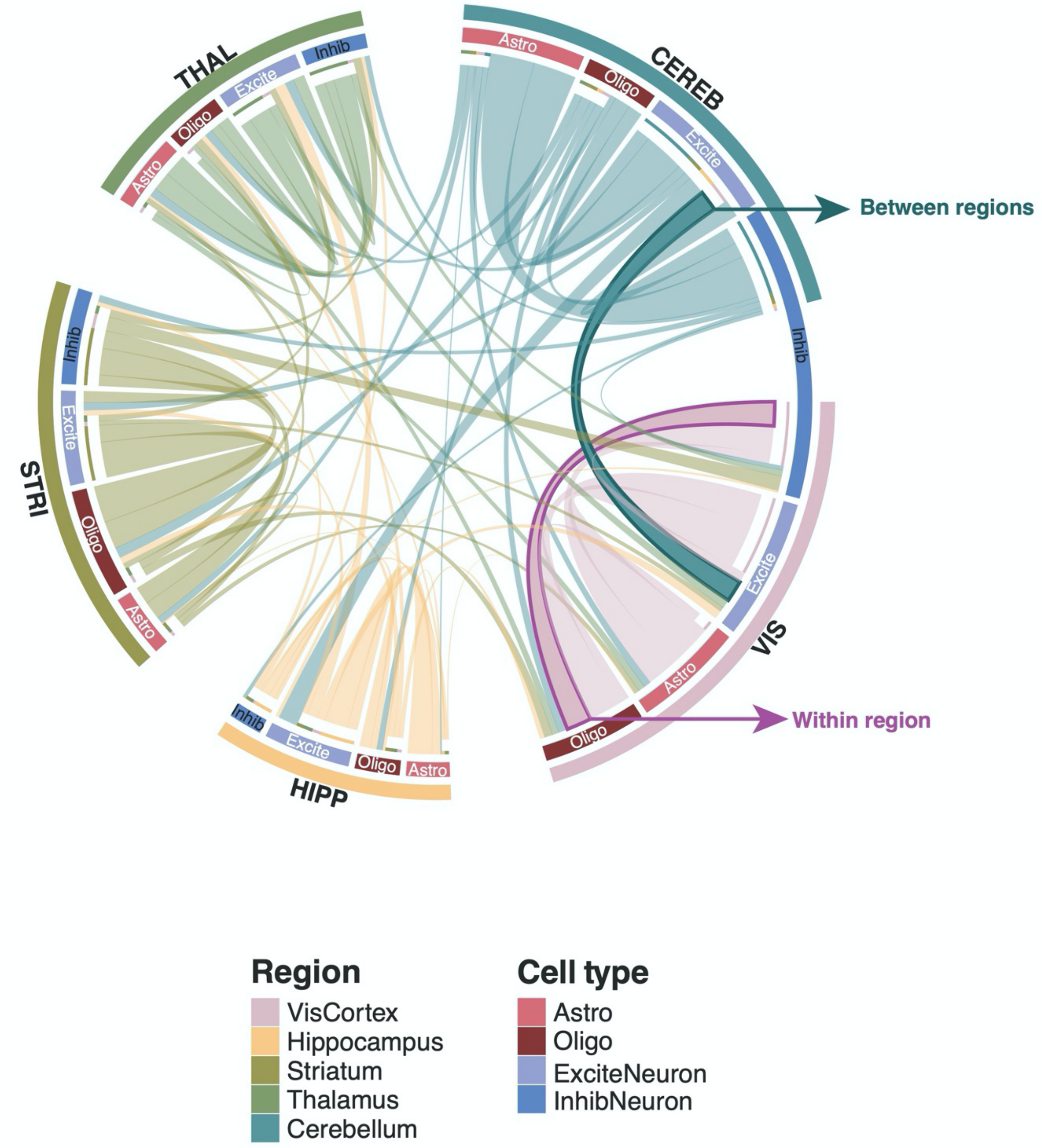

Supplementary Fig 12: Circos plot of highly variable exons (hVEx) changing inclusion at P56. Outer concentric ring: brain region; inner concentric ring: cell types. At constant age (P56), triad is defined by brain region and cell type. Triads connections indicate that hVExs are present in this comparison. Connection thickness indicates number of hVExs detected in comparison. Connection color indicates brain region of origin but cross-brain region-comparison colors are random.

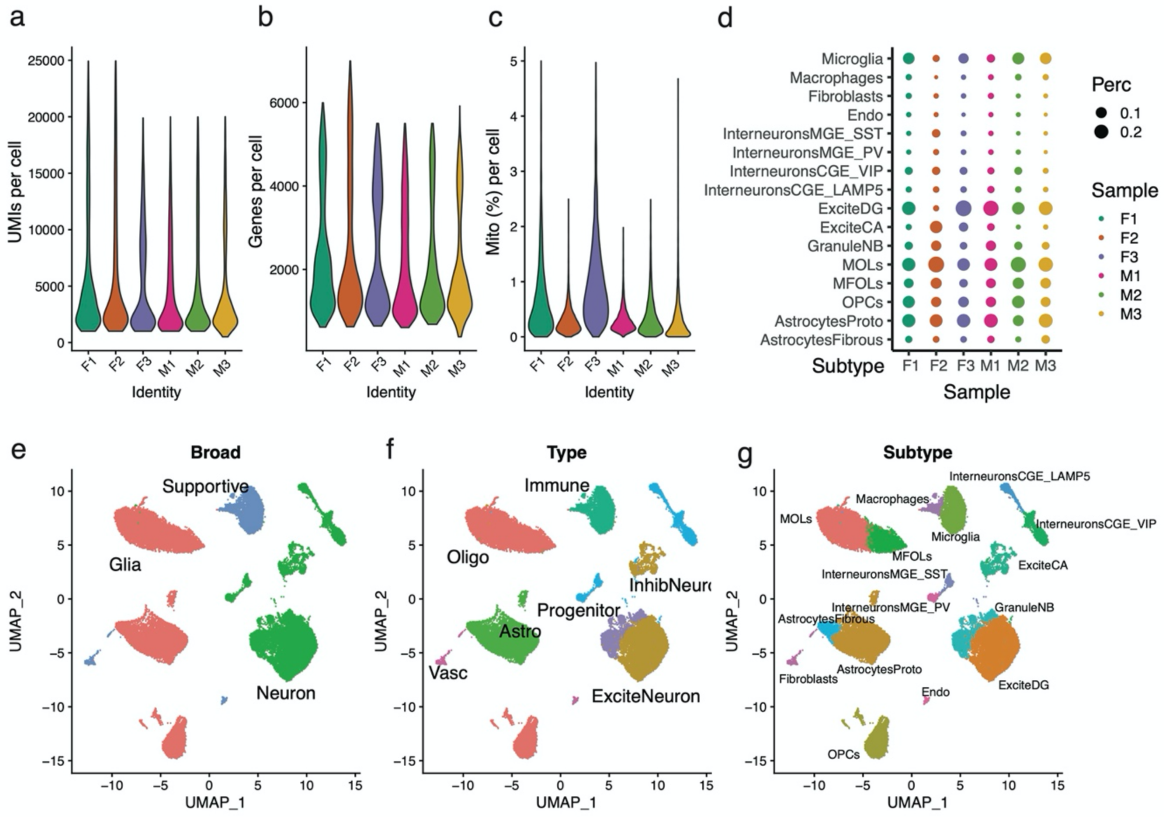

Supplementary Fig 13: Processing of short-read snRNA human hippocampal data. (a) Violin plots of the UMIs per nucleus for each of the six hippocampal samples indicated on the x-axis. (b) Same as in (a) but showing a distribution of genes per cell nucleus (c) Same as in (a) but showing the distribution of the percentage of mitochrondrial reads sequenced per cell nucleus. (d) A dot plot indicating the percentage of nuclei per sample (x-axis) belonging to a cell subtype (y-axis). Size of dot indicates percentage while color of dot indicates sample. (e) UMAP embedding of all nuclei from the six samples clustered together after controlling for batch effects Each point represents a single cell nucleus and color of cluster denotes the broad cell type i.e., green: neurons, red: glia, and blue: supportive cells. (f) Same UMAP as in (e) but colored by cell type. (g) Same UMAP as in (e) but colored by cell subtype.

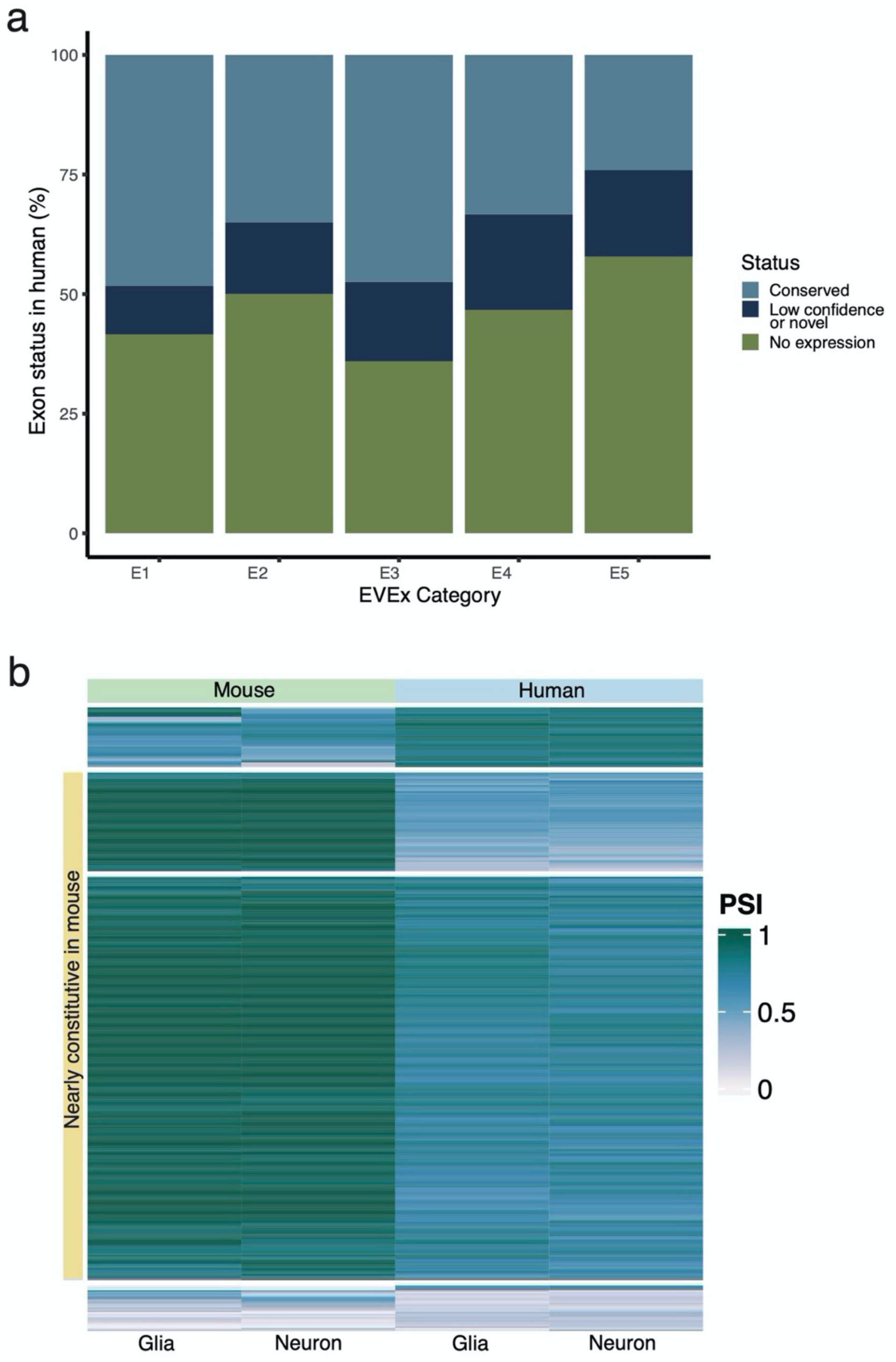

Supplementary Fig 14: Exon conservation between mouse and human (a) Barplots for each group of extremely variable exons (EVEx, E1- E5) indicated on the x-axis with the percentage of exons on the y-axis. Bars are split according to whether the exon has an ortholog and has a calculable Ψ in human data (light blue), does not have an ortholog in human mapped with high-confidence (navy), or has an ortholog but is too lowly expressed to calculate a Ψ value. **(b)** Heatmap of Ψ values in glia and neurons in mouse (left side) versus humans (right size) for exons that are lowly variable in humans. Yellow annotation bar on the left indicates exons in which Ψ values are close to 1 in mouse, indicating that the exons are nearly constitutively included in mouse.

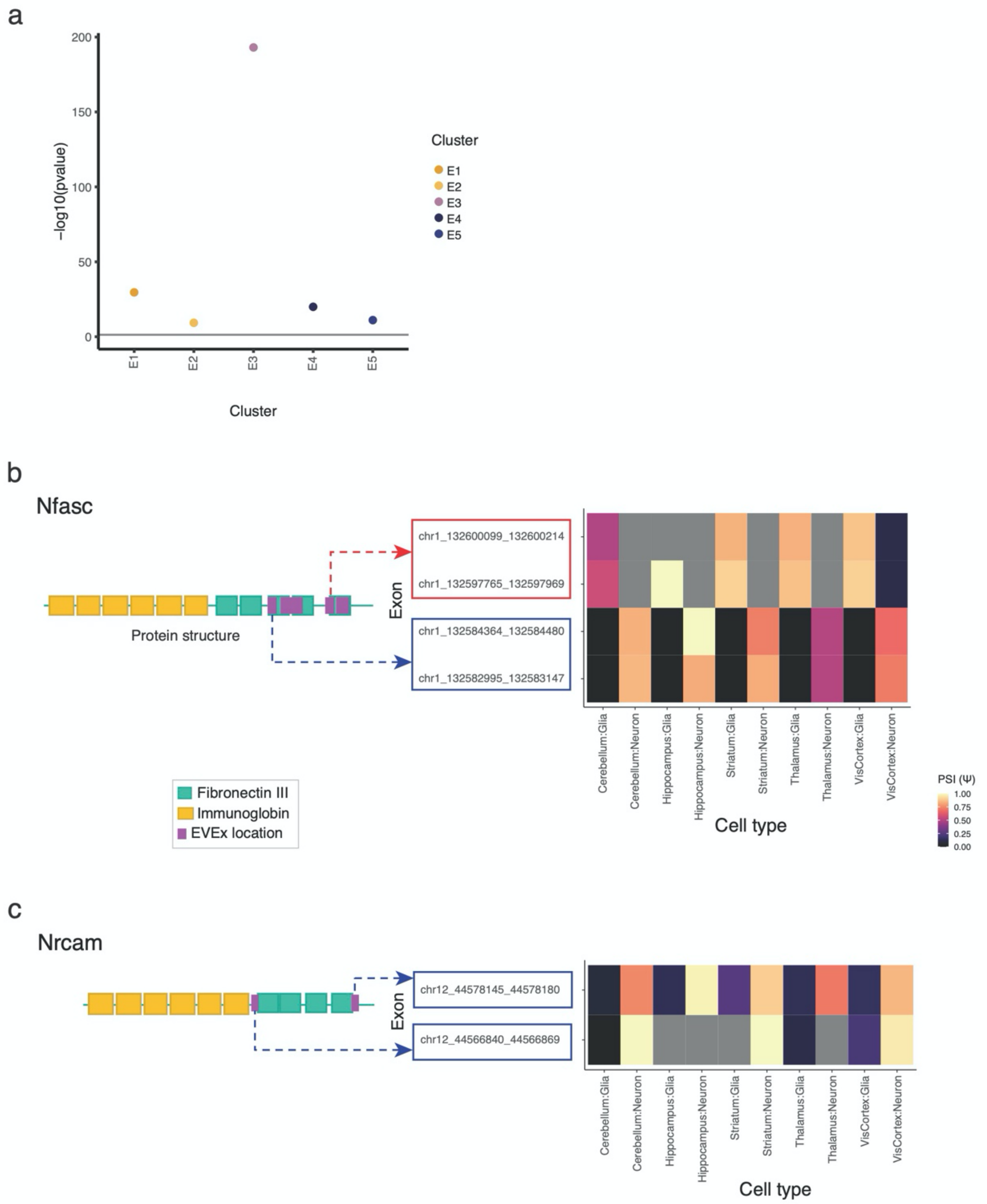

Supplementary Fig 15: Domain architecture of extremely variable exons (EVEx). **(a)** Dotplot showing the $\log_{10}$ p-value obtained using the χ^2 test of different EVEx categories (x-axis) having a higher prevalence of the inter-domain linker than other superfamilies. No correction for multiple testing was performed **(b)** *Left* Domain information for the NFASC protein with immunoglobin (IG)-like domains in yellow followed by Fibronectin Type III (Fn3) domains in teal. Highlighted parts (purple) denote EVEx with cell type specificity. *Right* Heatmap of exon inclusion (PSI) values for the four EVEx identified for the *Nfasc* gene. Top two exons show a clear preference for glial inclusion while bottom two are highly included in neurons for all five brain regions at P56. Grey tiles indicate NA values. **(c)** Same as in (a) but for the gene *Nrcam* where both exons are highly included in neurons.

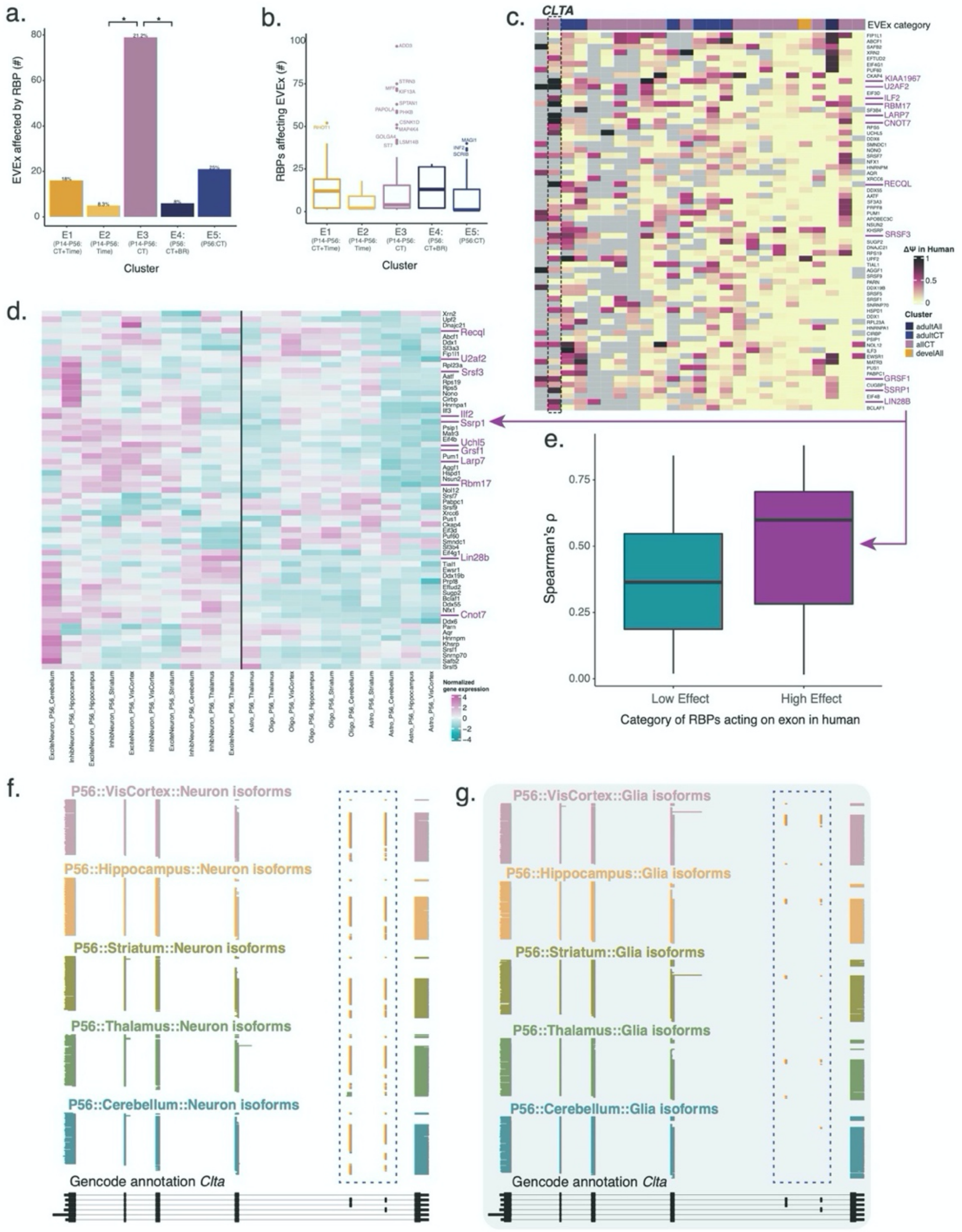

Supplementary Fig 16: Effect of RNA binding protein (RBP) expression on cell-type specific splicing. (a) Barplot of number of exons significantly affected by RBP expression. X-axis and color of bar indicates EVEx category. **(b)** Boxplot showing the number of RBPs that each exon is affected by. X-axis and color of box indicates 5 EVEx categories with N = 16,5,79,6,21 exons respectively. Outlier points indicate RBP gene names. **(c)** Heatmap showing $|\Delta\Psi|$ of exons identified as EVEx in human cell line data (category indicated on the top). RBPs identified as having a high effect on the exon inclusion of an alternative exon in *CLTA* highlighted in purple. **(d)** Heatmap of the mouse RBP ortholog gene expression in mouse cell types indicated on the x-axis. **(e)** Boxplot of correlation in gene expression of RBPs in mouse with *Clta* Ψ values for cell types shown in (d). Blue indicates RBPs with low effect (n=26) on CLTA as identified in (b) and purple indicates RBPs with high effect (n=11). **(f)** Cluster-resolved single-cell long reads for *Clta* gene in neurons. Each line is a single cDNA molecule. Orange exons: alternative exons. Colors indicate region of origin. Bottom black track: Gencode annotation. **(g)** Same as in (f) but for glial cell types. For boxplots **b,e**, center line, median; box limits, upper and lower quartiles; and whiskers, 1.5× interquartile range.

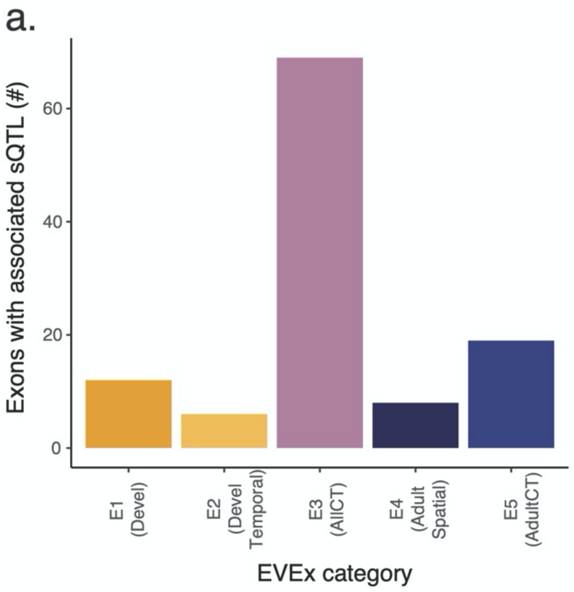

Supplementary Fig 17: Querying sQTLs associated with exons. (a) Barplot denoting number of exons with associated splicing QTL (sQTL) queried from the GWAS catalog. X-axis represents the EVEx category E1-E5 as defined in Fig 3d. CT: Cell type

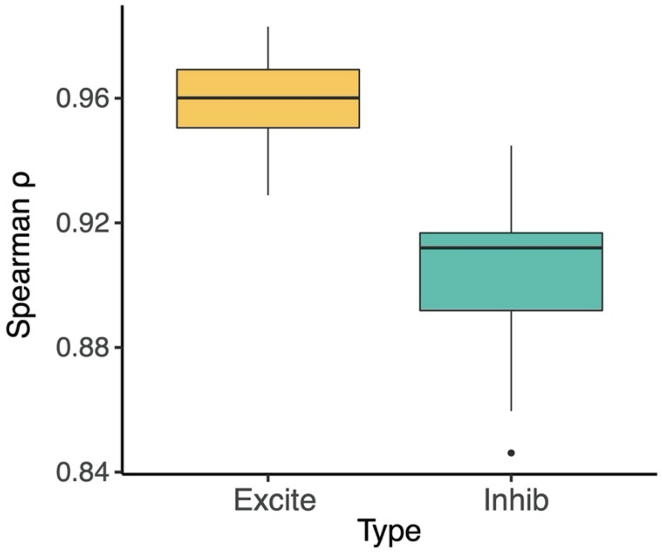

Supplementary Fig 18: Correlation within excitatory and inhibitory neurons. Boxplot of the pairwise spearman correlation (ρ) values of the exon inclusion values across all subtypes of excitatory neurons (n=30) in all developmental timepoints (yellow), and same for inhibitory neurons (n = 8, teal). Center line, median; box limits, upper and lower quartiles; and whiskers, 1.5× interquartile range.

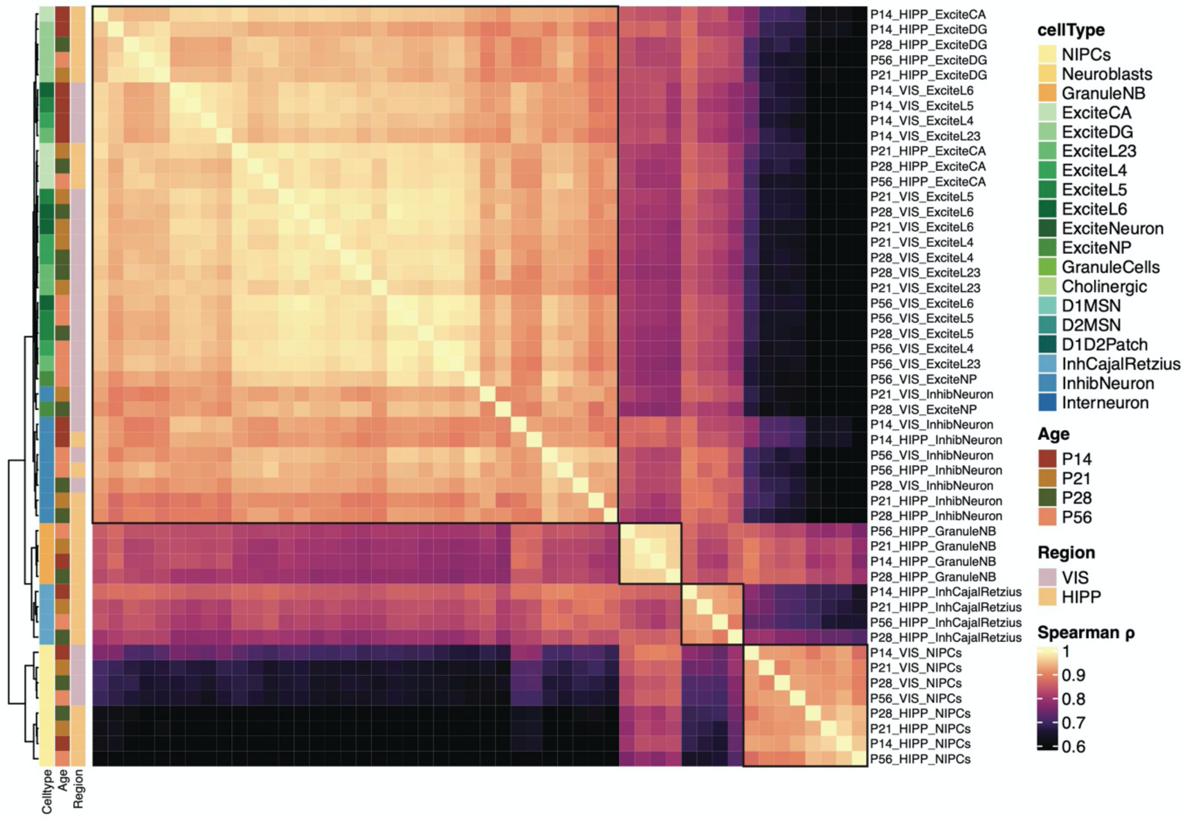

Supplementary Fig 19: Correlation of neuronal subtype Ψ. Heatmap of the pairwise spearman correlation (ρ) values of the exon Ψ across all subtypes of excitatory and inhibitory neurons in HIPP and VIS considered together. Annotation bars on left indicate the cell subtype (left-most), age (middle), and region (right-most) of origin for each subtype considered. Black boxes outline the major splits in the dendrogram.

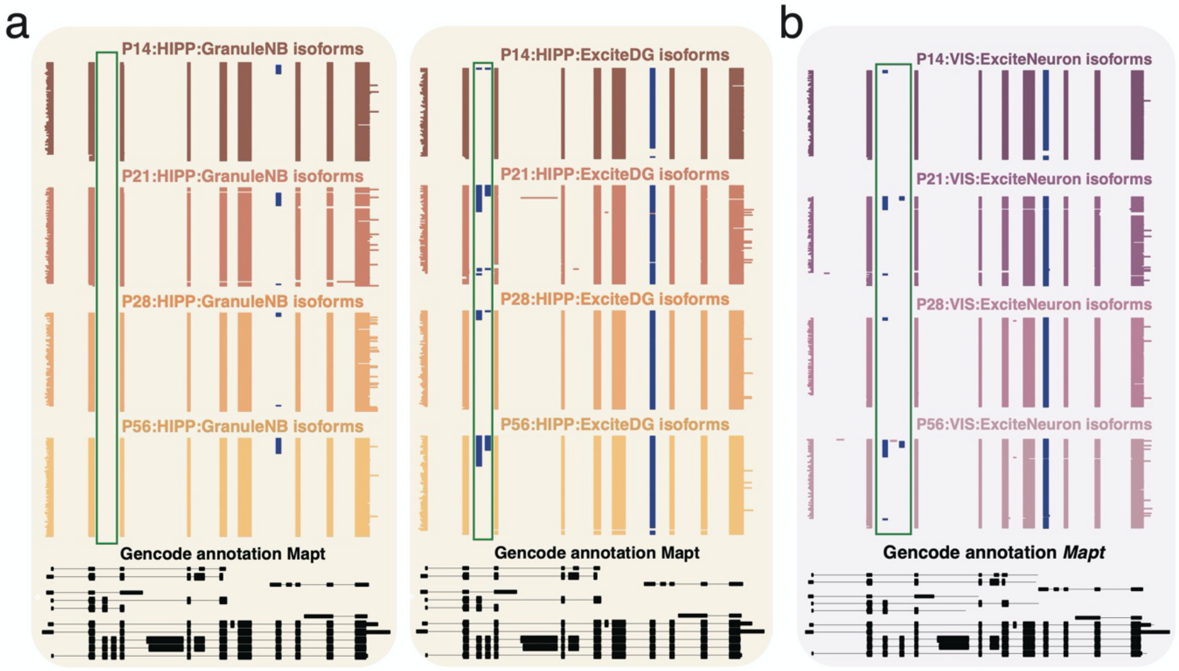

Supplementary Fig 20: Developmental isoform expression of *Mapt* showing developmental isoform variability. (a) ScisorWiz plot showing the isoforms for the gene *Mapt* for granule neuroblasts (left) and dentate gyrus excitatory neurons (right) in the hippocampus colored and split by age. Each line indicates a unique cDNA molecule, with clustered chunks denoting exons. Alternative exons denoted in blue. The first two alternative exons are outlined with a green box. Black lines on the bottom indicates GENCODE annotated transcripts. **(b)** Plot with a similar structure as in (a) but for visual cortex excitatory neurons.

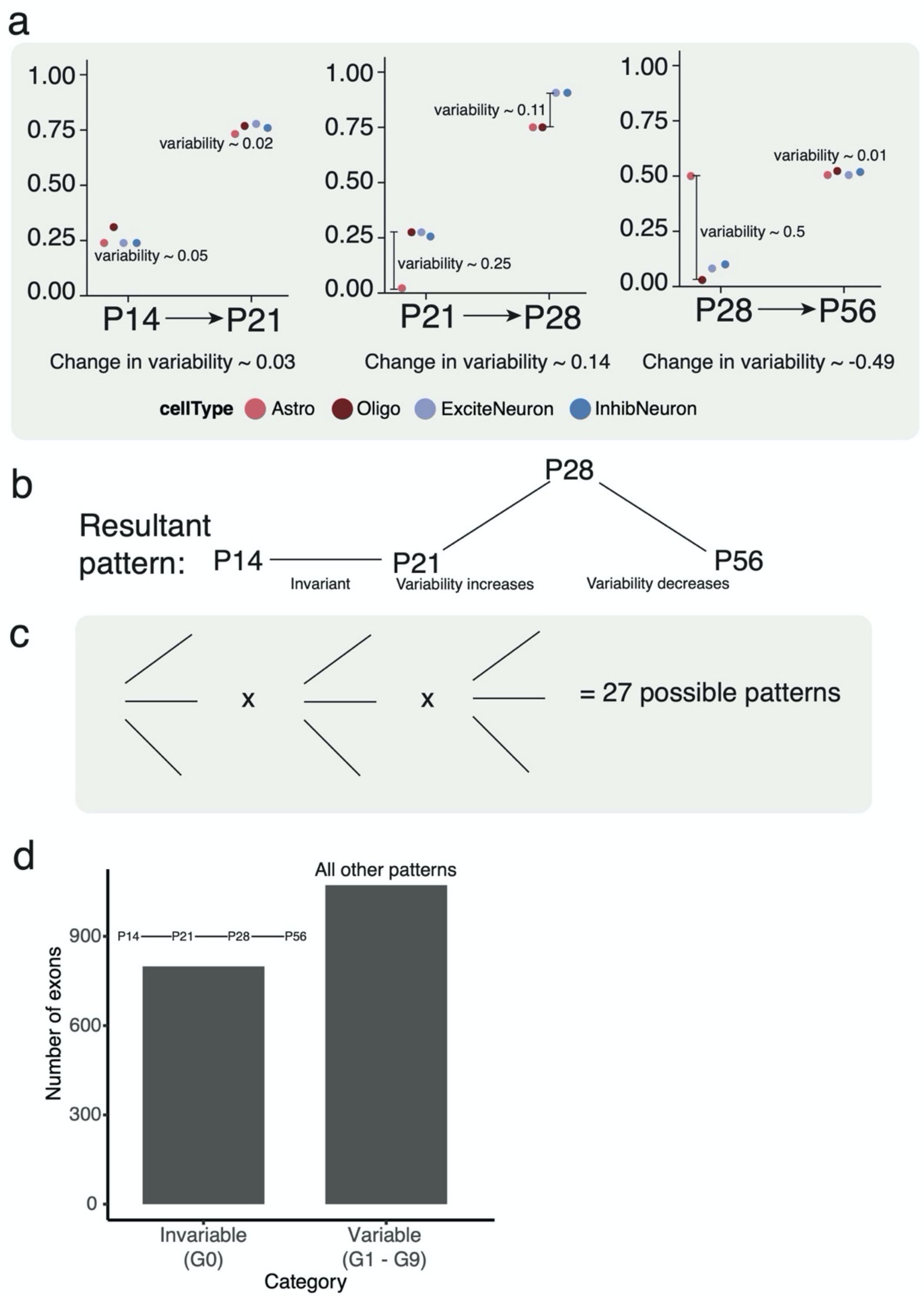

Supplementary Fig 21: Defining patterns of developmental variability. (a) Schematic representing the change in variability for major cell types in each of the three developmental transitions indicated on the x-axis. X-axis indicates time point and y-axis shows the Ψ value. **(b)** The pattern of variability resulting from the hypothetical schematic shown in (a). **(c)** Given that the variability can go up, down, or remain fairly constant, and there are three developmental transitions under considerations, 27 patterns of variability are theoretically possible. **(d)** Under the definition that the variability ≤ 0.1 for each of the three transitions, a barplot showing the number of exons defined as invariable versus variable. The variable exons are then used to obtain the 9 developmental patterns of variability.

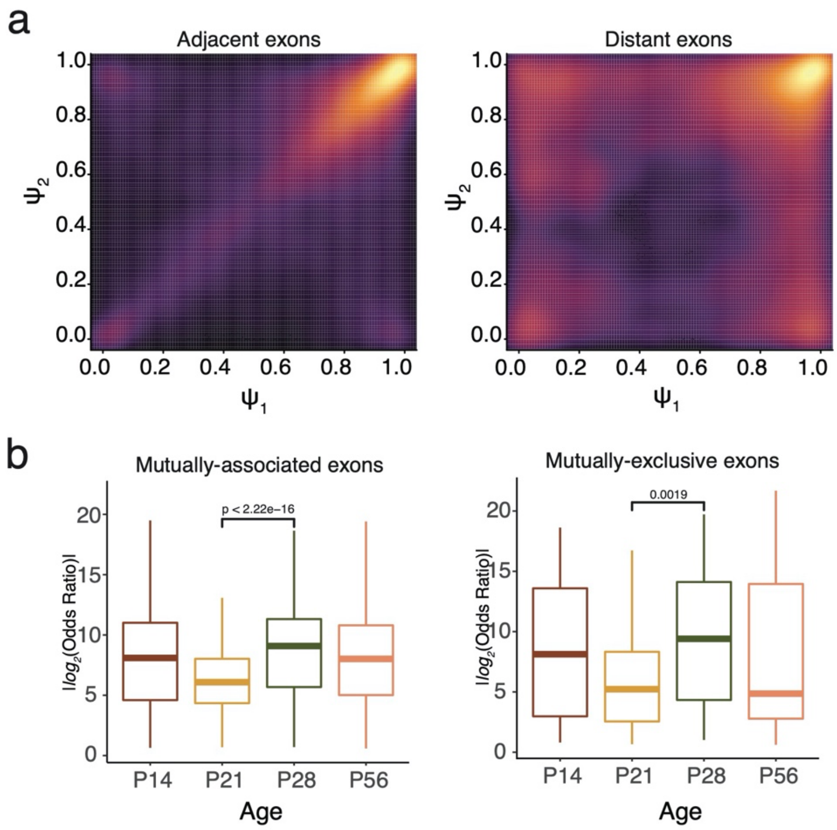

Supplementary Fig 22: Inclusion patterns for pairs of exons. (a) A smoothed scatterplot showing the individual Ψ of pairs of adjacent exons (*left*) which are often included or skipped together, and distant exons (*right*) which can be mutually exclusive. Black/ purple indicates low enrichments of points while yellow indicates high enrichment. **(b)** Boxplots of the effect size of coordination for pairs of exons. Y axis show the absolute value of the $\log_2$ odds ratio for mutually associated exons (*left,* n = 419, 1554, 518, 1281) and mutually exclusive exons (*right*, n = 80,96,85,107) for each of the developmental timepoints indicated on the x-axis. P28 has higher $|\log_2(\text{odds ratio})|$ than P21 (p-value $<2.2e^{-16}$ and p=0.0019 respectively) Higher absolute odds ratio indicates tighter coordination. P-values obtained from two-sided Wilcoxon rank sum test. Center line, median; box limits, upper and lower quartiles; and whiskers, 1.5× interquartile range.

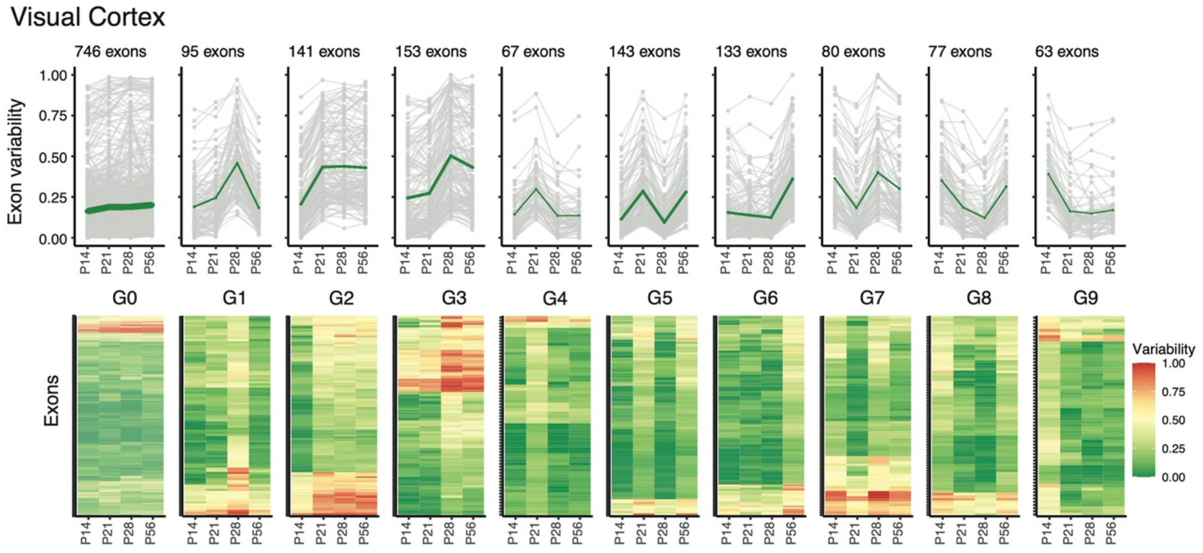

Supplementary Fig 23: Patterns of developmental variability in the mouse visual cortex. Line plots showing the variability value for exons in the invariable category (G0) and the nine patterns of variability identified (G1-G9) on the y-axis and the four timepoints on the x-axis. The number of exons in each category for visual cortex is indicated above. Green line connects the mean variability value per group and time point (*top*). Heatmap of the exon variability for each category (G0-G9) and each time point indicated on the x-axis (*bottom*)

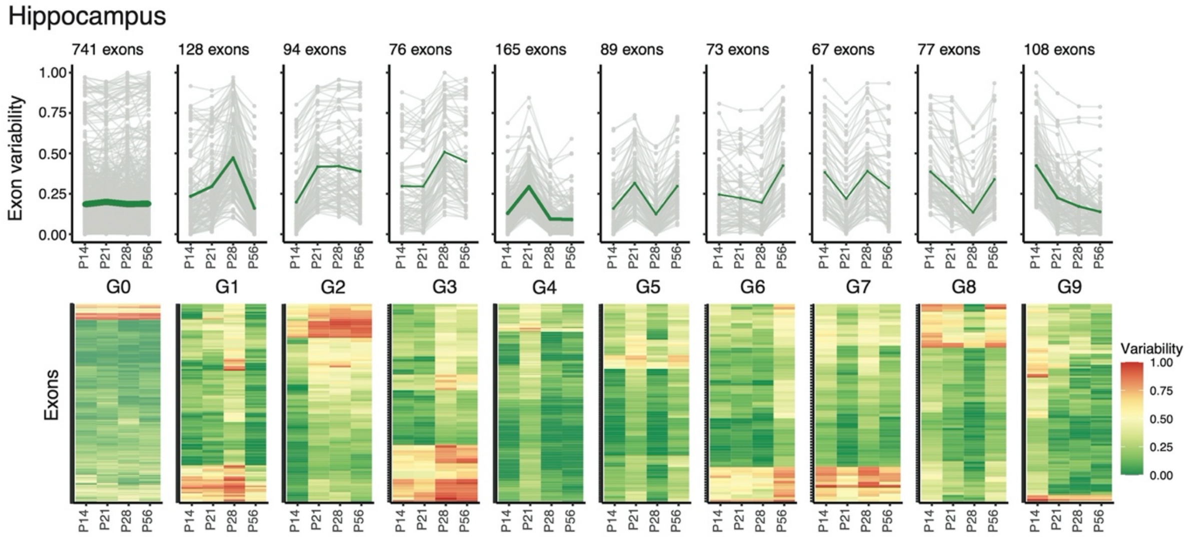

Supplementary Fig 24: Patterns of developmental variability in the mouse hippocampus. Line plots showing the variability value for exons in the invariable category (G0) and the nine patterns of variability identified (G1-G9) on the y-axis and the four timepoints on the x-axis. The number of exons in each category for hippocampus is indicated above. Green line connects the mean variability value per group and time point (*top*). Heatmap of the exon variability for each category (G0-G9) and each time point indicated on the x-axis (*bottom*)

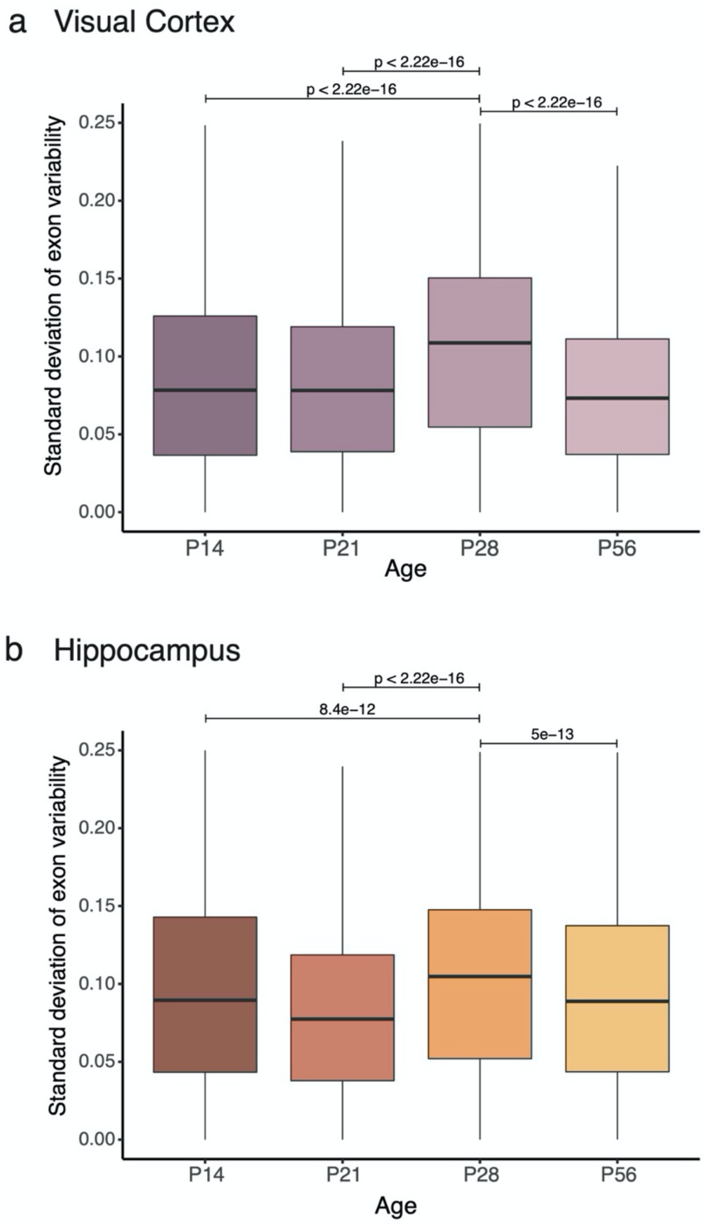

Supplementary Fig 25: P28 as a critical timepoint for exon variability between cell types (a) Boxplots showing the standard deviation of exon variability between the major cell types in the visual cortex on the y-axis, at each timepoint indicated on the x-axis (n = 2081, 3432, 2373, 5872 exons). **(b)** Same as in (a) but for hippocampus (n = 2309, 5174, 2146, 2550 exons). Center line, median; box limits, upper and lower quartiles; and whiskers, 1.5× interquartile range. P-values obtained using the two-sided Wilcoxon rank sum test, no correction for multiple testing was done.

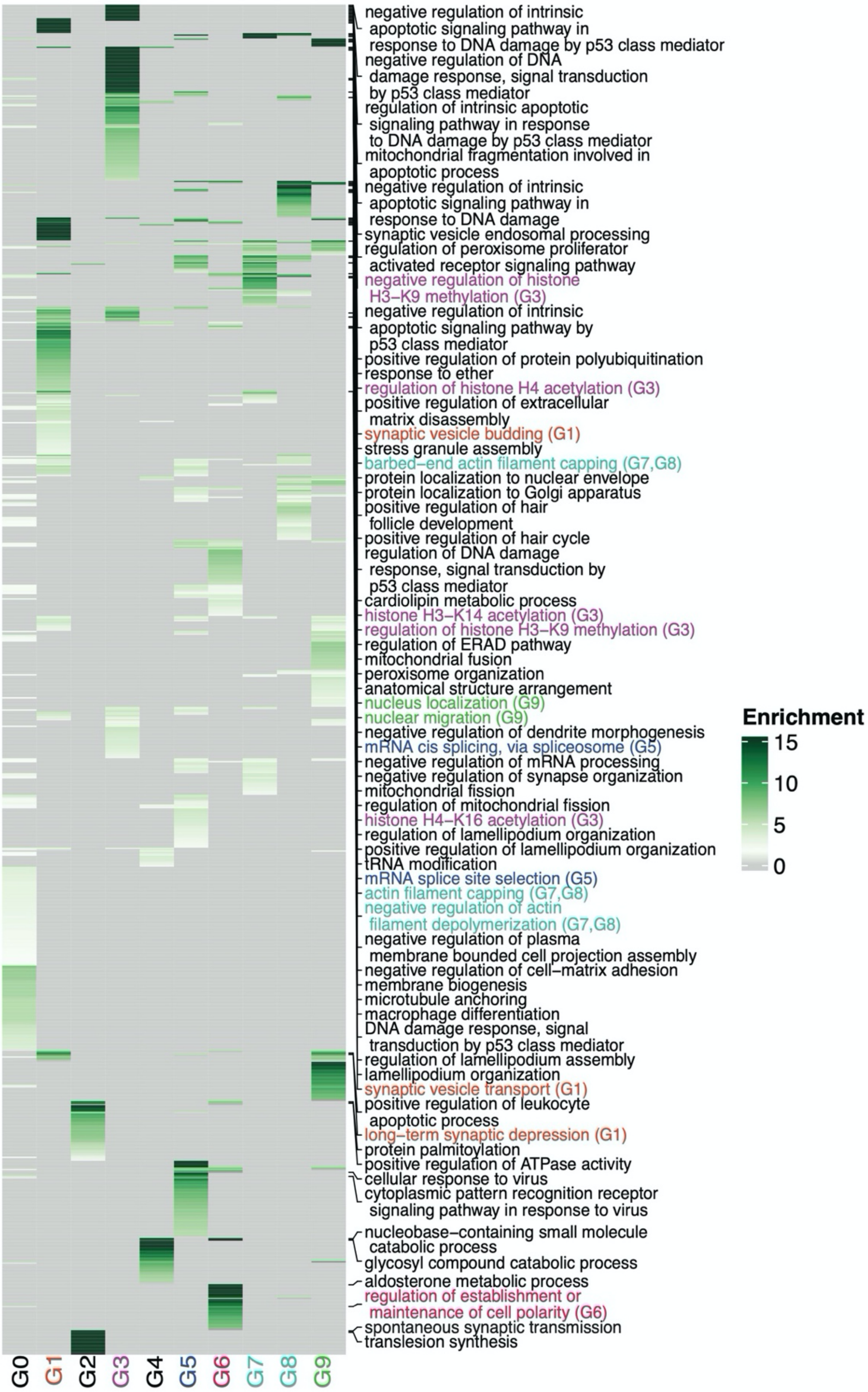

Supplementary Fig 26: Gene ontology enrichment for the developmental patterns of variability. Heatmap of the biological process gene ontology (GO-BP) enrichment terms for each of the nine variable and one invariable (G0) patterns of developmental variability of exon inclusion. Color of tile indicates the level of enrichment in categories compared to the background. GO terms with very high enrichment observed in a single category, or medium enrichment observed across multiple categories are reported. Some terms and the group they belong to are highlighted in colors to aid the reader.

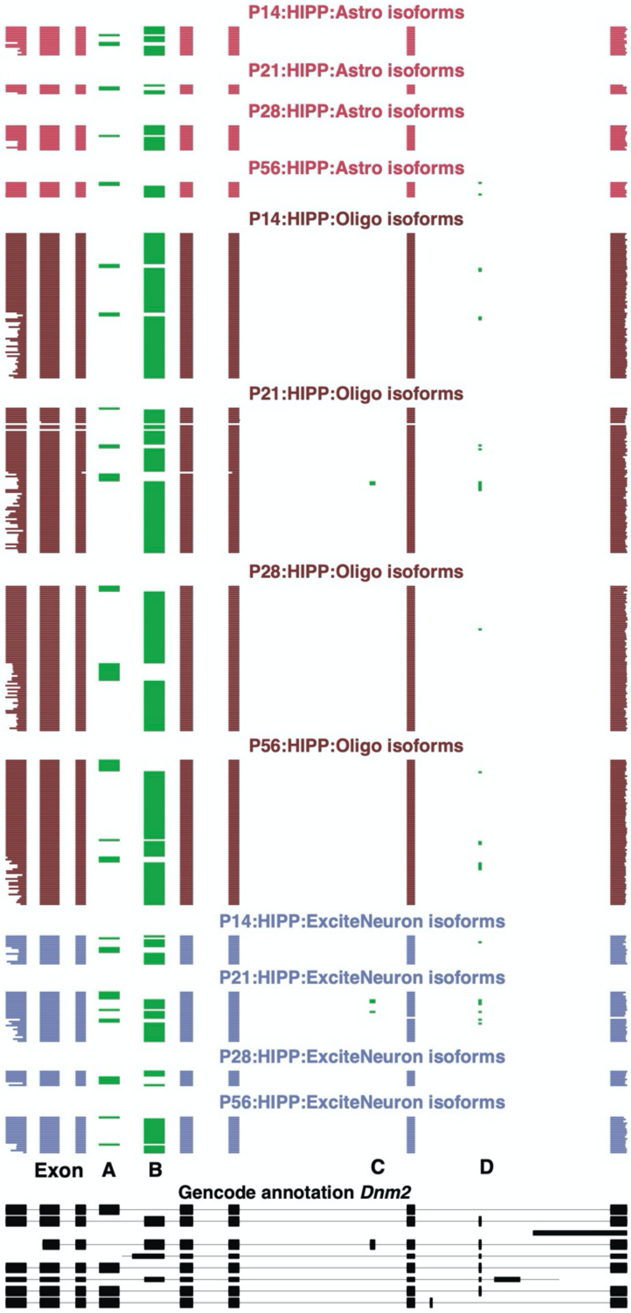

Supplementary Fig 27: Isoform expression of *Dnm2* in hippocampus. ScisorWiz plot showing the isoforms for the gene *Dnm2* for astrocytes (top, pink), separated by timepoint, oligodendrocytes (middle, maroon), and excitatory neurons (bottom, blue). Each line indicates a unique cDNA molecule, with clustered chunks denoting exons. Alternative exons are colored in green and are represented on the bottom. Black lines on the bottom indicates GENCODE annotated transcripts. Compare to Fig 6d-e

Supplementary Notes 1-7

Supplementary note 1: Considerations of varying cell numbers in the short-read analysis

The cerebellum is a generally observed to be challenging to dissociate, in part because of the robust arborization and connectivity of Purkinje cell neurons. Moreover, the cell type complexity within dissociated samples is lower since the great majority of cerebellar cells in the tissue are excitatory granule cells. The number of cells recovered per experiment with cerebellum is also generally low, with the state of the art yielding a maximum of 5000 cells per experiment even with sophisticated protocols utilizing laser capture microscropy and ophthalmic microscalpels (e.g., https://www.nature.com/articles/s41586-021-03220-z#Sec7, https://www.ncbi.nlm.nih.gov/pmc/articles/PMC8338761).

To ensure consistency of protocol across all samples and to retain biological replicates (P56 HIPP, STRI, and CEREB came from the same animals), we chose to proceed with the lower number of cells at the cost of not capturing rarer cerebellar cell types.

- We first performed rigorous QC by manually validating per-sample statistics i.e., total reads sequenced, intronic, intergenic, antisense alignments, and per-cell statistics, i.e., genes, UMIs, mitochondrial percentage. This was followed by stringent filtering based on per-sample cutoffs. Finally, we categorized cells into cell types based on marker gene expression based on the literature. Next, we integrated replicates using centroid correction on clusters with Harmony along either a developmental or spatial axis. This provided a logical check to ensure that our recovered cell types were consistently assigned across replicates, as well as regressed out potential batch effects from UMI counts, mitochondrial reads etc.
- Thus, using tools and best practices vetted by the single-cell community, we have taken into account technical differences that arise during sample preparation and short-read sequencing

Supplementary note 2: Considerations of varying cell numbers in the long-read analysis

- Number of cells recovered: The number of cells and cell types recovered from long reads is reliant on the barcodes recovered from the short read analysis. Therefore, as mentioned, rare cell sub types and their isoform profiles cannot be assessed. However, conclusions about abundant cell types can be made from the recovered cells in our experiments.
- Number of UMIs recovered: The number of UMIs recovered per sample is a consequence of long read sequencing depth and cells per sample. P56 Rep2 CEREB was sequenced 3x as much as Rep1 (**Table S3**). This resulted in ~3x reads / cell in Rep2 versus Rep1 on average. However, a lot of these were expectedly duplicated UMIs (**Fig S3a**). Upon filtering and retaining only one UMI per gene, we still see a higher UMI/cell distribution, but not drastically so (**Fig S3c**).
- Number of UMIs per gene: Upon examining the number of UMIs recovered per gene, we find that the numbers are consistent between replicates (**Fig S3b**). This means that difference in sequencing depth affects the exact genes being recovered between replicates, however, the number of UMIs per commonly expressed gene are similar between replicates, and in fact across all samples, and therefore comparisons can be made for these genes.
- Read truncation: Due to variability in library fragment size and sequencing runs, some transcripts were truncated and full transcripts not always recovered. However, we made use of IsoQuant, which has been benchmarked against other tools and has high specificity in recovering and assigning truncated reads to annotated transcripts. Additionally, we made use of exon PSI values for exons, and TSS and PolyA site assignment for reads that were only complete on one end, ensuring that we made conclusions based on moderate to highly expressed transcript elements.

- Estimation of splicing differences: As stated above, the number of UMIs per gene is broadly consistent between all samples after filtering out duplicates etc. All our analysis for exons is performed using PSI (percent spliced in) values, and for isoforms with PI (percent isoform) values. In cases where UMIs per gene falls below the threshold of 10, the PSI or PI value is reported as NA. This means that we only make comparisons between samples when the gene is sufficiently and commonly expressed, and we are confident in the assignment of a percentage value. Additionally, we average the values across replicates per sample. This allows for greater confidence in every percentage value.
- Bootstrapping and correlation to estimate variability between replicates and samples: We investigated the variability between replicates of the same samples by examining the correlation of Ψ values as well as performing downsampling experiments (Methods). This revealed that despite some within-sample variability between replicates in single-cell data, splicing differences were broadly replicable and between-sample comparisons showed comparatively very high differences in splicing.

To summarize, biases in long-read analysis caused by variability between samples were not an issue after correcting for UMI duplication, imposing minimum read thresholds, and normalizing for read depth per gene by employing percentage values.

Potential issues:

- Non-capturable genes: In some cases, comparisons between samples just cannot be made because the UMIs recovered for a gene are low. In this case, we report the values as NA but we cannot assess how that biases our results.
- Noisy data: Since droplet-based microfluidics is a stochastic process and long-read sequencing is of lower throughput, it is possible that what we recover is not representative of true biology within every single cell. We control for this by combining reads within a cell type, but again, this is harder to do for lowly expressed genes than for highly expressed genes.

Supplementary Note 3: Long read methodology and statistics

Oxford Nanopore Technology (ONT) and PacBio HiFi sequencing yielded 250×10^6 and 38×10^6 barcoded long reads respectively for 395 cell clusters (e.g., P56:Thalamus:Replicate1:OPCs) obtained from the short-read analysis pipeline (Methods, **Table S2-3**). Recent transcript-discovery software (IsoQuant)[48,49] was run on high accuracy single-cell PacBio reads using default PacBio parameters on an aggregate of all barcoded PacBio reads with GENCODE v21 as the reference annotation. IsoQuant (v2.3.0) identified novel splice sites, enhancing the GENCODE annotation by 22.1% (40,184 transcripts). No further analysis was done on this PacBio data.

Reads sequenced with Oxford Nanopore were aligned to the extended annotation. Additionally, the enhanced annotation gtf file was used on each of the ONT samples to correct incorrectly assigned splice sites in multi-exonic barcoded reads. Isoquant (v3.1) was run using default parameters for ONT data. Over 67.3% of mapped, barcoded ONT reads (SD=4.51%, **Table S2**) represented multi-exonic transcripts with trustworthy splice sites. The other ~30% of ONT transcripts were completely novel, i.e., both with respect to the GENCODE annotation and the novel transcripts identified through PacBio sequencing. We do not use the full-length completely novel transcripts discovered through ONT in our isoform-based analysis. Instead, we use the exons within these transcripts for our exon-based analysis.

We find that between 65.13% and 83.14% of the novel PacBio isoforms were recovered in the ONT data (**Fig S2b**). In past research, we have found that novel transcripts tend to be more lowly expressed than known ones. Therefore, the finding that not all PacBio-derived novel isoforms are also seen in ONT was expected. We also assessed their expression in individual samples as compared to the

annotated samples. We find that the novel PacBio isoforms are expressed 50-70% of the rate of annotated transcripts (Fig S2c). However, we find that 94.37% of all novel PacBio transcripts were validated in ONT by considering all samples together. This implies that novel isoforms are often lowly expressed across multiple cell types, brain regions or time points.

To avoid any bias introduced by an individual cell, we retained exactly one UMI per combination of gene and cell barcode, all samples exhibited QC metrics commensurate with their sequencing depth, and these reads were used to calculate isoform abundance per cell cluster (See **Supplementary note 2**, Fig S3).

Supplementary Note 4: Variability in full-length isoform expression along three axes

Within a fixed cell type, between 41.02% and 50.24% of genes had isoforms in distinct triangles. Bootstrapping experiments revealed that compared to the other cell types, excitatory neurons consistently had a higher number of genes with isoforms in distinct quadrants, implying that excitatory neurons display isoform regulation not recapitulated in other cell types (Supplemental Methods, Fig S4i). In addition to a third of genes showing multi-axial variability in most cell types, ~66% genes showed this hyper variability only in restricted cell types (Fig S5b). The gene *Rufy3*, which plays a role in neuronal polarity[49], axon growth[50], and synaptic plasticity[51], exemplified cell-type specific isoform variability along multiple axes. Three of the six annotated transcripts exhibited developmental variability in distinct cell types (progenitors, inhibitory neurons, and immune cells), two isoforms exhibited subtype and regional variability in astrocytes, while one isoform (Rufy3-207) was consistently found in the center-triangle (Fig S6).

Supplementary Note 5: Influence of RNA binding protein expression on cell-type specific splicing

We accessed ENCODE III data from two human cell lines: K562 and HepG2, wherein short hairpin RNA (shRNA) or CRISPR was used to deplete individual RNA binding proteins (RBPs), followed by RNA-seq of both, the knockdown (KD) and the control samples (https://pubmed.ncbi.nlm.nih.gov/32728046/, https://www.nature.com/articles/s41586-020-2077-3). A total of 263 RBPs were profiled in this study, and about a third were annotated as being involved in splicing regulation and RNA processing. We obtained exons that were significantly altered in their splicing profiles from all the RBP KD experiments. Although these were two adult human cell lines, given the fidelity of cell-type specific exon expression between species (Fig 3h), we expect that results will be somewhat consistent in mouse.
Upon intersecting the RBPs from both cell lines with our list of EVEx we found that 127 exons were significantly affected by the panel. This made up approximately 20% of all the EVEx, and the distribution per category from E1 – E5 ranged from 8-20% (Fig S16a). Notably, the cell-type specific categories had the most number and percent of exons being affected. This is unsurprising because the category E2 has a temporal (age-specific) component whereas category E4 has a brain regional component which cannot be recapitulated in the human cell line data.

Interestingly, we found that the relationship between RBP and EVEx was not one-to-one. In fact, starting with the aforementioned 127 EVEx (20% of all EVEx), we found that while 25% of these have a single significant RBP affecting its splicing percentage, while almost half of these 127 EVEx have 4 or more RBPs affecting them), indicating that EVEx regulation, including indirect effects, can be very complex. (Fig 3k).

This of course also varies by group (Fig S16b).

Considering the reverse direction, we can also determine how many exons each RBP strongly affects. We isolated exons from the ENCODE data which had a dPSI of at least 50% between KD and control in at least one cell line. Intersecting this set with EVEx yielded 25 exons. Visualizing the dPSI for all these exons revealed that each RBP acts on moderate to high levels on the splicing of multiple exons (Fig S16c). Furthermore, we also found that the expression levels of these RBP genes is extremely variable per cell type, brain region, and developmental timepoint, meaning that exon inclusion is affected to varying degrees in the mouse brain depending on which combination of RBPs and exons are expressed along which spatio-temporal axis (Fig S16d). We isolated an example of an exon acted upon by multiple RBPs which came from the gene *CLTA* in human. After separating RBPs with low (dPSI < 0.4) versus high effect (dPSI>= 0.4) on this exon, we found that the influence on exon inclusion is mouse is highly correlated with that in human (Fig S16e).

Surprisingly, we found very similar results for highly variable exons (hVEx). Here too, we found ~20% of all hVEx significantly being modulated by one or more RBPs. This indicates that it is not just the exons displaying very high variability, but also exons displaying moderate variability between cell types, brain regions, and timepoints that are influenced by RNA binding proteins.

Of course, a more systematic analysis of gene regulatory networks and splicing profiles would be needed to fully parse the regulation of cell-type specific isoform expression.

Supplementary Note 6: The potential effects of cell-type specific splicing on human diseases

We probed whether the splicing of EVEx has known ramifications in human diseases. 16% of EVEx have an associated published splicing QTL[70] (sQTL, Methods). This contrasts with 6% of the background set of constitutive and 21% of exons that display low variability in mouse (Fig 3k). The EVEx categories E3 and E5, i.e., of overall cell-type specificity have the highest number and percentage of associated sQTLs (Fig S17a). This implies that we have essentially identified a cell-type specific component of splicing QTLs which is difficult to query through tissue-level studies. Lastly, given the linkage disequilibrium of SNPs in the same haplotype block, we cross-referenced the genes within which these SNPs were located with the GWAS catalog. Most genes (81 of 114) had a significant association in a GWAS study. Of those, 49 had associations with neurological conditions such as bipolar disorder, schizophrenia, Alzheimer's disease, Parkinson's disease, anxiety and depression. The remainder had associations with cardiovascular disease, diabetes, etc., which could be more indirectly related to the brain. Alternatively, similar regulation may also affect non-brain tissues.

Supplementary Note 7: Cell-type and developmental expression of Dnm2

The ScisorWiz[77] plot shows *Dnm2* leveraging four alternative exons to produce 6 complex isoforms. In excitatory neurons at P14, two mutually exclusive exons (Exons A,B) define two major isoforms, the second of which dominates at P21 and P28. At P56 however, the inclusion of the first exon gets upregulated to again resemble the pattern at P14. However, two additional exons render the isoform landscape more complex, where Exon C is only visible in P56 excitatory neurons while Exon D starts upregulating its expression at P28 (Fig 6d). Exons A and B in glia at all timepoints resemble the splicing patterns of excitatory neurons at P21 and P28, but not at P14 and P56. In contrast to excitatory neurons, exon C is not seen in glia in the ages and region examined, while exon D is expressed uniformly starting at P28 (Fig 6e).
