# Supplementary material for: Single-cell long-read sequencing-based mapping reveals specialized splicing patterns in developing and adult mouse and human brain
Source: Nat Neurosci. 2024 Apr 9;27(6):1051–63. doi: 10.1038/s41593-024-01616-4 (PMC11156538; doi:10.1038/s41593-024-01616-4)
Supplement: Supplementary file 2 — Reporting Summary [file 41593_2024_1616_MOESM2_ESM.pdf]

Corresponding author(s): Hagen u Tilgner

Last updated by author(s): Feb 13, 2024

Reporting Summary

Nature Portfolio wishes to improve the reproducibility of the work that we publish. This form provides structure for consistency and transparency in reporting. For further information on Nature Portfolio policies, see our Editorial Policies and the Editorial Policy Checklist.

Statistics

For all statistical analyses, confirm that the following items are present in the figure legend, table legend, main text, or Methods section.

n/a	Confirmed	
☐	☒	The exact sample size (*n*) for each experimental group/condition, given as a discrete number and unit of measurement
☐	☒	A statement on whether measurements were taken from distinct samples or whether the same sample was measured repeatedly
☐	☒	The statistical test(s) used AND whether they are one- or two-sided<br>*Only common tests should be described solely by name; describe more complex techniques in the Methods section.*
☒	☐	A description of all covariates tested
☐	☒	A description of any assumptions or corrections, such as tests of normality and adjustment for multiple comparisons
☐	☒	A full description of the statistical parameters including central tendency (e.g. means) or other basic estimates (e.g. regression coefficient) AND variation (e.g. standard deviation) or associated estimates of uncertainty (e.g. confidence intervals)
☐	☒	For null hypothesis testing, the test statistic (e.g. *F*, *t*, *r*) with confidence intervals, effect sizes, degrees of freedom and *P* value noted<br>*Give P values as exact values whenever suitable.*
☒	☐	For Bayesian analysis, information on the choice of priors and Markov chain Monte Carlo settings
☒	☐	For hierarchical and complex designs, identification of the appropriate level for tests and full reporting of outcomes
☒	☐	Estimates of effect sizes (e.g. Cohen's *d*, Pearson's *r*), indicating how they were calculated

Our web collection on statistics for biologists contains articles on many of the points above.

Software and code

Policy information about availability of computer code

Data collection	Viable cells were selected using FlowJo version 10 software. Illumina reads were converted to fastq using bcl2fastq. PacBio long reads were basecalled using SMRT-Link (v 8.0.0.78867) and CCS (8.0.0.80529). ONT long reads were basecalled using Min KNOW Core (v 4.0.5), Bream (v6.0.10), and guppy (v4.0.ll) on the PromethlON machine.
Data analysis	Data analysis was done using a combination of open source publicly available code and custom code. Software used in this analysis included: STARlong (v2.7.0), minimap2 (v2.17-r943-dirty), scisorseqr (v0.1.9), lsoQuant (v2.3.0) for PacBio and lsoquant (v3.l) for ONT, cellranger (v3.l.0), Seurat (v 3.2.2 and v3.2.3), harmony (v0.1.0), Slingshot (vl.6), ensembledb (v2.26). Data visualization was performed using a ScisorWiz, ComplexHeatmap (v 2.13.1), clusterProfiler (v3.18.1),<br>The source code generated for this paper is publicly available at https://github.com/noush-joglekar/biccn_tilgner _scisorseq

For manuscripts utilizing custom algorithms or software that are central to the research but not yet described in published literature, software must be made available to editors and reviewers. We strongly encourage code deposition in a community repository (e.g. GitHub). See the Nature Portfolio guidelines for submitting code & software for further information.

Data

Policy information about availability of data

All manuscripts must include a data availability statement. This statement should provide the following information, where applicable:

- Accession codes, unique identifiers, or web links for publicly available datasets
- A description of any restrictions on data availability
- For clinical datasets or third party data, please ensure that the statement adheres to our policy

The summary of all mouse data used for this study is available on the Knowledge Brain Map at https://knowledge.brain-map.org/data/Z0GBA7V12N4J4NNSUHA/summary and all human data is available on https://knowledge.brain-map.org/data/ASP3B09DZ8PXDUYSHDH/summary. These pages contains links to raw and processed data hosted on the Neuroscience Multi-Omic data archive (NeMO) under the identifier dat-717krsa (https://assets.nemoarchive.org/dat-717krsa). All data supporting the findings of this study are provided within the paper and its supplementary information. Publicly available data was downloaded from APPRIS (https://apprisws.bioinfo.cnio.es/landing_page/), ENCODE (https://www.encodeproject.org/), GTEx (https://www.gtexportal.org/home/downloads/adult-gtex/qtl), and the GWAS catlog (https://www.ebi.ac.uk/gwas/docs/file-downloads). Source data for the main figures can be found at https://github.com/noush-joglekar/biccn_tilgner_scisorseq/tree/main/data

Research involving human participants, their data, or biological material

Policy information about studies with human participants or human data. See also policy information about sex, gender (identity/presentation), and sexual orientation and race, ethnicity and racism.

Reporting on sex and gender	6 human (3 Male, 3 Female) hippocampal tissue samples were acquired.
Reporting on race, ethnicity, or other socially relevant groupings	All subjects were unaffected controls with no pathological diagnosis, and died of accidental causes<br>Sex: 3 males, 3 females, age range: 28-40, Race: 2 Black and 4 White individuals
Population characteristics	Sex: 3 males, 3 females, age range: 28-40, Race: 2 Black and 4 White individuals. No pathological diagnosis
Recruitment	*Describe how participants were recruited. Outline any potential self-selection bias or other biases that may be present and how these are likely to impact results.*
Ethics oversight	Acquisition of human tissue samples was done through the NIH Neurobiobank and were compliant with research ethics stated by the NIH. All donors completed University of Maryland IRB-approved consent documents. They were informed via these consent documents that the donated tissue would be used for distribution to qualified researchers and that such distributions could be made at any time in the future. These consent documents also assured that the identity of the donor would remain unknown to any tissue recipients and those reviewing the results of their work.

Note that full information on the approval of the study protocol must also be provided in the manuscript.

Field-specific reporting

Please select the one below that is the best fit for your research. If you are not sure, read the appropriate sections before making your selection.

☒ Life sciences ☐ Behavioural & social sciences ☐ Ecological, evolutionary & environmental sciences

For a reference copy of the document with all sections, see nature.com/documents/nr-reporting-summary-flat.pdf

Life sciences study design

All studies must disclose on these points even when the disclosure is negative.

Sample size	No statistical methods were used to pre-determine sample sizes (e.g., cell number in a single-cell experiments) but we aimed for ~10000 single cells / experiment. These numbers are similar to those reported in previous publications (see PMIDs 31435019, 35256815). For the mouse experiment we had 11 samples (4 timepoints x 2 brain regions, 1 timepoint x 3 brain regions) with each sample having 2 biological replicates resulting in 22 experiments.<br>For the human samples there is more inter-individual variability so we had 1 brain region x 6 donors resulting in 6 samples
Data exclusions	No data was excluded from this study except for filtered cells after QC. Raw data have been deposited for all samples.
Replication	2 biological replicates were obtained for each sample from mouse, and 6 from human. QC and results were replicable, and these were confirmed by comparing gene, exon, and isoform expression profiles between replicates
Randomization	The null hypothesis of the study was that brain regions and developmental timepoints of wild type, healthy had no impact on alternative splicing patterns. No experimental manipulations of mice were performed. The study design was hence observational (known samples collected at different time points) and did not require randomization of experimental or control groups.
Blinding	No experimental manipulations of mice were performed. The study design was observational (known samples collected at different time

Blinding	points) and did not have experimental or control groups. Additionally, data collection and analysis could not be and were not performed blind to the conditions of the experiments.

Reporting for specific materials, systems and methods

We require information from authors about some types of materials, experimental systems and methods used in many studies. Here, indicate whether each material, system or method listed is relevant to your study. If you are not sure if a list item applies to your research, read the appropriate section before selecting a response.

Materials & experimental systems

n/a	Involved in the study	
☒	☐	Antibodies
☒	☐	Eukaryotic cell lines
☒	☐	Palaeontology and archaeology
☐	☒	Animals and other organisms
☒	☐	Clinical data
☒	☐	Dual use research of concern
☒	☐	Plants

Methods

n/a	Involved in the study	
☒	☐	ChIP-seq
☒	☐	Flow cytometry
☒	☐	MRI-based neuroimaging

Animals and other research organisms

Policy information about studies involving animals; ARRIVE guidelines recommended for reporting animal research, and Sex and Gender in Research

Laboratory animals	C57BL/6NTac mice were used throughout. We collected samples from Pl4 (n=2), P21 (n=2), P28 (n=2), and P56 (n=6) mice. Mice were housed in groups of three to four per cage with a 12hr light/12hr dark cycles and ad libitum access to food and water. Ambient temperature and humidity were centrally regulated.
Wild animals	This study did not involve wild animals
Reporting on sex	All male samples were used for this study and the reported results are all derived from the male sex. This was done to avoid sex as a covariate in the analysis and reduce the experimental cost of requiring more replicates per sample.
Field-collected samples	The study did not involve samples collected from the field
Ethics oversight	All experiments were approved by the Institutional Animal Care and Use Committee of Weill Cornell Medicine and were in accordance with the 2011 Eighth Edition of the National Institute of Health Guide for the Care and Use of Laboratory Animals.

Note that full information on the approval of the study protocol must also be provided in the manuscript.

Plants

Seed stocks	*Report on the source of all seed stocks or other plant material used. If applicable, state the seed stock centre and catalogue number. If plant specimens were collected from the field, describe the collection location, date and sampling procedures.*
Novel plant genotypes	*Describe the methods by which all novel plant genotypes were produced. This includes those generated by transgenic approaches, gene editing, chemical/radiation-based mutagenesis and hybridization. For transgenic lines, describe the transformation method, the number of independent lines analyzed and the generation upon which experiments were performed. For gene-edited lines, describe the editor used, the endogenous sequence targeted for editing, the targeting guide RNA sequence (if applicable) and how the editor was applied.*
Authentication	*Describe any authentication procedures for each seed stock used or novel genotype generated. Describe any experiments used to assess the effect of a mutation and, where applicable, how potential secondary effects (e.g. second site T-DNA insertions, mosiacism, off-target gene editing) were examined.*
